# SimFFPE and FilterFFPE: improving structural variant calling in FFPE samples

**DOI:** 10.1093/gigascience/giab065

**Published:** 2021-09-22

**Authors:** Lanying Wei, Martin Dugas, Sarah Sandmann

**Affiliations:** Institute of Medical Informatics, University of Münster, Münster 48149, Germany; Institute of Medical Informatics, University of Münster, Münster 48149, Germany; Institute of Medical Informatics, Heidelberg University Hospital, Heidelberg 69120, Germany; Institute of Medical Informatics, University of Münster, Münster 48149, Germany

**Keywords:** FFPE, next-generation sequencing, artifact removal, structural variant calling

## Abstract

**Background:**

Artifact chimeric reads are enriched in next-generation sequencing data generated from formalin-fixed paraffin-embedded (FFPE) samples. Previous work indicated that these reads are characterized by erroneous split-read support that is interpreted as evidence of structural variants. Thus, a large number of false-positive structural variants are detected. To our knowledge, no tool is currently available to specifically call or filter structural variants in FFPE samples. To overcome this gap, we developed 2 R packages: SimFFPE and FilterFFPE.

**Results:**

SimFFPE is a read simulator, specifically designed for next-generation sequencing data from FFPE samples. A mixture of characteristic artifact chimeric reads, as well as normal reads, is generated. FilterFFPE is a filtration algorithm, removing artifact chimeric reads from sequencing data while keeping real chimeric reads. To evaluate the performance of FilterFFPE, we performed structural variant calling with 3 common tools (Delly, Lumpy, and Manta) with and without prior filtration with FilterFFPE. After applying FilterFFPE, the mean positive predictive value improved from 0.27 to 0.48 in simulated samples and from 0.11 to 0.27 in real samples, while sensitivity remained basically unchanged or even slightly increased.

**Conclusions:**

FilterFFPE improves the performance of SV calling in FFPE samples. It was validated by analysis of simulated and real data.

## Background

For decades, formalin fixation and paraffin embedding (FFPE) has been widely used to prepare and preserve biopsy specimens [1]. FFPE tissues preserve morphological and cellular details and provide a method for long-term storage at room temperature. These advantages make FFPE tissues the most common sources of archived clinical material: it is estimated that >400 million FFPE samples are currently available, many of which have corresponding clinical records, including diagnoses, treatment options, and drug responses [1]. Furthermore, rare tumors are most often stored as FFPE samples [2]. Therefore, FFPE samples provide a common and valuable source for medical research.

Next-generation sequencing (NGS) plays an important role in medical research. It allows us to investigate entire genomes, uncover the molecular characteristics of diseases, and provide insights into therapies. However, formalin fixation can result in fragmented, degraded, protein cross-linked DNA, introducing false-positive results to NGS data analysis [3]. The interpretation of NGS data strongly relies on bioinformatics tools; therefore, to analyze FFPE samples, these tools need to be optimized to minimize the number of false-positive or false-negative results.

NGS can be used to detect genomic variants of different scales: single-nucleotide variants (SNVs), short insertions/deletions, and structural variants (SVs), including copy number variants (CNVs). So far, studies on FFPE-specific artifacts have been focusing on false-positive SNVs and only a few on CNVs [3]. When performing variant calling on FFPE samples, we observed a large number of false-positive SVs. However, to our knowledge no study has yet considered filtration of these calls. Artifact chimeric reads (ACRs) are known to be enriched in FFPE samples [4] and are likely leading to false-positive SV calls. It is hypothesized that these ACRs are derived from the binding of single-stranded DNA (ssDNA) fragments [4]. The proportion of ssDNA is much higher in FFPE samples than in fresh frozen (FF) samples because double-stranded DNA (ds-DNA) is denatured owing to the high temperature used in the deparaffinization and reverse cross-linking steps for DNA extraction from FFPE samples [4, 5]. These ssDNAs may randomly self-assemble if short reverse complementary (SRC) regions exist. During the end-repair step of library construction, T4 DNA polymerase removes the 3′ overhangs and fills in the 5′ overhangs of the binding product [4], thereby producing artifact chimeric ds-DNA, which eventually leads to false-positive SV calls (see illustration in Supplementary Fig. S1).

To evaluate and improve the performance of SV calling algorithms in FFPE samples, ground truth data are needed. However, publicly available real-world FFPE data sets with matched FF samples are scarce. Furthermore, to our knowledge, no experimental validation of SV candidates is available for these data sets. Therefore, we simulated data with known biological truth and performed expert-based validation of SV calls for 2 real data sets with FFPE and matched FF samples.

Aiming at improving SV calling performance in FFPE samples, we defined the following research objectives: (i) To develop an NGS read simulator that can specifically simulate ACRs in FFPE samples; the simulated reads should be as realistic as possible. (ii) To develop a tool that successfully removes ACRs while keeping non-artifact chimeric reads resulting from real SVs. (iii) To benchmark existing SV callers by using simulated as well as real NGS data sets resulting from FFPE samples, and to evaluate the effect of ACR removal on SV calling.

## Methods

### Data sets

#### Real data sets

Two real-world data sets were analyzed in this study. Both contain whole-exome sequencing (WES) data of FFPE and matched FF samples publicly available at the European Nucleotide Archive (Supplementary Table S1). The first data set contains 13 FFPE breast tumor samples and 13 corresponding FF samples (Accession No. SRP044740). The second data set contains 5 FFPE samples with unspecified tumor type and 4 corresponding FF samples (Accession No. PRJNA301548; note: 2 FFPE samples belong to the same patient).

#### Simulated data sets

The real data available to us are all WES data; however, the ideal data for SV calling are whole-genome sequencing (WGS) data with sufficient read length. Therefore, to complement the available real data, we generated simulated data that are more optimal for SV calling (mimicking WGS data with 150 bp read length). To generate simulated data sets, we first simulated 400 non-overlapping SVs with varying lengths (1–10 kb; 100 duplications, 100 deletions, 100 inversions, and 100 translocations) on chromosome 12 of genome assembly hg19 using RSVSim [6]. Next, we applied SimFFPE (algorithm described in section “Simulating ACRs with SimFFPE”) to the mutated, as well as the original, chr12 sequence to generate simulated FFPE NGS reads that cover the whole chromosome. Notably, the 100 translocations were simulated as large insertions of random segments from other chromosomes. To evaluate the effect of FilterFFPE (algorithm described in section “Filtering ACRs with FilterFFPE”) on SV calling, we generated 3 simulated data sets (see Table 1). Altogether, 41 samples were simulated.

**Table 1.** Characteristics of the simulated data sets

Name	n	Coverage (×)	SV frequency (%)	Proportion of ACFs (%)
Sim1	10	10–100	50	10
Sim2	10	50	10–100	10
Sim3	21	50	50	0–20

### Simulating ACRs with SimFFPE

**Figure 1:**
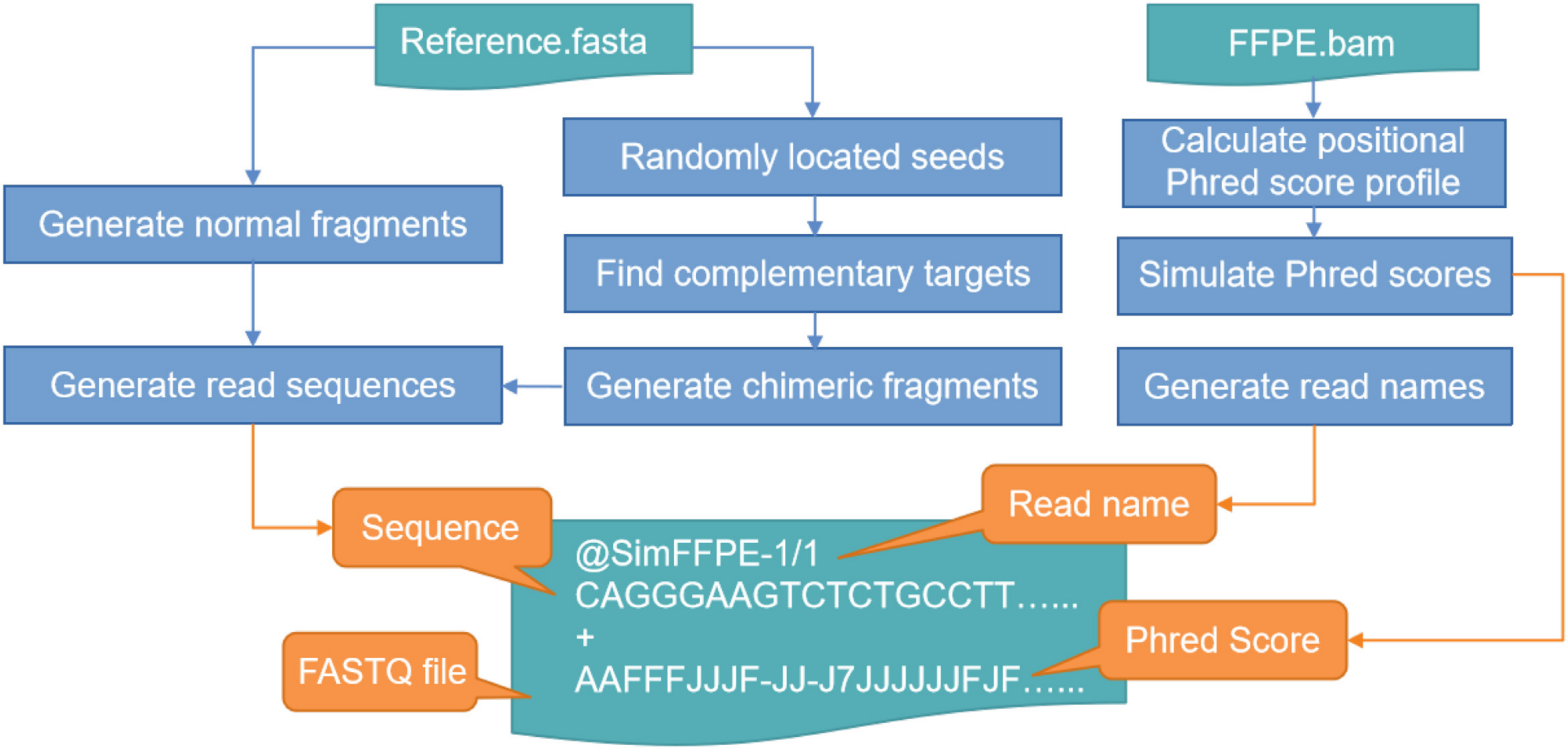
: Workflow of SimFFPE. The algorithm generates normal and artifact chimeric fragments and simulates read sequences from these fragments. Phred scores are simulated on the basis of read position. A FASTQ file is generated as output.

The general workflow of SimFFPE is shown in Fig. 1.

The whole simulation can be split into 2 parts—the simulation of normal fragments and the simulation of artifact chimeric fragments (ACFs). While normal fragments are simulated directly from the reference genome, the simulation of ACFs is more complex. Details of ACF simulation are described in the subsection “Simulating ACFs.” We observed normally distributed fragment lengths in real data; therefore, we used a normal distribution to simulate fragment lengths. This observation is in line with several other publications on NGS simulators (e.g., [7, 8]).

Simulations for WGS, as well as WES and targeted sequencing data, are supported. For WES and targeted data, we uniformly model the capture efficiency. Simulated read sequences are generated from one end (single-end sequencing) or both ends of the fragments (paired-end sequencing). We refer to the reads generated from ACFs as ACRs. It should be noted that well-known errors in NGS data, such as base substitutions and indels, are not the focus of this work; therefore, SimFFPE performs only simple random error simulations.

Phred quality scores are correlated with base position in the reads [8]. Accordingly, SimFFPE estimates positional Phred score profiles from real NGS data for simulations. We provide 2 exemplary positional Phred score profiles for read lengths of 100 and 150 bp.

#### Simulating ACFs

To simulate ACFs, the essential task is to find genome sequence pairs with SRC regions and combine them to form double-stranded fragments. A graphic representation of this process is available in Supplementary Fig. S1.

To locate candidate SRC pairs for binding, we randomly select short (on average 6 bp) genome sequences (referred to as “seed sequences”) and find their reverse complementary sequences (referred to as “target sequences”). The obvious match—target sequences at the same genomic location on the reverse strand—are excluded.

**Figure 2:**
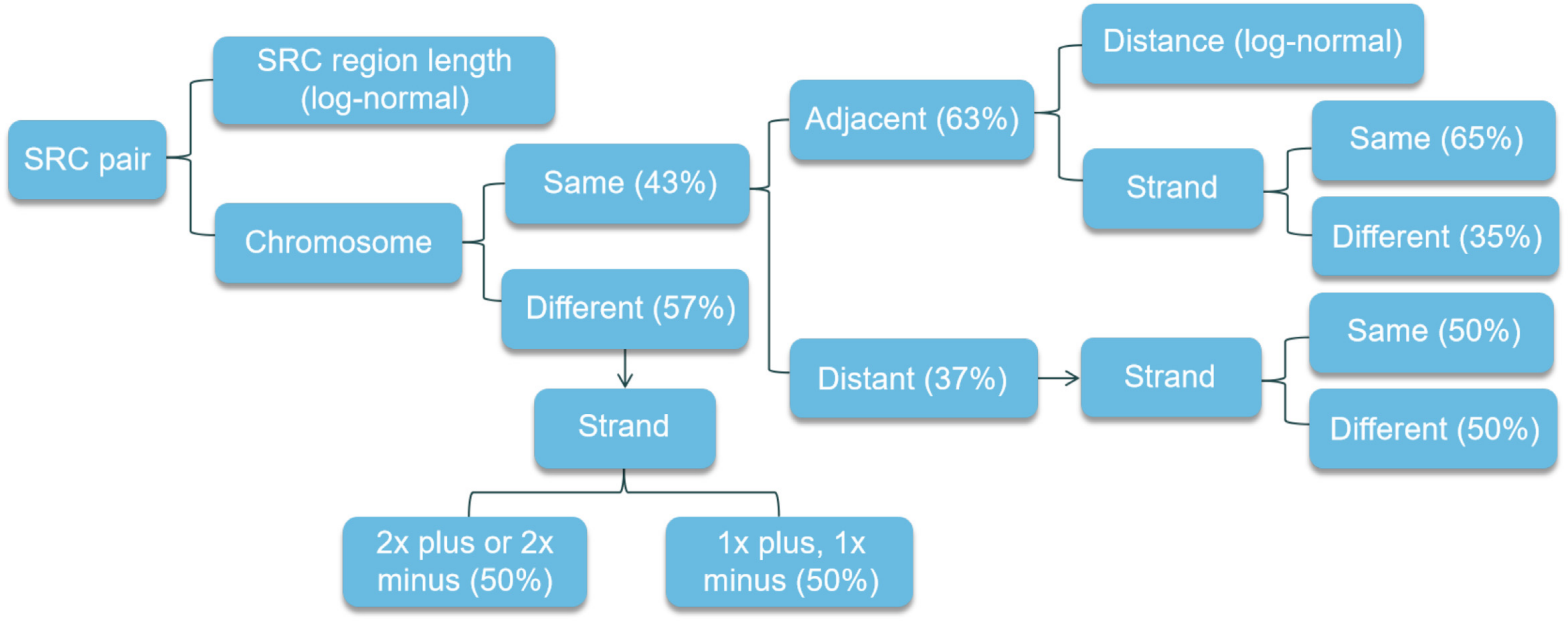
: Aspects that are considered when simulating short reverse complementary (SRC) pairs. The proportions and distribution models shown in parentheses are SimFFPE’s default settings, which are determined on the basis of 18 real FFPE samples from 2 public data sets.

For a given seed sequence, there can be millions of candidate target sequences. If 1 target sequence were randomly selected, this could result in simulated data widely deviating from real data. To simulate data as realistically as possible, an elaborate set of characteristics is considered when simulating SRC pairs. Among others, these characteristics include SRC region length distribution, location (whether from the same chromosome and, if so, whether from adjacent chromosomal regions), distance, and strand (Fig. 2). All default distributions and proportions of SimFFPE are based on the characteristics of the 18 real FFPE samples from the 2 aforementioned public data sets (Supplementary Section S3). Based on the SRC region length distribution in these samples, we decided to use a lognormal distribution (μ = 1.8, σ = 0.55) to approximate the true distribution. More information about the relevant parameters can be found in the vignettes and reference manual of the SimFFPE package [9].

Because only 1 target sequence of a seed is finally selected, computational costs are greatly reduced by SimFFPE identifying target sequences of a seed only within a small region. More specifically, we partition the genome into small windows (5 kb). Target sequences are searched in a random window or within the same window of the seed. The resulting SRC pairs and ACFs are called between-window SRC pairs and distant ACFs, and within-window SRC pairs and adjacent ACFs, respectively.

The reason to differentiate between these 2 cases is as follows: we observed that in real FFPE samples, ∼27% (43% on the same chromosome × 63% from adjacent chromosomal regions) of ACFs are derived from the binding of adjacent (within 5 kb) SRC pairs (Supplementary Figs S5 and S6). Owing to the small window size and the sheer human genome size (>3 Gb), such a relatively high proportion indicates a high chance of binding between 2 adjacent ssDNAs. It seems reasonable to assume that 2 ssDNAs originating from adjacent genomic regions are, on average, physically closer to each other and thus have a higher chance of binding. Accordingly, we divide the ACF simulation into 2 parts: the adjacent ACF simulation and the distant ACF simulation. For both, several demands have to be met.

**Figure 3:**
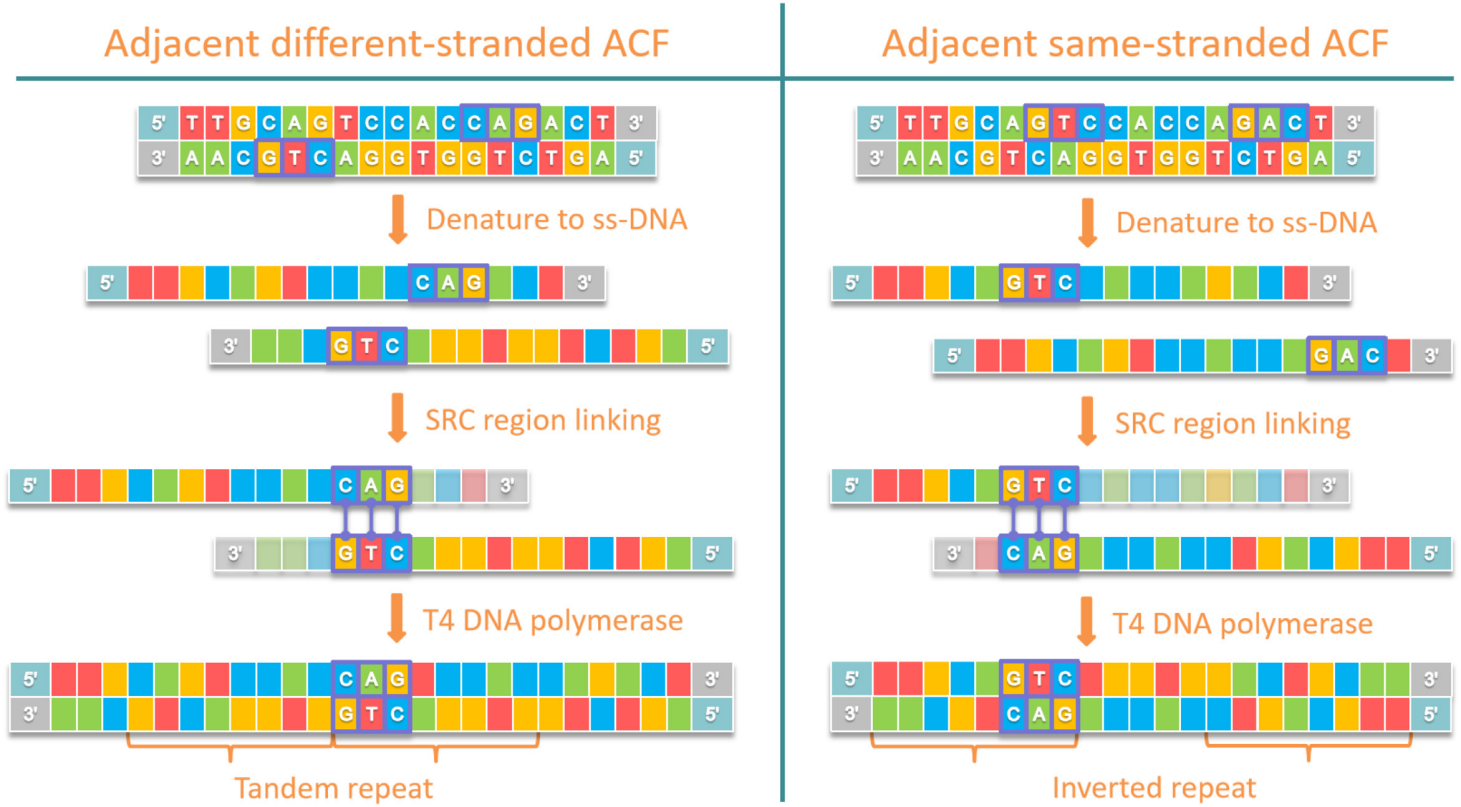
: Examples of forming a repeat and an inverted repeat by adjacent ssDNA combination. ACF: artifact chimeric fragment; SRC: short reverse complementary.

The most important considerations for adjacent ACF simulation are as follows (details in Supplementary Figs S7–S9): (i) In real data, we observed a relatively high proportion of adjacent ACFs resulting from genomically close SRC pairs (50–200 bp). Analyzing the distribution of the distance between the combined SRC pair in real data, we decided to choose a lognormal distribution (μ = 4.7, σ = 0.35) for simulation because this closely resembles real data. (ii) One SRC pair may originate from different strands of DNA or from the same strand. The probabilities for these 2 cases are not equal. We observed a higher proportion of same-stranded (65%) versus different-stranded (35%) SRC pairs in adjacent ACFs in real data. A corresponding parameter (sameStrandProp; default = 0.65) was set. It seems possible that a long ssDNA molecule might form a hairpin structure and generate chimeric ds-DNA. This might explain the higher proportion of same-stranded SRC pairs in adjacent ACFs. (iii) In some real samples, we observed that some read pairs from adjacent ACFs both align to the same genomic locus (Supplementary Fig. S9). We found out that this occurs when enzymatic fragmentation is used in library preparation. Enzymes are able to recognize and cut at specific sites of the genome. As shown in Fig. 3, an adjacent ACF can be a repeat or an inverted repeat of a DNA sequence. Thus, enzymatic fragmentation leads to both ends of the ACF being cut at the same genomic locus. If the ACF is an inverted repeat and is enzymatically fragmented (with both sides ending at the same genomic position), then the read pair are sequenced from the same starting point and proceed with the same sequence (until the end of the repeat unit). As a result, this read pair is mapped to the same genomic locus. Accordingly, SimFFPE supports the simulation of enzymatic fragmented adjacent ACFs. When enzymatic fragmentation is simulated, SimFFPE cuts the adjacent ACF (if it is a repeat) at a random site in one end, and cuts the other end at the same genomic locus of the repeated sequence. For simplicity, we did not model the specific cutting sites of enzymes, but we ensured that the fragment length distribution was still well simulated.

For distant ACF simulation, some additional aspects have to be taken into account: (i) Analysis of the real data sets indicates that strand usage for the formation of distant ACFs is almost equal. (ii) In real data sets, we observed a common feature across the whole genome: within some small genomic regions (1-2 kb), there were more ACRs originating from distant ACFs compared to other regions. These are referred to as “spikes” (Supplementary Fig. S9). To simulate these spikes, we use a β-distribution (α = β = 0.5) to model the amount of distant ACRs in each small region. Thus, the simulation enriches distant ACRs in some of these small regions.

A summary of the differences in simulating adjacent and distant ACFs is provided in Table 2.

**Table 2.** Differences in simulating adjacent and distant ACFs

Parameter	Adjacent ACF	Distant ACF
SRC pair	Within-window	Between-window
Strand usage of the SRC pair	Unequal	Equal
Distance between the SRC pair	Lognormal	Random
Enzymatic fragmentation simulation	Applicable	
Spike simulation		Applicable

### Filtering ACRs with FilterFFPE

**Figure 4:**
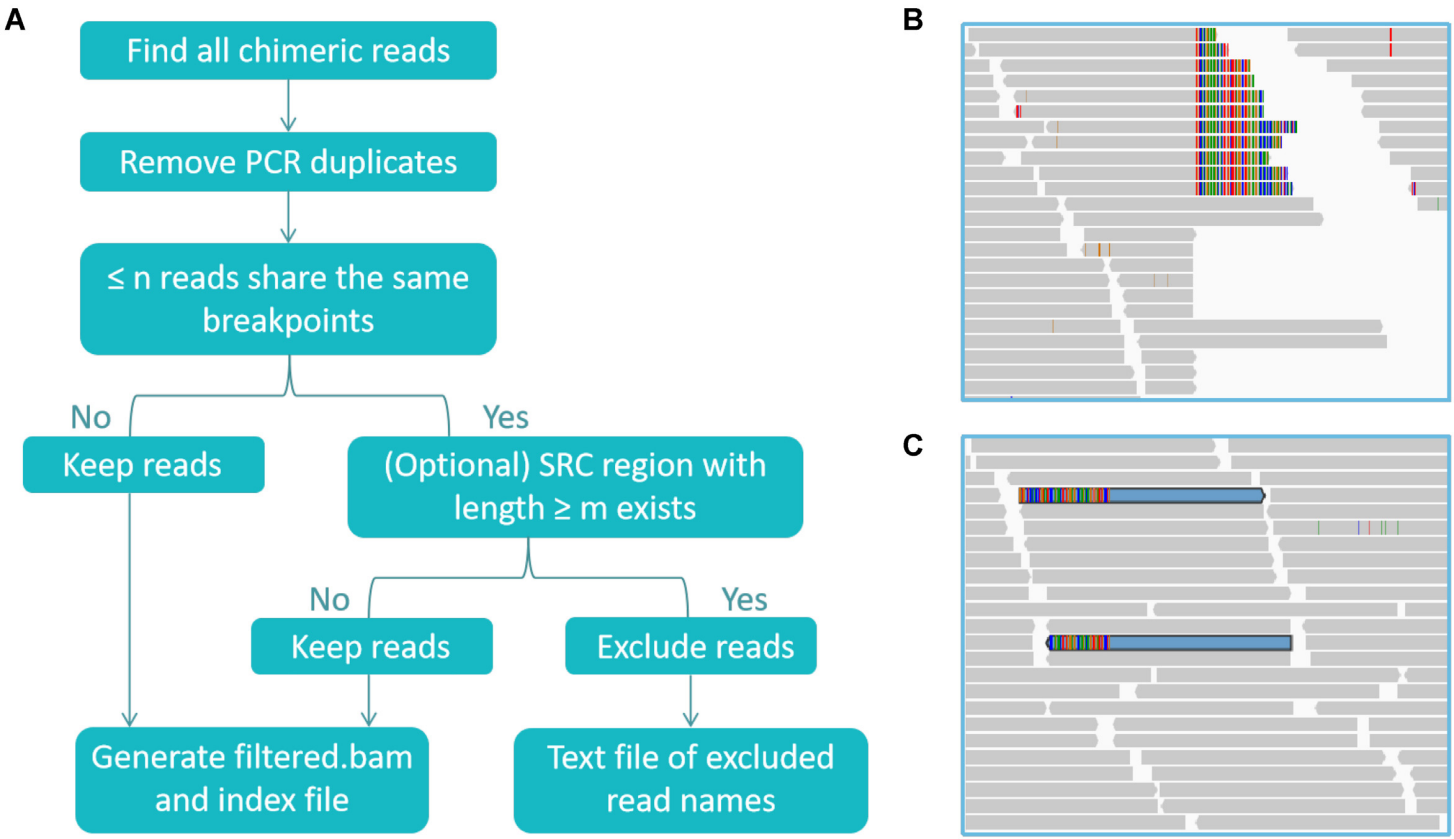
: Filtration with FilterFFPE. (A) The workflow of FilterFFPE to filter out artifact chimeric reads (ACRs). Values of n and m are user-definable. (B) Breakpoint of a true deletion. (C) Breakpoint of an ACR pair. SRC: short reverse complementary.

The workflow of FilterFFPE is shown in Fig. 4. To filter ACRs while preserving informative chimeric reads resulting from true SVs, it is important to identify features that can help to distinguish between these 2 types of chimeric reads. As long as sequencing depth and SV frequency are not too low, >1 or even dozens of chimeric fragments can cover the same breakpoints of the SV event (Fig. 4B). However, because the binding of 2 ssDNAs is a rather random event, there is little chance that the breakpoint of an ACF is also present in other ACFs or in chimeric reads from real SV events (note that for paired-end sequencing, the read pair from an ACF can share the same breakpoints, but both reads belong to the same fragment). Therefore, an apparent filtering strategy is to evaluate the number of reads sharing the same breakpoint: if n or fewer chimeric reads (default: n = 2 for paired-end sequencing) share the same breakpoints, these reads are recognized as potential ACRs. Notably, chimeric reads covering true SVs may also be identified as potential ACRs at low sequencing depths or low SV frequencies. Therefore, we need an additional feature to validate these potential ACRs. This feature is the presence of an SRC region. According to the ACF formation mechanism, only the presence of an SRC region causes 2 ssDNAs to bind and form an ACF. Therefore, FilterFFPE analyzes whether an SRC region is present next to the breakpoint of an ACR candidate.

The detection of an SRC region is based on the main characteristics of ACRs. ACRs contain 2 genome segments: 1 from the seed sequence and 1 from the complementary target. Thus, there exist (at least) 2 alignments, both containing soft-clipped bases. In an ACR, towards the end of the mapped sequences, a short region should be mapped in both alignments. This region can be identified as the SRC region that links 2 ssDNAs forming the ACF. First, FilterFFPE identifies potential ACRs. Second, the presence and lengths of SRC regions within these ACR candidates is analyzed. Only reads with plausible SRC regions (SRC region length ≥ m, with default m = 1 to remove as many ACRs as possible) are removed by FilterFFPE. This step helps not to exclude real chimeric reads resulting from low-coverage regions or low-frequency SVs by mistake, i.e., preserving sensitivity. However, sequencing noise in ACRs may harm the correct detection of SRC regions. Thus, it is possible that some ACRs are falsely categorized as real chimeric reads, i.e., positive predictive value (PPV) is decreased. Therefore, this second filtration step is optional.

After determining the reads to be excluded, FilterFFPE generates a filtered and indexed BAM file, as well as a text file containing the names of the excluded reads.

### Performance evaluation

**Figure 5:**
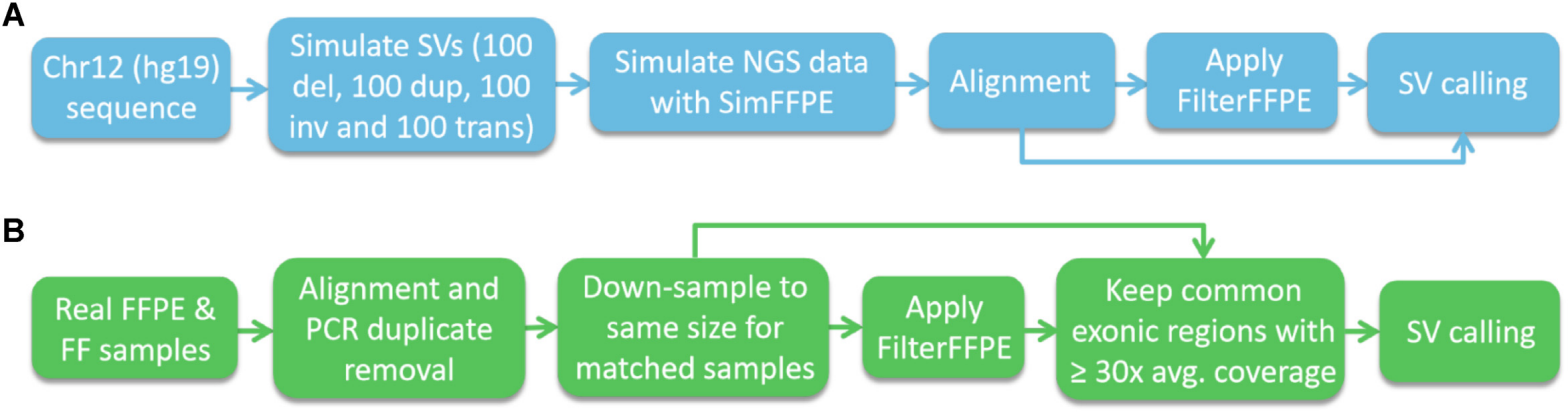
: Performance evaluation of SV callers Delly, Lumpy, and Manta with and without application of FilterFFPE considering real and simulated FFPE samples. Steps performed in case of (A) simulated data and (B) real data are visualized. del: deletions; dup: duplications; FF: fresh frozen; FFPE: formalin-fixed paraffin-embedded; inv: inversions; SV: structural variant; trans: translocations.

The steps taken to evaluate SV calling performance in real and simulated FFPE samples with and without application of FilterFFPE are shown in Fig. 5 (see Supplementary Section S4 for information on sequence alignment, duplicate removal, downsampling, and so forth). Three SV callers, Delly (v0.7.9) [10], Lumpy (v0.3.1) [11], and Manta (v1.6.0) [12], were used for SV calling. These tools have performed best in recent benchmarking studies [13–15].

For real data sets, each pair of matched FFPE and FF samples was downsampled to the same size. Furthermore, only reads within exonic regions with sufficient coverage were used for SV detection (exonic regions with mean coverage ≥30× in both samples of the pair). PPV, sensitivity, and F1-score were used to evaluate each tool's SV calling performance with and without application of FilterFFPE.

Different SV callers can detect the same breakpoint with minor shifts in the genomic location. To determine whether an SV call indicates a true-positive SV and whether it is shared between 2 samples, a maximum shift of ±5 bp is allowed to identify consistent breakpoints. This threshold was determined on the basis of a previous evaluation on different SV callers’ breakpoint resolution by Gong et al. [14].

Because data on experimental validation of SV candidates in real samples were not available, we performed expert-based validation by 2 independent experts in the field of SV detection (see Supplementary Section S5 for characteristics used to determine true-positive SVs). To facilitate manual inspection, we divided SV calls in FFPE samples (before and after FilterFFPE’s application) into 4 categories: (1) SV calls with high probability of being true-positive SVs (1,041 SV calls). These are shared SV calls with matched FF samples (without application of FilterFFPE in FF samples). (2) SV calls with reduced probability of being true-positive calls (2,282 SV calls). These are non-shared SV calls with reliable support (shared with ≥1 non-matched FF sample, or having in total ≥10 reads of split- and/or paired-read support). The criteria for reliable support were determined on the basis of prior manual inspection of 500 randomly selected non-shared SV calls. (3) SV calls with low probability of being true-positive calls (1,952 SV calls). These are non-shared SV calls that lacked reliable support (do not match a call in any FF sample and have <10 supporting reads) but showed characteristics that we identified in categories 1 and 2 as being typical for true-positive variants (matching a call that is labeled as true positive in any other FFPE sample, or overlapping with a gene that is characterized by a high number of SV calls). (4) SV calls with high probability of being false-positive calls (the remaining 44,877 SV calls).

We manually inspected all 5,275 SV calls in categories 1–3. The SV calls in the fourth category were automatically labeled as false-positive calls because they lack characteristics of potentially true variants. To ensure that this automatic classification was reliable, we randomly selected 2,000 of the 44,877 calls and performed manual inspection. In total, 1,996 of these 2,000 calls are false-positive calls; the remaining 4 are ambiguous (and remain so after application of FilterFFPE; for details see Supplementary Section S5). We therefore consider it plausible to automatically label the whole category as false-positive calls. In total, we labeled 1,506 SV calls as true-positive and 47,026 as false-positive calls. In addition, 1,620 SV calls could not be classified clearly and were thus excluded from further evaluation. The number of SV calls with information on initial category and final judgment is shown in Supplementary Table S2.

## Results

### Simulating realistic FFPE data using SimFFPE

A comparison of SimFFPE to existing NGS data simulators is provided in Table 3. The other tools mainly serve to simulate read-level sequencing noise. In contrast, SimFFPE additionally simulates ACFs that are characteristic of FFPE samples. These ACFs are fragment-level noise that can lead to false-positive SV calls.

**Table 3.** Comparison of SimFFPE to existing NGS data simulators

Name	Installation	Language	OS	Sequencing type	Read type	Noise type
SimFFPE	Bioconductor, Bioconda	R	L, M, W	WGS, WES	SE, PE	ACR, SeqE
ART [7]	Bioconda, manual	C++, Perl	L, M, W	WGS	SE, PE	SeqE
BEAR [16]	Manual	Python, Perl	L	WGS	SE, PE	SeqE
FASTQSim [17]	Manual	Bash, Python	L	WGS	SE	SeqE
GemSim [18]	Manual	Python	L, M, W	WGS	SE, PE	SeqE
Grinder [19]	Manual	Perl	L, M, W	WGS	SE, PE	SeqE
InSilicoSeq [20]	Bioconda, manual	Python	L, M, W	WGS	PE	SeqE
NeSSM [21]	Manual	Python	L	WGS	SE, PE	SeqE
pIRS [22]	Bioconda, manual	C++, Perl	L	WGS	PE	SeqE
SimuSCoP [8]	Manual	C++	L	WGS, WES	SE, PE	SeqE
SInC [23]	Manual	C++	L	WGS	PE	SeqE

ACR: artifact chimeric read; L: Linux; M: MacOS; OS: operating system; PE: paired-end sequencing; SE: single-end sequencing; SeqE: sequencing error; W: Windows; WES: whole-exome sequencing; WGS: whole-genome sequencing.

**Figure 6:**
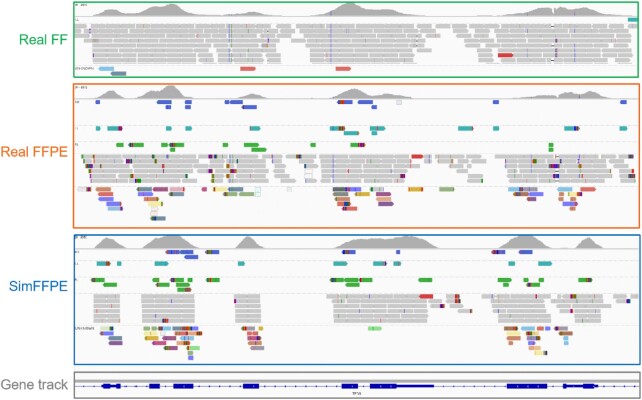
: Exemplary alignment of reads simulated by SimFFPE in comparison to real reads in matching FF and FFPE samples. Soft-clipped bases are shown. Alignments are grouped by pair orientation. Pair orientation is presented in terms of read-strand: left (L) vs right (R), and first read vs second read of a pair. The color (not gray) of the alignment indicates an abnormal pair orientation, or a different chromosome that the paired read mapped to. Alignments with normal pair orientation are colored in grey. FF: fresh frozen; FFPE: formalin-fixed paraffin-embedded.

Figure 6 shows exemplary aligned reads generated by SimFFPE. For comparison, data from a real sample (FFPE and matching FF) are displayed. SimFFPE generates ACRs that closely resemble those highly noisy reads in real FFPE samples. In contrast, existing simulators such as ART [7] only produce normal reads similar to those in FF samples (Supplementary Fig. S9). We further compared the proportion of abnormally paired reads in real and simulated samples (Supplementary Section S7). The proportion of abnormally paired reads is higher in real FFPE samples than in FF samples. The distribution of this proportion in simulated data set Sim3 (with varying artifact levels) is very close to that of real FFPE samples.

### Filtering FFPE-specific ACRs with FilterFFPE

On all real and simulated samples, we performed filtration with FilterFFPE (using default setting with 2-step filtration; for results on filtration with FilterFFPE applying the first step only, see Supplementary Sections S8–S11).

For each simulated sample, excluded reads could be divided into ACRs and normal reads (tagged by SimFFPE when generating the data) and counted separately (Supplementary Fig. S13). As a result, in 40 of 41 simulated samples (1 sample without any ACFs was simulated), 99.73–100% of the removed reads were ACRs (mean: 99.96%, see Supplementary Fig. S14, results with 2-step filtration). These excluded ACRs account for 97.72–97.94% (mean: 97.82%) of all chimeric reads derived from ACFs (Supplementary Fig. S16). Reads obtained from ACFs can also be non-chimeric (without supplementary alignment): these include reads that do not cover breakpoints or cover only a few bases of 1 of the 2 original sequences (see Supplementary Fig. S15 for illustration). These non-chimeric reads do not lead to artifact split-read support and are thus not removed by FilterFFPE. Therefore, the percentage of excluded ACRs based on all reads from ACFs is 65.63% on average (Supplementary Fig. S17).

In real data sets, we applied FilterFFPE to both FFPE and matched FF samples. The percentage of filtered reads ranged from 0.33% to 9.2% in FFPE samples (median: 2.5%). In contrast, only 0.015–0.33% (median: 0.10%) were filtered in FF samples. These results match our previous observation that ACRs are enriched in FFPE samples compared to FF samples. It should be noted that FF samples are expected to contain some ACRs because any heating step during sequencing can result in DNA denaturation and thus ACR generation. Nevertheless, because the percentage of ACRs in FF samples is low, the effect of these ACRs on SV calling is usually negligible (Supplementary Fig. S23).

### Evaluation of SV calling with and without previous filtration with FilterFFPE

**Figure 7:**
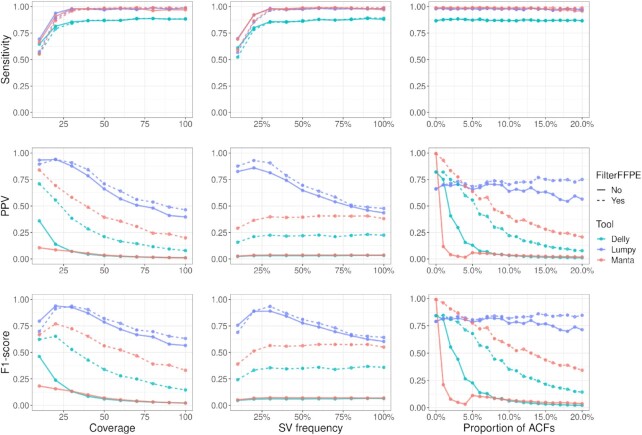
: FilterFFPE increases positive predictive value (PPV) of SV calling in simulated samples while keeping sensitivity unchanged. Lumpy performs best—with and without application of FilterFFPE.

Figure 7 shows the performance of the 3 SV calling tools Delly, Lumpy, and Manta on the 3 simulated data sets with and without previous application of FilterFFPE. Results show that FilterFFPE substantially improves PPV of SV calling, considering a diverse set of scenarios, while only affecting sensitivity in a few exceptional cases. Thus, an overall improvement in F1-score is observed.

As simulated coverage or ACF proportion increases, the number of ACRs increases; therefore, we expected and also observe an increasing number of false-positive SV calls and decreasing PPV. SV frequency has no effect on the number of ACRs, and thus, we did not expect any effect on the number of false-positive SV calls. It can be observed that both Manta and Delly are characterized by stable PPV at different SV frequencies. Interestingly, Lumpy shows a decrease in PPV with increasing SV frequency. Detailed evaluation of the SV calling results revealed that Lumpy generated several SV candidates for real SVs with different breakpoints. Some of these SV candidates were recognized as false-positive calls because the detected breakpoints were not close enough to the real ones (±5 bp).

After removing ACRs with FilterFFPE, PPVs of all 3 tools increase in all our simulated data sets: Manta shows the largest increase (on average from 0.06 ± 0.15 [mean ± SD] to 0.45 ± 0.21), followed by Delly (0.10 ± 0.18 to 0.29 ± 0.22) and Lumpy (0.65 ± 0.13 to 0.71 ± 0.12).

Sensitivity of the 3 tools is stable across all simulated data sets, except for low coverage (≤30×) or low SV frequencies (≤30%). In these extreme cases, it is difficult to distinguish between real chimeric reads and ACRs. Therefore, application of FilterFFPE slightly reduces sensitivity (on average from 0.83 ± 0.13 to 0.78 ± 0.17; 6 samples). For all other samples, sensitivity even increases marginally after using FilterFFPE (on average from 0.94 ± 0.05 to 0.95 ± 0.05). Compared to the other tools, Delly is characterized by lowest sensitivity—before and after filtration with FilterFFPE. This is due to the fact that Delly did not detect translocations with precise genomic location: 61 of 100 simulated translocations could not be detected accurately by Delly (often with a deviation of 30–300 bp at the breakpoint).

It should be mentioned that these results are based on all reported SV calls. In addition, every tool has diverse internal categories to characterize SV calls of different qualities, including "precise" vs "imprecise" calls (whether breakpoints can be precisely located) and/or "pass" vs "non-pass" calls (whether certain quality conditions are met). Interestingly, with the combined use of these categories and FilterFFPE, the best performance is observed in case of FilterFFPE+Delly, considering only precise calls. Delly’s precise calls have a mean F1-score of 0.71 ± 0.14 across the 3 simulated data sets and reach 0.91 ± 0.06 with FilterFFPE. More details can be found in Supplementary Figs S18–S21.

**Figure 8:**
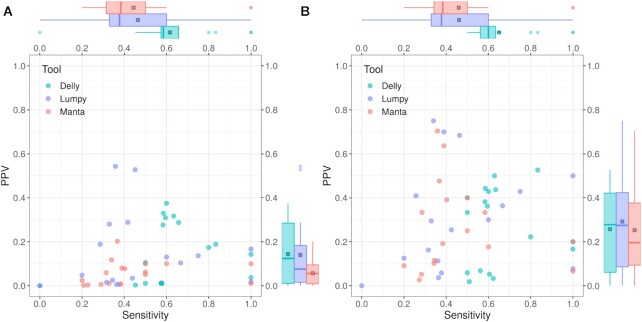
: FilterFFPE considerably increases positive predictive value (PPV) of SV calling in real FFPE samples, while slightly improving sensitivity. PPV and sensitivity of SV calling (A) before and (B) after application of FilterFFPE are shown. Each dot represents 1 FFPE sample. The upper and lower hinges of the box plot correspond to the third and the first quartiles, respectively. Box midline represents median value. Whisker extends to the largest or smallest value that is within 1.5 * interquartile range of the upper or lower hinge. Square in the box plot indicates mean value. Altogether, Lumpy is characterized by highest PPV and Delly by highest sensitivity.

Figure 8 shows the influence of FilterFFPE on SV calling in real FFPE samples. Similar to the results in simulated data sets, application of FilterFFPE leads to a considerable improvement in PPV and a minor improvement in sensitivity: filtration with FilterFFPE increases mean PPV in case of Delly from 0.14 ± 0.14 to 0.26 ± 0.19, from 0.06 ± 0.05 to 0.25 ± 0.21 for Manta, and from 0.14 ± 0.17 to 0.29 ± 0.25 for Lumpy; mean sensitivity increases from 0.62 ± 0.23 to 0.65 ± 0.16 for Delly, from 0.44 ± 0.24 to 0.46 ± 0.23 for Manta, and remains 0.46 for Lumpy. For Delly and Manta, more true-positive calls were exclusively detected after application of FilterFFPE (Supplementary Tables S3 and S4), thus resulting in increased sensitivity. Considering the tools’ internal categories, the best overall performance can be observed—just like in the case of simulated data—for FilterFFPE+Delly, considering only precise calls. Delly’s precise calls have a mean F1-score of 0.45 ± 0.28 in real FFPE samples and reach 0.58 ± 0.24 with FilterFFPE. More details can be found in Supplementary Fig. S22.

To further validate the performance of FilterFFPE, we also calculated the number of reported SV calls in FF samples before and after FilterFFPE’s application (Supplementary Fig. S23). Over all 18 real FFPE samples, FilterFFPE reduces the number of reported SV calls by 44% (Delly), 76% (Manta), and 61% (Lumpy). In comparison, the number is reduced by only 0.3% (Delly) and 5% (Manta) and increased by only 0.2% (Lumpy) in matched FF samples.

## Discussion

In this article, we introduce 2 R packages for improved handling of sequencing data generated from FFPE samples: SimFFPE and FilterFFPE. SimFFPE is a novel tool simulating realistic sequencing data from FFPE samples. Simulated data with known biological truth are the prerequisite for, e.g., optimization of variant calling pipelines. Based on the output of SimFFPE we developed and tested a new filtration algorithm for SV calling: FilterFFPE. Results on both simulated and real data show that our filtration algorithm is able to improve PPV without compromising the sensitivity of 3 established SV calling algorithms.

Despite developing a tool for realistic simulation of FFPE samples, it can be observed that the sensitivity of the 3 SV calling tools Manta, Delly, and Lumpy differed between simulated and real data. These discrepancies were mainly due to technical differences between these data sets: our simulated samples were whole-chromosome sequencing data (mimicking WGS data because they are the ideal material for SV calling) while real samples contained WES data and had a shorter read length (150 bp in simulated samples vs 90 bp in real samples).

The sensitivity of Lumpy and Manta was much lower for real data than for simulated data. Lumpy uses not only read-pair and split-read support but also read-depth support to identify SV candidates. However, regional coverage fluctuates heavily in WES data. Thus, it can harm read-depth support detection in Lumpy and lead to lower sensitivity. The reduced sensitivity of Manta is likely due to inaccurately detected SV positions. The accuracy of Manta’s local assembly might have been affected by the shorter read length of the real data. Delly showed the lowest sensitivity in simulated data sets but featured the highest in real data. It could be observed that Delly’s imprecise positioning of translocations leads to false-negative calls. In our simulated data, 25% (100 of 400) of all SVs were translocations, but only 2% (7 of 296) in real data.

Because the purpose of SimFFPE and the type of its simulated noise are different from those of existing simulators, it is difficult to compare SimFFPE with other simulation tools. However, exemplary comparison of simulated and real data in the IGV shows that reads generated by SimFFPE resemble real FFPE samples, while reads generated by other simulation tools resemble real FF samples.

It can be argued that for real data we do not know biological truth based on validation experiments but just by expert-based review. It is possible that our data contain misclassified variants, i.e., false-negative and false-positive calls. Nevertheless, the classification was based on a detailed scheme and criteria, and we performed careful manual inspection on >5,000 SV calls. Therefore, the effect of misclassified variants on our overall results can be assumed to be negligible.

Regarding FilterFFPE, the first filtration step may seem very similar to filtering out SV calls with split-read support ≤2. However, these 2 strategies are fundamentally different. Many true SV calls in real samples lack split-read support. For example, in the 18 real FFPE samples, 41% (615 of 1,506) of the true-positive SV calls had no split-read support. This can be related to the fact that real SVs often overlap with homologous sequences and/or sequence repeats [6]. These highly repetitive sequences can confound split-read alignment and thus obscure split-read support detection. Short read length and low coverage can also lead to missing split-read support. In addition, split-read support appears to be tool-dependent: for the same SV call we observe varying amounts of split-read support and missing information on how this number is calculated by each tool.

In real FFPE samples, we observed different levels of artifacts and, thus, variable levels of PPV for SV detection. One possible reason for sample-wise artifact level variation may be different time at high-temperature steps during processing of FFPE samples in the laboratory. For instance, larger paraffin blocks require longer time for deparaffinization, thus leading to a higher proportion of denatured DNA and a higher number of ACRs. Besides, long-term storage of FFPE samples leads to more fragmented DNA, which is more vulnerable to denaturation at high temperature. We also observed varying sensitivities for real samples, which could be explained by different levels of sample coverage.

The mechanism of the ACR generation in FFPE samples was first described in detail by Haile et al. [4]. They used S1 nuclease to remove ssDNAs before sequencing. Their method serves as a laboratory aid, while FilterFFPE serves as a bioinformatic method to deal with these ACRs. Using S1 nuclease has the advantage of preventing ACR generation from the source. It has the ability to degrade large amounts of ssDNA, especially in highly noisy FFPE samples. However, this can be unfavorable for low-frequency variant detection, especially considering that the FFPE material is often very precious. Moreover, the majority of already sequenced FFPE samples have not been treated with S1 nuclease. And currently, to our knowledge, S1 nuclease treatment is not yet widely adopted in the standard protocol for sequencing FFPE samples.

## Conclusion

SV calling in FFPE samples is challenging owing to the presence of ACRs leading to a large number of false-positive calls. To facilitate future development of FFPE-specific algorithms, we developed SimFFPE. It is the first simulation tool generating realistic NGS data from FFPE samples, simulating ACRs as well as normal reads. In addition, we developed the filtration algorithm FilterFFPE. Analyses on simulated as well as real data show that our algorithm successfully removes ACRs while keeping real chimeric reads. Thus, FilterFFPE improves PPV considerably without affecting sensitivity.

## Availability of Source Code and Requirements

Project name: SimFFPE and FilterFFPE

Project home page: https://bioconductor.org/packages/release/bioc/html/SimFFPE.html; https://bioconductor.org/packages/release/bioc/html/FilterFFPE.html

Operating system: Platform independent

Programming language: R

Other requirements: None

License: LGPL-3

RRID:SCR_021085; RRID:SCR_021086

## Data Availability

The 2 real data sets analyzed during the present study are available in the European Nucleotide Archive repository at https://www.ebi.ac.uk/ena/browser/home and can be accessed with accession Nos. SRP044740 and PRJNA301548.

The 3 simulated data sets can be generated with SimFFPE and RSVSim as described.

Additional supporting files including FilterFFPE’s outputs and tabular data are available from the *GigaScience* GigaDB database [24].

## Additional Files

**Supplementary Section S1**. Mechanism of ACF formation.

**Supplementary Section S2**. Real data sets.

**Supplementary Section S3**. Distributions and proportions for simulation.

**Supplementary Section S4**. BAM file processing.

**Supplementary Section S5**. Manual inspection.

**Supplementary Section S6**. IGV view of NGS data.

**Supplementary Section S7**. Proportion of abnormally paired reads.

**Supplementary Section S8**. FilterFFPE excludes FFPE-specific ACRs.

**Supplementary Section S9**. Evaluation of SV calling in simulated data sets.

**Supplementary Section S10**. Evaluation of SV calling in real data sets.

**Supplementary Section S11**. Increased sensitivity after application of FilterFFPE.

**Supplementary Figure S1**. Example of seed- and target sequences used to generate an SRC region in an ACF.

**Supplementary Figure S2**. SRC region length distribution in real FF samples.

**Supplementary Figure S3**. SRC region length distribution in real FFPE samples.

**Supplementary Figure S4**. SRC region length distribution in an exemplary sample simulated with SimFFPE.

**Supplementary Figure S5**. Proportion of SRC pairs with two ssDNA molecules originating from the same chromosome.

**Supplementary Figure S6**. Proportion of adjacent SRC pairs among same chromosomal SRC pairs.

**Supplementary Figure S7**. Proportion of same strand SRC pairs among adjacent SRC pairs.

**Supplementary Figure S8**. Cumulative distribution of the original genomic distance between two ssDNA molecules of adjacent SRC pairs.

**Supplementary Figure S9**. Exemplary alignment of reads simulated by SimFFPE and ART in comparison to real reads in an FFPE sample

**Supplementary Figure S10**. FilterFFPE removes artifact chimeric reads while keeping real chimeric reads.

**Supplementary Figure S11**. Proportion of improperly paired reads.

**Supplementary Figure S12**. Proportion of read pairs mapping to different chromosomes.

**Supplementary Figure S13**. FilterFFPE excludes FFPE-specific ACRs.

**Supplementary Figure S14**. Proportion of ACRs in excluded reads.

**Supplementary Figure S15**. Examples of chimeric and non-chimeric reads deriving from artifact chimeric fragments.

**Supplementary Figure S16**. Sensitivity of FilterFFPE excluding ACRs based on chimeric reads (with supplementary alignment) from ACFs.

**Supplementary Figure S17**. Sensitivity of FilterFFPE excluding ACRs based on all reads (chimeric + non-chimeric) from ACFs.

**Supplementary Figure S18**. SV calling performance for each SV call category in simulated data sets with and without FilterFFPE’s application (two filtration steps applied).

**Supplementary Figure S19**. SV calling performance for each SV call category in simulated data sets with and without applying FilterFFPE’s first filtering step only.

**Supplementary Figure S20**. SV calling performance for 6 simulated samples with low coverage or low SV frequency.

**Supplementary Figure S21**. SV calling performance for 35 simulated samples with high coverage and high SV frequency.

**Supplementary Figure S22**. SV calling performance for real data with (one- or two-step filtration) and without FilterFFPE’s application.

**Supplementary Figure S23**. Relative change in the number of SV calls in real data after application of FilterFFPE (one-step and two-step filtration).

**Supplementary Table S1**. Information on real data sets.

**Supplementary Table S2**. Number of SV calls with information on initial category and final judgment.

**Supplementary Table S3**. Number of true calls that are exclusively called before and after the application of FilterFFPE with one-step filtration.

**Supplementary Table S4**. Number of true calls that are exclusively called before and after the application of FilterFFPE with two-step filtration.

## Abbreviations

ACF: artifact chimeric fragment; ACR: artifact chimeric read; bp: base pairs; CNV: copy number variant; dsDNA: double-stranded DNA; FF: fresh frozen; FFPE: formalin-fixed and paraffin-embedded; Gb: gigabase pairs; IGV: Integrative Genomics Viewer; indel: short insertion/deletion; kb: kilobase pairs; NGS: next-generation sequencing; PCR: polymerase chain reaction; PPV: positive predictive value; SNV: single-nucleotide variant; SD: standard deviation; SRC: short reverse complementary; ssDNA: single-stranded DNA; SV: structural variant; WES: whole-exome sequencing; WGS: whole-genome sequencing.

## Competing Interests

The authors declare that they have no competing interests.

## Authors' Contributions

L.W. analyzed the data, developed SimFFPE and FilterFFPE, and wrote the manuscript. S.S. and M.D. guided the project and provided ideas for improvement during the development of the 2 tools, as well as suggestions on the manuscript. All authors read and approved the final version of the manuscript.

## Supplementary Material

giab065_GIGA-D-21-00120_Original_Submission

giab065_GIGA-D-21-00120_Revision_1

giab065_GIGA-D-21-00120_Revision_2

giab065_Response_to_Reviewer_Comments_Original_Submission

giab065_Response_to_Reviewer_Comments_Revision_1

giab065_Reviewer_1_Report_Original_Submission Michael Linderman -- 5/19/2021 Reviewed

giab065_Reviewer_1_Report_Revision_1 Michael Linderman -- 8/4/2021 Reviewed

giab065_Reviewer_2_Report_Original_Submission Binay Panda -- 6/16/2021 Reviewed

giab065_Reviewer_2_Report_Revision_1 Binay Panda -- 8/12/2021 Reviewed

giab065_Supplemental_File
