# Supplementary material for: SimFFPE and FilterFFPE: improving structural variant calling in FFPE samples
Source: Gigascience. 2021 Sep 22;10(9):giab065. doi: 10.1093/gigascience/giab065 (PMC8458033; doi:10.1093/gigascience/giab065)
Supplement: giab065_GIGA-D-21-00120_Revision_1 [file giab065_giga-d-21-00120_revision_1.pdf]

GigaScience

SimFFPE and FilterFFPE: improving structural variant calling in FFPE samples

--Manuscript Draft--

**Manuscript Number:**	GIGA-D-21-00120R1
**Full Title:**	SimFFPE and FilterFFPE: improving structural variant calling in FFPE samples
**Article Type:**	Technical Note
**Funding Information:**	
**Abstract:**	Background: Artifact chimeric reads are enriched in next-generation sequencing data generated from formalin-fixed paraffin-embedded (FFPE) samples. Previous work indicated that these reads are characterized by erroneous split-read support that is interpreted as evidence of structural variants. Thus, a large number of false positive structural variants is detected. To our knowledge, no tool is currently available to specifically call or filter structural variants in FFPE samples. To overcome this current gap, we developed two R packages: SimFFPE and FilterFFPE . Results: SimFFPE is a read simulator, specifically designed for next-generation sequencing data from FFPE samples. A mixture of characteristic artifact chimeric reads as well as normal reads is generated. FilterFFPE is a filtration algorithm, removing artifact chimeric reads from sequencing data, while keeping real chimeric reads. To evaluate the performance of FilterFFPE , we performed structural variant calling with three common tools (Delly , Lumpy and Manta) with and without prior filtration with FilterFFPE . After applying FilterFFPE , the average positive predictive value improved from 0.27 to 0.48 in simulated samples, and from 0.11 to 0.27 in real samples, while sensitivity remained basically unchanged or even slightly increased. Conclusions: FilterFFPE improves the performance of SV calling in FFPE samples. It was validated by analysis of simulated and real data.
**Corresponding Author:**	Lanying Wei<br>WWU Münster: Westfalische Wilhelms-Universitat Munster<br>Münster, GERMANY
**Corresponding Author Secondary Information:**	
**Corresponding Author's Institution:**	WWU Münster: Westfalische Wilhelms-Universitat Munster
**Corresponding Author's Secondary Institution:**	
**First Author:**	Lanying Wei
**First Author Secondary Information:**	
**Order of Authors:**	Lanying Wei
	Martin Dugas
	Sarah Sandmann
**Order of Authors Secondary Information:**	
**Response to Reviewers:**	Reviewer #1:<br>Specific comments/questions:<br>* Evaluating the fidelity of simulated NGS data in general is very difficult. And in this context many of the parameters you might compare in such an evaluation (i.e. those in Figure 2) were used to develop the tool. However, are there any other metrics you could use to quantitatively evaluate SimFFPE? For example, could a comparison between real and simulated FFPE data of the the fraction of improperly paired reads (or pairs mapping to different chromosomes) in tiled windows across the genome provide evidence of the fidelity of SimFFPE? Alternately are there experimental evaluations that might be possible, but would be beyond the scope of this work, which could be included as future work in the discussion?<br><br>Changes: We added section 7 to the supplementary material, describing our analysis

Powered by Editorial Manager® and ProduXion Manager® from Aries Systems Corporation

of the “Proportion of abnormally paired reads”, and refer to it at the end of subsection “Simulating realistic FFPE data using SimFFPE” of the main article.

We think it is a good suggestion to use these metrics to quantitatively evaluate SimFFPE. Results regarding this new analysis underline our previous observation that SimFFPE generates realistic simulated FFPE data.

* I had trouble understanding the manual curation process/results, and specifically category 3 SVs. My understanding is that this category comprises SVs that do not match a call in a FF sample and have less than 10 supporting reads. And that the authors manually reviewed 1,952 of these 46,829 SVs. Should I interpret Table S2 as 134/1,952 were actually true positives and 1,169/1,952 were considered ambiguous (and thus excluded)? Or is the "grey list" determined algorithmically? If the former, what fraction of the putative false positives (most of which, if I understood correctly, were not manually reviewed) would still be considered false positives (as opposed to "grey listed") had they been manually reviewed? Since there are so many putative false positives, it would seem that this category would have a significant impact on the results.

Changes: We revised our description in the main manuscript (section “Performance evaluation”) and supplement (section 5) and updated our categorization: now, there are four categories. Former category 3 is split into category 3 (low probability of being true; all calls manually reviewed) and category 4 (calls automatically labeled as false positives; random examples manually reviewed). Category 3 contains 1,952 calls, category 4 contains 44,877 calls. To access the fidelity of our automatic classification (category 4) we manually reviewed 2,000 randomly selected SV.

We totally agree that 44,877 putative false positive SV calls that are not reviewed could have a significant impact on the results. Therefore, we extended our manual review of variant calls and additionally considered 2,000 randomly selected calls in category 4. Among these, 1,996 are false positives and only 4 are considered ambiguous (located in homologous / repetitive regions). The 4 ambitious calls are kept after application of FilterFFPE. Our updated results confirm that the expected number of true positives within these 44,877 category 4 calls is very low (0-90). Therefore, we think it is reasonable to label the whole category 4 as false positives without further manual inspection.

* Page 5 and Figure S11: I find the absolute scales in Figure S11 panels a-c hard to interpret since there is no context to know how many ACRs should have been excluded. I would advocate for reporting the fraction of ACR and non-ACR reads excluded. This would complement the results on Page 5, which if I understood correctly, are effectively the precision/PPV ("99.73% to 100.00% of the filtered reads were ACRs (average: 99.96%)."). Is the sensitivity also reported somewhere? If not, I think including those stats in that paragraph would help the reader better understand FilterFFPE's effectiveness.

Changes: We added four new figures to our supplement showing PPV and sensitivity of FilterFFPE (Figures S14-S17). Additionally, we updated the subsection “Filtering FFPE-specific ACRs with FilterFFPE” in our main manuscript (2nd paragraph) by these additional results.

* I found the evaluation of the two filtering steps very interesting, and particularly the sentences on page 11 in the supplemental "This shows that the second filtering step of FilterFFPE has achieved its expected effect (improving sensitivity in case of low coverage or low SV frequency)." and on page 13 in the supplemental: "The second filtering step mainly improves sensitivity at low coverage or low SV frequency; thereby, the improvement in sensitivity applying the second filtering step is small in real data sets, while the improvement in PPV using only the first filtering step is more pronounced." The combination of those sentences prompted a number of questions:

- Does this indicate that stage 1 alone excludes more than just ACRs, but that effect is

attenuated at increased coverage?

Yes, in the new Figure S14 you can also observe this tendency: With increased coverage, more reads can cover the breakpoint of an SV event. Therefore, the likelihood of these reads to be removed as artifact chimeric reads by mistake gets smaller.

- What fraction of reads that would have been excluded by stage 1 only and retained when stage 2 is employed (if I understood correctly, theses are reads with unique breakpoints but no SRC region)? Or perhaps more useful for the reader, what are the precision and recall for excluding ACRs with the different filter configurations?

Changes: We updated results on application of no filter, one- and two-step filtration to enable the direct comparison (see Figures S20-S23 in supplementary material).

Regarding your question: Yes, those are reads with unique breakpoints but no SRC region.

- For the real data (with sufficient coverage) does looking for the SRC regions during filtering have either a neutral or actually a net negative effects on SV calling?

Changes: We updated supplementary Figure S22. It can be observed that adding the second filtration step results in slightly lower PPV and slightly higher sensitivity.

- If there are indeed a relevant fraction of ACRs without SRC regions, is there a different mechanism that creates those reads? Is it the case that the SRC region is present but can't be detected (as suggested on page 5: "However, sequencing noise in ACRs may harm the correct detection of SRC regions.") or are there other error mechanisms at work?

Currently we have not observed or had theories about any other mechanism. In addition, verification of possible mechanisms requires laboratory work, which is beyond the scope of this work.

I struggled to compare the effects of the two filter stages using Figures S12-S13, S14-S15, and S16-S17. I found myself flipping back and forth, but it is difficult to compare the values that way. Could Figures S14-S15 be integrated to show "No filter", "Stage 1 only" and "Stage 1 & 2" (i.e., three bars instead of two) to permit direct comparison? And perhaps something similar for Figures S16-S17? I recognize that doing the same for Figures S12-S13 might become unreadable. Is there another way to structure FigureS13 to more directly show the differences between the two filter configurations?

Changes: We now integrated results with no filter, with one- and two-step filtration into a single figure to enable the direct comparison (see Figures S20-S23 in supplementary material).

For Figures S18 and S19 (former Figures S12 and S13), it is difficult to merge the results in one readable figure. Therefore, we provided two additional Figures S20 and S21 to enable direct comparison. Since the second filtration step is specifically designed for low coverage or low SV frequency, we split the simulated samples into 6 samples with low coverage / low SV frequency, and 35 other samples. This allows the reader to compare the effect of one- and two-step filtration in these two contexts.

* The sections above prompted me to wonder if "stage 1" is a useful filter to apply prior to SV calling generally, not just for FFPE samples (at least with high coverage). How does stage 1 vs. stage 1 and 2 impact the sensitivity and PPV for SV calls in the FF samples? If stage 1 is an interesting filter "across the board", perhaps add a brief description of its utility to the discussion?

Powered by Editorial Manager® and ProduXion Manager® from Aries Systems Corporation

	Although we like the idea of introducing 1-step filtration with FilterFFPE as a general step in the workflow of SV calling, we do not observe much evidence that it would actually be beneficial. In Figure S23 it can be observed that FilterFFPE has only little effect on FF samples, especially for Delly and Lumpy.<br><br><br>* My understanding is that FilterFFPE is removing reads and thus my initial (and likely naive) expectation is that sensitivity would be the same or decrease - not increase. If I understood correctly, the last paragraph of the results and Figure S16 indicates that after filtering all the callers are reporting fewer SVs. And from Page 7 my understanding is that all SVs (not just PASSing SVs) are considered in the analysis. If that is correct, does that indicate the callers are identifying more/different true positive SVs in the FilterFFPE data than in all the data?<br><br>Changes: We added section 11 “Increased sensitivity after application of FilterFFPE” to our supplement. Tables S3 and S4 within this section sum up the number of true calls for each tool that are exclusively called before and after FilterFFPE’s application. In the main article, we updated the corresponding paragraph in our results section accordingly.<br><br><br>* Page 7: "Despite developing a tool for realistic simulation of FFPE samples, it can be observed that sensitivity of the three SV calling tools Manta, Delly and Lumpy differed between simulated and real data. These discrepancies were mainly due to technical differences between these data sets: our simulated samples were whole chromosome sequencing data (mimicking WGS data since it is the ideal material for SV calling) while real samples contained WES data and had a shorter read length (150 bp in simulated samples vs 90 bp in real samples)." I am curious why you did not try to generate simulated data similar to the real samples. My understanding is that SimFFPE supports both WGS and WES simulation.<br><br>Changes: We updated subsection “Simulated data sets” of our methods section. To complement the real WES data that we evaluated in our manuscript, we additionally generated simulated data that are more optimal for SV calling (mimicking WGS data with 150 bp read length, n=41).<br><br><br>Minor comments/questions:<br><br>* While the choice of Lumpy, Delly, and Manta seems very appropriate for the simulated WGS data, my understanding is that those methods are not necessarily designed for WES/targeted sequencing. I recognize though that the key comparisons here are within tools (with and without FilterFFPE) as opposed to absolute sensitivity, etc. Out of curiosity, do you think you would observe similar results for WES-specific CNV callers like XHMM, etc.?<br><br>We think that it mainly depends on the sources of evidence deployed by the caller to detect SVs: Since the majority of WES-specific CNV callers (including XHMM) only use changes in read-depth (depth of coverage) to detect CNVs, we do not expect ACRs (and thus FilterFFPE) to have much impact on the results. However, in case of WES-specific CNV callers also integrating split-read and/or paired-read evidence, FillerFFPE should have similar effect on the results.<br><br><br><br>* When simulating WES data, how is the effect of the WES capture technology modeled?<br><br>Changes: We updated the description of “Simulating ACRs with SimFFPE”, mentioning that the capture efficacy is uniformly modeled.<br><br>A more realistic simulation of the capture efficacy variation might be obtained by analyzing a large amount of WES (FFPE) data, but since it is not the focus of this work, we only used a simple simulation.

Powered by Editorial Manager® and ProduXion Manager® from Aries Systems Corporation

* Page 3: "In real data, we observed that some read pairs from adjacent ACFs both align to the same genomic locus. We found out that this is a special phenomenon arising from the enzymatic fragmentation of some adjacent ACFs…" I had trouble following the phenomenon being described in this section. Perhaps a naive question, but is this a function of the specific library preparation method, or does this occur generally in FFPE samples? How does SimFFPE model the fragmentation location?

Changes: We modified the description in our methods section, subsection “Simulating ACFs” to make it clearer.

Regarding your question: Yes, it is due to the specific library preparation method.

* I found Figure 5 confusing at first. Based on the convergence of arrows at "SV calling" I initially thought that the simulated data was used during evaluation of the real FFPE and FF samples. But if I understood correctly those are entirely separate analyses. If that is indeed correct, I would suggest having separate a "SV calling" box for the for the simulated and real data workflows.

Changes: We modified the figure accordingly.

* I find Figure 8 difficult to interpret since you have to match the dots manually based on the very small text. Is the intent for the reader to look at the shift of individual points, or to look at the shift in the overall distribution? If the latter, is there an alternate visualization of the distribution? Or perhaps overlay a cross with the means, quartiles, etc.?

Changes: Thank you for this suggestion. The shift in the overall distribution is more important. We modified the figure, removed text labels and added box plots to the sides for better visualization.

* Page 7: I would advocate for including the standard deviation along with the means (or the range of changes observed) to provide additional context for interpreting the changes in sensitivity and PPV before and after applying FilterFFPE.

Changes: Standard deviation was added to the text.

Reviewer #2:

Major comments

1. On pages 4-5, under section 'Simulating ACFs', the authors mention a higher probability of SCRs forming between adjacent chromosomal regions or within the same chromosome, based on 18 real FFPE samples. However, the Figure 2 indicates the probability of SCRs on the same chromosome as 43%, and that on different chromosomes as 57%.

Changes: We updated the description of “Simulating ACFs” in our methods section.

We see your point that the probability of SCRs on the same chromosome is – altogether – lower compared to different chromosomes. In our description in the methods we just wanted to point out that 27% (adjacent region on the same chromosome) is a relatively high number. It has to be taken into account that the length of one chromosome is much shorter than the total length of all other chromosomes, and even much shorter is the length of an adjacent region on one chromosome. Furthermore, a higher percentage can be observed for the same strand within the group of adjacent regions on the same chromosome. Within the group of regions on different chromosomes, the percentage regarding the strand is equal (50%).

Powered by Editorial Manager® and ProduXion Manager® from Aries Systems Corporation

	2. Figure 8 shows sample-wise and tool-wise differences in relationship between positive predictive values and sensitivity of SV detection, using real data. It is not clear what causes sample-to-sample differences in this relationship. Can the authors elaborate further?<br><br>Changes: We added a paragraph to our discussion (the 2nd to last paragraph) elaborating on this question.
**Additional Information:**	
**Question**	**Response**
Are you submitting this manuscript to a special series or article collection?	No
**Experimental design and statistics**<br><br>Full details of the experimental design and statistical methods used should be given in the Methods section, as detailed in our Minimum Standards Reporting Checklist. Information essential to interpreting the data presented should be made available in the figure legends.<br><br>Have you included all the information requested in your manuscript?	Yes
**Resources**<br><br>A description of all resources used, including antibodies, cell lines, animals and software tools, with enough information to allow them to be uniquely identified, should be included in the Methods section. Authors are strongly encouraged to cite Research Resource Identifiers (RRIDs) for antibodies, model organisms and tools, where possible.<br><br>Have you included the information requested as detailed in our Minimum Standards Reporting Checklist?	Yes
**Availability of data and materials**<br><br>All datasets and code on which the conclusions of the paper rely must be either included in your submission or	Yes

deposited in publicly available repositories (where available and ethically appropriate), referencing such data using a unique identifier in the references and in the "Availability of Data and Materials" section of your manuscript.<br><br>Have you have met the above requirement as detailed in our Minimum Standards Reporting Checklist?	

Powered by Editorial Manager® and ProduXion Manager® from Aries Systems Corporation

Manuscript
Click here to access/download;Manuscript;Revised_Main_SimFFPE_and_Fi

OXFORD
(GIGA)^n SCIENCE

GigaScience, 2021, 1–10

doi: xx.xxxx/xxxx
Manuscript in Preparation
Technical Note

TECHNICAL NOTE

SimFFPE and FilterFFPE: improving structural variant calling in FFPE samples

Lanying Wei[1,*], Martin Dugas[1,2] and Sarah Sandmann[1]

[1]Institute of Medical Informatics, University of Münster, Münster, 48149, Germany and [2]Institute of Medical Informatics, Heidelberg University Hospital, Heidelberg, 69120, Germany

*lanying.wei@uni-muenster.de

ORCIDs:
Lanying Wei, 0000-0002-4281-8017; Martin Dugas, 0000-0001-9740-0788; Sarah Sandmann, 0000-0002-5011-0641.

Abstract

Background: Artifact chimeric reads are enriched in next-generation sequencing data generated from formalin-fixed paraffin-embedded (FFPE) samples. Previous work indicated that these reads are characterized by erroneous split-read support that is interpreted as evidence of structural variants. Thus, a large number of false positive structural variants is detected. To our knowledge, no tool is currently available to specifically call or filter structural variants in FFPE samples. To overcome this current gap, we developed two R packages: SimFFPE and FilterFFPE. **Results:** SimFFPE is a read simulator, specifically designed for next-generation sequencing data from FFPE samples. A mixture of characteristic artifact chimeric reads as well as normal reads is generated. FilterFFPE is a filtration algorithm, removing artifact chimeric reads from sequencing data, while keeping real chimeric reads. To evaluate the performance of FilterFFPE, we performed structural variant calling with three common tools (Delly, Lumpy and Manta) with and without prior filtration with FilterFFPE. After applying FilterFFPE, the average positive predictive value improved from 0.27 to 0.48 in simulated samples, and from 0.11 to 0.27 in real samples, while sensitivity remained basically unchanged or even slightly increased. **Conclusions:** FilterFFPE improves the performance of SV calling in FFPE samples. It was validated by analysis of simulated and real data.

Key words: FFPE; Next-generation sequencing; Artifact removal; Structural variant calling

Background

For decades, formalin fixation and paraffin embedding (FFPE) has been widely used to prepare and preserve biopsy specimens [1]. FFPE tissues preserve morphological and cellular details and provide a method for long-term storage at room temperature. These advantages make FFPE tissues the most common sources of archived clinical material: it is estimated that more than 400 million FFPE samples are currently available, many of which have corresponding clinical records, including diagnoses, treatment options, and drug responses [1]. Furthermore, rare tumors are most often stored as FFPE samples [2]. Therefore, FFPE samples provide a common and valuable source for medical research.

Next-generation sequencing (NGS) plays an important role in medical research. It allows us to investigate entire genomes, uncover the molecular characteristics of diseases, and provide insights into therapies. However, formalin-fixation can result in fragmented, degraded, protein cross-linked DNA, introducing false positive results to NGS data analysis [3]. The interpretation of NGS data strongly relies on bioinformatics tools; therefore, to analyze FFPE samples, these tools need to be optimized to minimize the number of false positive or false negative results.

NGS can be used to detect genomic variants of different scales: single nucleotide variants, short insertions/deletions and structural variants (SVs), including copy number variants (CNVs). So far, studies on FFPE-specific artifacts have been focusing on false positive SNVs and only few on CNVs [3]. When performing variant calling on FFPE samples, we observed a large number of false positive SVs. However, to our knowledge no study has yet considered filtration of these calls. Artifact chimeric reads (ACRs) are known to be en-

Compiled on: July 19, 2021.
Draft manuscript prepared by the author.

riched in FFPE samples [4] and are likely leading to false positive SV calls. It is hypothesized that these ACRs are derived from the binding of single-stranded DNA (ss-DNA) fragments [4]. The proportion of ss-DNA is much higher in FFPE samples than in fresh frozen (FF) samples, because double-stranded DNA (ds-DNA) is denatured due to high temperature used in the deparaffinization and reverse cross-linking steps for DNA extraction from FFPE samples [4, 5]. These ss-DNAs may randomly self-assemble if short reverse complementary (SRC) regions exist. During the end-repair step of library construction, T4 DNA polymerase removes the 3' overhangs and fills in the 5' overhangs of the binding product [4], thereby producing artifact chimeric ds-DNA, which eventually leads to false positive SV calls (see illustration in Supplementary Material, Figure S1).

To evaluate and improve the performance of SV calling algorithms in FFPE samples, ground truth data is needed. However, publicly available real-world FFPE data sets with matched FF samples are scarce. Furthermore, to our knowledge, no experimental validation of SV candidates is available for these data sets. Therefore, we simulated data with known biological truth, and performed expert-based validation of SV calls for two real data sets with FFPE and matched FF samples.

Aiming at improving SV calling performance in FFPE samples, we defined the following research objectives: 1. To develop an NGS read simulator that can specifically simulate ACRs in FFPE samples. The simulated reads should be as realistic as possible. 2. To develop a tool that successfully removes ACRs while keeping non-artifact chimeric reads resulting from real SVs. 3. To benchmark existing SV callers by using simulated as well as real NGS data sets resulting from FFPE samples, and to evaluate the effect of ACR removal on SV calling.

Methods

Data sets

Real data sets

Two real world data sets were analyzed in this study. Both contain whole-exome sequencing (WES) data of FFPE and matched FF samples publicly available at the European Nucleotide Archive (Supplementary Material, Table S1). The first data set contains 13 FFPE breast tumor samples and 13 corresponding FF samples (Accession number: SRP044740). The second data set contains 5 FFPE samples with unspecified tumor type and 4 corresponding FF samples (Accession number: PRJNA301548; note: two FFPE samples belong to the same patient).

Simulated data sets

The real data available to us are all WES data, however, the ideal data for SV calling is whole genome sequencing (WGS) data with sufficient read length. Therefore, to complement the available real data, we generated simulated data that are more optimal for SV calling (mimicking WGS data with 150 bp read length). To generate simulated data sets, we first simulated 400 non-overlapping SVs with varying lengths (1 kb to 10 kb; 100 duplications, 100 deletions, 100 inversions and 100 translocations) on chromosome 12 of genome assembly hg19 using RSVsim [6]. Next, we applied SimFFPE (algorithm described in section "Simulating ACRs with SimFFPE") to the mutated as well as the original chr12 sequence to generate simulated FFPE NGS reads that cover the whole chromosome. Notably, the 100 translocations were simulated as large insertions of random segments from other chromosomes. To evaluate the effect of FilterFFPE (algorithm described in section "Filtering ACRs with FilterFFPE") on SV calling, we generated three simulated data sets (see Table 1). Altogether, 41 samples were simulated.

Table 1. Characteristics of the simulated data sets. Abbreviations: SV - structural variant; ACF - artifact chimeric fragment.

Name	n	Coverage	SV frequency	Proportion of ACFs
Sim1	10	10-100x	50%	10%
Sim2	10	50x	10-100%	10%
Sim3	21	50x	50%	0-20%

Simulating ACRs with SimFFPE

The general workflow of SimFFPE is shown in Figure 1.

The whole simulation can be split into two parts - the simulation of normal fragments and the simulation of artifact chimeric fragments (ACFs). While normal fragments are simulated directly from the reference genome, the simulation of ACFs is much more complex. Details of ACF simulation are described in the subsection "Simulating ACFs". We observed normally distributed fragment lengths in real data; therefore, we used a normal distribution to simulate fragment lengths. This observation is in line with several other publications on NGS simulators, e.g. [7, 8].

Simulations for WGS as well as WES and targeted sequencing data are supported. For WES and targeted data, we uniformly model the capture efficacy. Simulated read sequences are generated from one end (single-end sequencing) or both ends of the fragments (paired-end sequencing). We refer to the reads generated from ACFs as ACRs. It should be noted that well-known errors in NGS data, such as base substitutions and indels, are not the focus of this work; therefore, SimFFPE only performs simple random error simulations.

Phred quality scores are correlated with base position in the reads [8]. Accordingly, SimFFPE estimates positional Phred score profiles from real NGS data for simulations. We provide two exemplary positional Phred score profiles for read lengths of 100 and 150 bp.

Simulating ACFs

To simulate ACFs, the essential task is to find genome sequence pairs with SRC regions and combine them to form double-stranded fragments. A graphic representation of this process is available in Supplementary Material (Figure S1).

To locate candidate SRC pairs for binding, we randomly select short (on average 6 bp) genome sequences (referred to as "seed sequences") and find their reverse complementary sequences (referred to as "target sequences"). The obvious match - target sequences at the same genomic location on the reverse strand - are excluded.

For a given seed sequence, there can be millions of candidate target sequences. If one target sequence was randomly selected, this could result in simulated data largely deviating from real data. To simulate data as realistic as possible, an elaborate set of characteristics is considered when simulating SRC pairs. Among others, these characteristics include SRC region length distribution, location (whether from the same chromosome, and if yes, whether from adjacent chromosomal regions), distance and strand (Figure 2). All default distributions and proportions of SimFFPE are based on the characteristics of the 18 real FFPE samples from the two public data sets mentioned above (Supplementary Material, section 3). Based on the SRC region length distribution in these samples, we decided to use a log-normal distribution (μ=1.8, σ=0.55) to approximate the true distribution. More information about the relevant parameters can be found in the vignettes and reference manual of the SimFFPE package (`http://www.bioconductor.org/packages/release/bioc/html/SimFFPE.html`).

Since only one target sequence of a seed is finally selected, computational costs are greatly reduced by SimFFPE identifying target sequences of a seed only within a small region. More specifically, we partition the genome into small windows (5 kb). Target sequences are searched in a random window or within the same window of

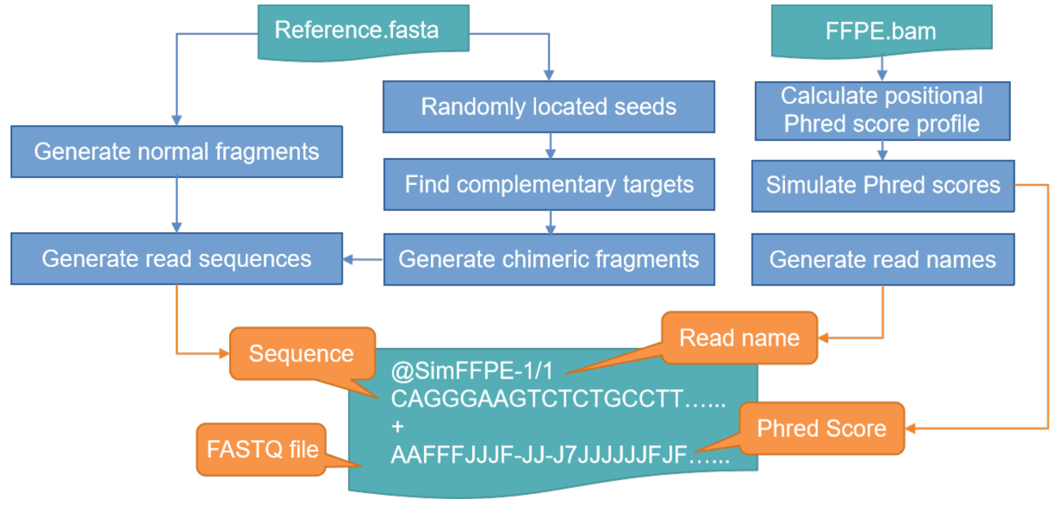

Figure 1. Workflow of SimFFPE. The algorithm generates normal- and artifact chimeric fragments, and simulates read sequences from these fragments. Phred scores are simulated based on read position. A FASTQ file is generated as output.

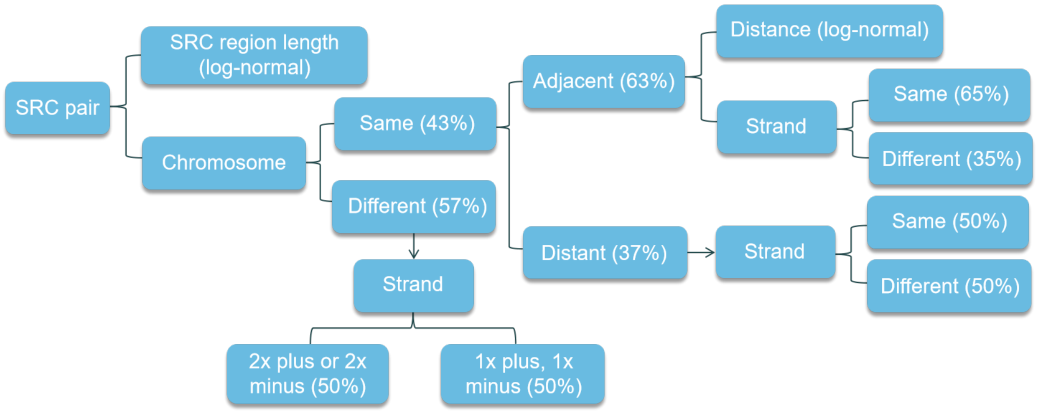

Figure 2. Aspects that are considered when simulating SRC pairs. The proportions and distribution models shown in parentheses are SimFFPE's default settings, which are determined on the basis of 18 real FFPE samples from two public data sets. Abbreviations: SRC - short reverse complementary.

the seed. The resulting SRC pairs and ACFs are called between-window SRC pairs and distant ACFs, and within-window SRC pairs and adjacent ACFs, respectively.

The reason to differentiate between these two cases is as follows: we observed that in real FFPE samples, around 27% (43% on the same chromosome × 63 % from adjacent chromosomal regions) of ACFs are derived from the binding of adjacent (within 5 kb) SRC pairs (Supplementary Material, Figures S5 and S6). Due to the small window size and the sheer human genome size (over 3 billion bp), such a relatively high proportion indicates a high chance of binding between two adjacent ss-DNAs. It appears reasonable to assume that two ss-DNAs originating from adjacent genomic regions are, on average, physically closer to each other and thus have a higher chance of binding. Accordingly, we divide the ACF simulation into two parts: the adjacent ACF simulation and the distant ACF simulation. For both, several demands have to be met.

The most important considerations for adjacent ACF simulation are as follows (details in Supplementary Material, Figures S7-S9): 1) In real data, we observed a relatively high proportion of adjacent ACFs resulting from genomically close SRC pairs (50-200 bp). Analyzing the distribution of the distance between the combined SRC pair in real data, we decided to choose a log-normal distribution (μ=4.7, σ=0.35) for simulation, as this closely resembles real data. 2) One SRC pair may originate from different strands of DNA or from the same strand. The probabilities for these two cases are not equal. We observed a higher proportion of same-stranded (65%) versus different-stranded (35%) SRC pairs in adjacent ACFs in real data. A corresponding parameter (sameStrandProp; default=0.65) was set. It seems possible that long ss-DNA molecule might form hairpin structure and generate chimeric ds-DNA. This might explain the higher proportion of same-stranded SRC pairs in adjacent ACFs. 3) In some real samples, we observed that some read pairs from adjacent ACFs both align to the same genomic locus (Supplementary Material, Figure S9). We found out that this occurs when enzymatic fragmentation is used in library preparation. Enzymes are able to recognize and cut at specific sites of the genome. As shown in Figure 3, an adjacent ACF can be a repeat or an inverted repeat of a DNA sequence. Thus, enzymatic fragmentation leads to both ends of the ACF being cut at the same genomic locus. If the ACF is an inverted repeat and is enzymatically fragmented (with both sides end at the same genomic position), then the read pair are sequenced from the same starting point, and proceed with the same sequence (until the end of the repeat unit). As a result, this read pair is mapped to the same genomic locus. Accordingly, SimFFPE supports simulation of enzymatic fragmented adjacent ACFs. When enzymatic fragmentation is simulated, SimFFPE cuts the adjacent ACF (if it is a repeat) at a random site in one end, and cuts the other end at the same genomic locus of the repeated sequence. For simplicity, we did not model the specific cutting sites of enzymes, but we ensured that the fragment length distribution was still well simulated.

For distant ACF simulation, several additional aspects have to be taken into account: 1) Analysis of the real data sets indicates that strand usage for the formation of distant ACFs is almost equal. 2) In real data sets, we observed a common feature across the whole

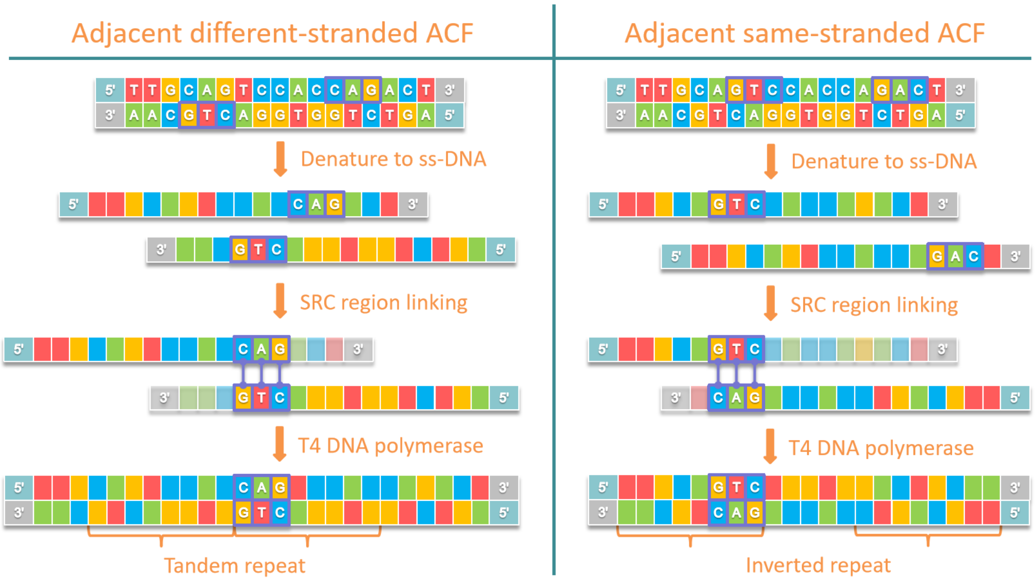

Figure 3. Examples of forming a repeat and an inverted repeat by adjacent ss-DNA combination. Abbreviations: ACF - artifact chimeric fragment.

Table 2. Differences in simulating adjacent and distant ACFs. Abbreviations: SRC - short reverse complementary; ACF - artifact chimeric fragment.

	Adjacent ACF	Distant ACF
SRC pair	Within-window	Between-window
Strand usage of the SRC pair	Unequal	Equal
Distance between the SRC pair	Log-normal	Random
Enzymatic fragmentation simulation	Applicable	/
“Spike” simulation	/	Applicable

genome: within some small genomic regions (1-2 kb), there were more ACRs originating from distant ACFs compared to other regions. These are referred to as “spikes” (Supplementary Material, Figure S9). To simulate these “spikes”, we use a beta distribution (α=β=0.5) to model the amount of distant ACRs in each small region. Thus, the simulation enriches distant ACRs in some of these small regions.

A summary of the differences in simulating adjacent and distant ACFs is shown in Table 2.

Filtering ACRs with FilterFFPE

The workflow of FilterFFPE is shown in Figure 4. To filter ACRs while preserving informative chimeric reads resulting from true SVs, it is important to identify features that can help to distinguish between these two types of chimeric reads. As long as sequencing depth and SV frequency are not too low, more than one or even dozens of chimeric fragments may cover the same breakpoints of the SV event (Figure 4b). However, since the binding of two ss-DNAs is a rather random event, there is little chance that the breakpoint of an ACF is also present in other ACFs or in chimeric reads from real SV events (note that for pair-ended sequencing, the read pair from an ACF can share the same breakpoints, but both reads belong to the same fragment). Therefore, an apparent filtering strategy is to evaluate the number of reads sharing the same breakpoint: if n or less chimeric reads (default: n=2 for paired-end sequencing) share the same breakpoints, these reads are recognized as potential ACRs. Notably, chimeric reads covering true SVs may also be identified as potential ACRs at low sequencing depths or low SV frequencies. Therefore, we need an additional feature to validate these potential ACRs. This feature is the presence of an SRC region. According to the ACF formation mechanism, only the presence of an SRC region causes two ss-DNAs to bind and form an ACF. Therefore, FilterFFPE analyzes if an SRC region is present next to the breakpoint of an ACR candidate.

The detection of an SRC region is based on the main characteristics of ACRs. ACRs contain two genome segments - one from the seed sequence and one from the complementary target. Thus, there exist (at least) two alignments, both containing soft-clipped bases. In an ACR, towards the end of the mapped sequences, a short region should be mapped in both alignments. This region can be identified as the SRC region that links two ss-DNAs forming the ACF. First, FilterFFPE identifies potential ACRs. Second, the presence and lengths of SRC regions within these ACR candidates is analyzed. Only reads with plausible SRC regions (SRC region length $\geq$ m, with default m=1 to remove as many ACRs as possible) are removed by FilterFFPE. This step helps not to exclude real chimeric reads resulting from low coverage regions or low frequency SVs by mistake, i.e. preserving sensitivity. However, sequencing noise in ACRs may harm the correct detection of SRC regions. Thus, it is possible that some ACRs are falsely categorized as real chimeric reads, i.e. positive predictive value (PPV) is decreased. Therefore, this second filtration step is optional.

After determining the reads to be excluded, FilterFFPE generates a filtered and indexed BAM file, as well as a text file containing the names of the excluded reads.

Performance evaluation

The steps taken to evaluate SV calling performance in real and simulated FFPE samples with and without application of FilterFFPE are shown in Figure 5 (see Supplementary Material, section 4 for information on sequence alignment, duplicate removal, downsampling, etc.). Three SV callers, Delly (v0.7.9) [9], Lumpy (v0.3.1) [10] and Manta (v1.6.0) [11] were used for SV calling. These tools have performed best in recent benchmarking studies [12, 13, 14].

For real data sets, each pair of matched FFPE and FF samples was down-sampled to the same size. Furthermore, only reads within exonic regions with sufficient coverage were used for SV detection (exonic regions with average coverage $\geq$ 30x in both samples of the

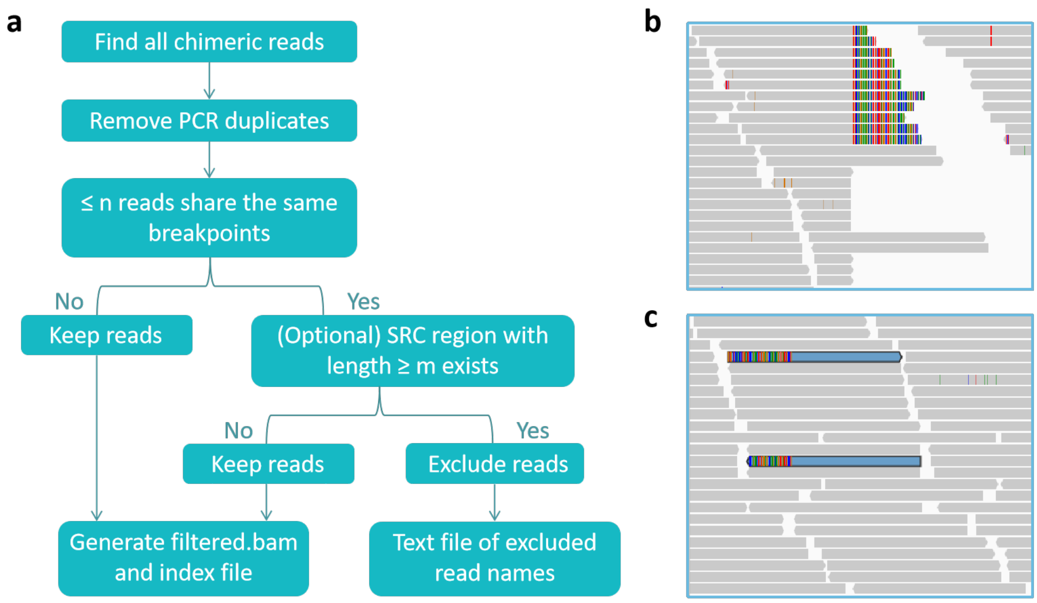

Figure 4. Filtration with FilterFFPE. (a) The workflow of FilterFFPE to filter out ACRs. Values of n and m are user-definable. (b) Breakpoint of a true deletion. (c) Breakpoint of an ACR pair. Abbreviations: PCR - polymerase chain reaction; SRC - short reverse complementary; ACR - artifact chimeric read.

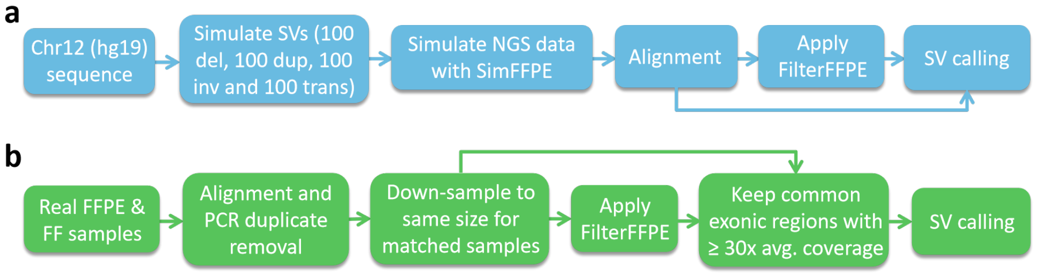

Figure 5. Performance evaluation of SV callers Delly, Lumpy and Manta with and without application of FilterFFPE considering real and simulated FFPE samples. Steps performed in case of (a) simulated data and (b) real data are visualized. Abbreviations: dup - duplications; del - deletions; inv - inversions; trans - translocations; FFPE - formalin-fixed paraffin-embedded; FF - fresh frozen; SV - structural variant.

pair). PPV, sensitivity and F1-score were used to evaluate each tools' SV calling performance with and without application of FilterFFPE.

Different SV callers can detect the same breakpoint with minor shifts in the genomic location. To determine if an SV call indicates a true positive SV and if it is shared between two samples, a maximum shift of $\pm$ 5 bp is allowed to identify consistent breakpoints. This threshold was determined based on a previous evaluation on different SV callers' breakpoint resolution by Gong et al. [13].

Since data on experimental validation of SV candidates in real samples was not available, we performed expert-based validation by two independent experts in the field of SV detection (see Supplementary Material, section 5 for characteristics used to determine true positives). To facilitate manual inspection, we divided SV calls in FFPE samples (before and after FilterFFPE's application) into four categories: 1. SV calls with high probability of being true positives (1,041 SV calls). These are shared SV calls with matched FF samples (without application of FilterFFPE in FF samples). 2. SV calls with reduced probability of being true positives (2,282 SV calls). These are non-shared SV calls with reliable support (shared with at least one non-matched FF sample, or having in total $\geq$ 10 reads of split- and/or paired-read support). The criteria for reliable support was determined on the basis of prior manual inspection of 500 randomly selected non-shared SV calls. 3. SV calls with low probability of being true positives (1,952 SV calls). These are non-shared SV calls without reliable support (do not match a call in any FF sample and have less than 10 supporting reads), but showed characteristics that we identified in categories one and two as being typical for true positive variants (matching a call that is labeled as true positive in any other FFPE sample, or overlapping with a gene that is characterized by a high number of SV calls). 4. SV calls with high probability of being false positives (the rest 44,877 SV calls).

We manually inspected all 5,275 SV calls in the first to third category. The SV calls in the fourth category were automatically labeled as false positives as they lack characteristics of potentially true variants. To ensure that this automatic classification was reliable, we randomly selected 2,000/44,877 calls and performed manual inspection. In total, 1,996 of these 2,000 calls are false positives, the rest 4 are ambiguous (and are remained after application of FilterFFPE, for details see Supplementary Material, section 5). We therefore consider it plausible to automatically label the whole category as false positives. In total, we labeled 1,506 SV calls as true positive and 47,026 as false positives. Besides, 1,620 SV calls could not be classified clearly and were thus excluded from further evaluation. The number of SV calls with information on initial category and final judgment is shown in Supplementary Material (Table S2).

Results

Simulating realistic FFPE data using SimFFPE

A comparison of SimFFPE to existing NGS data simulators is shown in Table 3. The other tools mainly serve to simulate read-level sequencing noise. In contrast, SimFFPE additionally simulates ACFs

Table 3. Comparison of SimFFPE to existing NGS data simulators. Abbreviations: OS - operating system; L - Linux; M - MacOS; W - Windows; WGS - whole genome sequencing; WES - whole exome sequencing; SE - single-end sequencing; PE - paired-end sequencing; ACR - artifact chimeric read; SeqE - sequencing error.

Name	Installation	Language	OS	Sequencing type	Read type	Noise type
SimFFPE	Bioconductor, Bioconda	R	L, M, W	WGS, WES	SE, PE	ACR, SeqE
ART [7]	Bioconda, manual	C++, Perl	L, M, W	WGS	SE, PE	SeqE
BEAR [15]	Manual	Python, Perl	L	WGS	SE, PE	SeqE
FASTQSim [16]	Manual	Bash, Python	L	WGS	SE	SeqE
GemSim [17]	Manual	Python	L, M, W	WGS	SE, PE	SeqE
Grinder [18]	Manual	Perl	L, M, W	WGS	SE, PE	SeqE
InSilicoSeq [19]	Bioconda, manual	Python	L, M, W	WGS	PE	SeqE
NeSSM [20]	Manual	Python	L	WGS	SE, PE	SeqE
pIRS [21]	Bioconda, manual	C++, Perl	L	WGS	PE	SeqE
SimuSCoP [8]	Manual	C++	L	WGS, WES	SE, PE	SeqE
SInC [22]	Manual	C++	L	WGS	PE	SeqE

that are characteristic of FFPE samples. These ACFs are fragment-level noise that can lead to false-positive SV calls.

Figure 6 shows exemplary aligned reads generated by SimFFPE. For comparison, data from a real sample (FFPE and matching FF) is displayed. SimFFPE generates ACRs that closely resemble those highly noisy reads in real FFPE samples. In contrast, existing simulators such as ART [7], only produce normal reads similar to those in FF samples (Supplementary Material, Figure S9). We further compared the proportion of abnormally paired reads in real and simulated samples (Supplementary Material, section 7). The proportion of abnormally paired reads is higher in real FFPE samples than in FF samples. The distribution of this proportion in simulated data set Sim3 (with varying artifact levels) is very close to that of real FFPE samples.

Filtering FFPE-specific ACRs with FilterFFPE

On all real and simulated samples, we performed filtration with FilterFFPE (using default setting with 2-step filtration; for results on filtration with FilterFFPE applying the first step only see Supplementary Material, section 8-10).

For each simulated sample, excluded reads could be divided into ACRs and normal reads (tagged by SimFFPE when generating the data) and counted separately (Supplementary Material, Figure S13). As a result, in 40 out of 41 simulated samples (as 1 sample without any ACFs was simulated), 99.73% to 100.00% of the removed reads were ACRs (average: 99.96%, see Supplementary Material, Figure S14, results with two-step filtration). These excluded ACRs account for 97.72% to 97.94% (average: 97.82%) of all chimeric reads derived from ACFs (Supplementary Material, Figure S16). Reads obtained from ACFs can also be non-chimeric (without supplementary alignment): these include reads that do not cover breakpoints, or cover only a few bases of one of the two original sequences (see Supplementary Material, Figure S15 for illustration). These non-chimeric reads do not lead to artifact split-read support, and are thus not removed by FilterFFPE. Therefore, the percentage of excluded ACRs based on all reads from ACFs is 65.63% on average (Supplementary Material, Figure S17).

In real data sets, we applied FilterFFPE to both FFPE and matched FF samples. The percentage of filtered reads ranged from 0.33% to 9.2% in FFPE samples (median: 2.5%). In contrast, only 0.015% to 0.33% (median: 0.10%) were filtered in FF samples. These results match our previous observation that ACRs are enriched in FFPE samples compared to FF samples. It should be noted that FF samples are expected to contain some ACRs, as any heating step during sequencing can result in DNA denaturation and thus ACR generation. Nevertheless, since the percentage of ACRs in FF samples is low, the effect of these ACRs on SV calling is usually neglectable (Supplementary Material, Figure S23).

Evaluation of SV calling with and without previous filtration with FilterFFPE

Figure 7 shows the performance of the 3 SV calling tools Delly, Lumpy and Manta on the 3 simulated data sets with and without previous application of FilterFFPE. Results show that FilterFFPE substantially improves PPV of SV calling, considering a diverse set of scenarios, while only affecting sensitivity in few exceptional cases. Thus, an overall improvement in F1-score is observed.

As simulated coverage or ACF proportion increases, the number of ACRs increases; therefore, we expected and also observe an increasing number of false positive SV calls and decreasing PPV. SV frequency has no effect on the number of ACRs and thus, we did not expect any effect on the number of false positive SV calls. It can be observed that both Manta and Delly are characterized by stable PPV at different SV frequencies. Interestingly, Lumpy shows a decrease in PPV with increasing SV frequency. Detailed evaluation of the SV calling results revealed that Lumpy generated several SV candidates for real SVs with different breakpoints. Some of these SV candidates were recognized as false positives, as the detected breakpoints were not close enough to the real ones ($\pm$ 5 bp).

After removing ACRs with FilterFFPE, PPVs of all three tools increase in all our simulated datasets: Manta shows the largest increase (on average from 0.06$\pm$0.15 (mean$\pm$standard deviation) to 0.45$\pm$0.21), followed by Delly (0.10$\pm$0.18 to 0.29$\pm$0.22) and Lumpy (0.65$\pm$0.13 to 0.71$\pm$0.12).

Sensitivity of the three tools is stable across all simulated data sets, except for low coverage ($\leq$30x) or low SV frequencies ($\leq$30%). In these extreme cases, it is difficult to distinguish between real chimeric reads and ACRs. Therefore, application of FilterFFPE slightly reduces sensitivity (on average from 0.83$\pm$0.13 to 0.78$\pm$0.17; 6 samples). For all other samples, sensitivity even increases marginally after using FilterFFPE (on average from 0.94$\pm$0.05 to 0.95$\pm$0.05). Compared to the other tools, Delly is characterized by lowest sensitivity - before and after filtration with FilterFFPE. This is due to the fact that Delly did not detect translocations with precise genomic location: 61 out of 100 simulated translocations could not be detected accurately by Delly (often with a deviation of 30-300 bp at the breakpoint).

It should be mentioned that these results are based on all reported SV calls. In addition, every tool has diverse internal categories to characterize SV calls of different qualities, including *precise* vs *imprecise* calls (whether breakpoints can be precisely located) and/or *pass* vs *non-pass* calls (whether certain quality conditions are met). Interestingly, with the combined use of these categories and FilterFFPE, the best performance is observed in case of FilterFFPE+Delly, considering only *precise* calls. Delly's *precise* calls have an average F1-score of 0.71$\pm$0.14 across the three simulated data sets, and reach 0.91$\pm$0.06 with FilterFFPE. More details can be found in Supplementary Material (Figures S18-S21).

Figure 8 shows the influence of FilterFFPE on SV calling in real FFPE samples. Similar to the results in simulated data sets, applica-

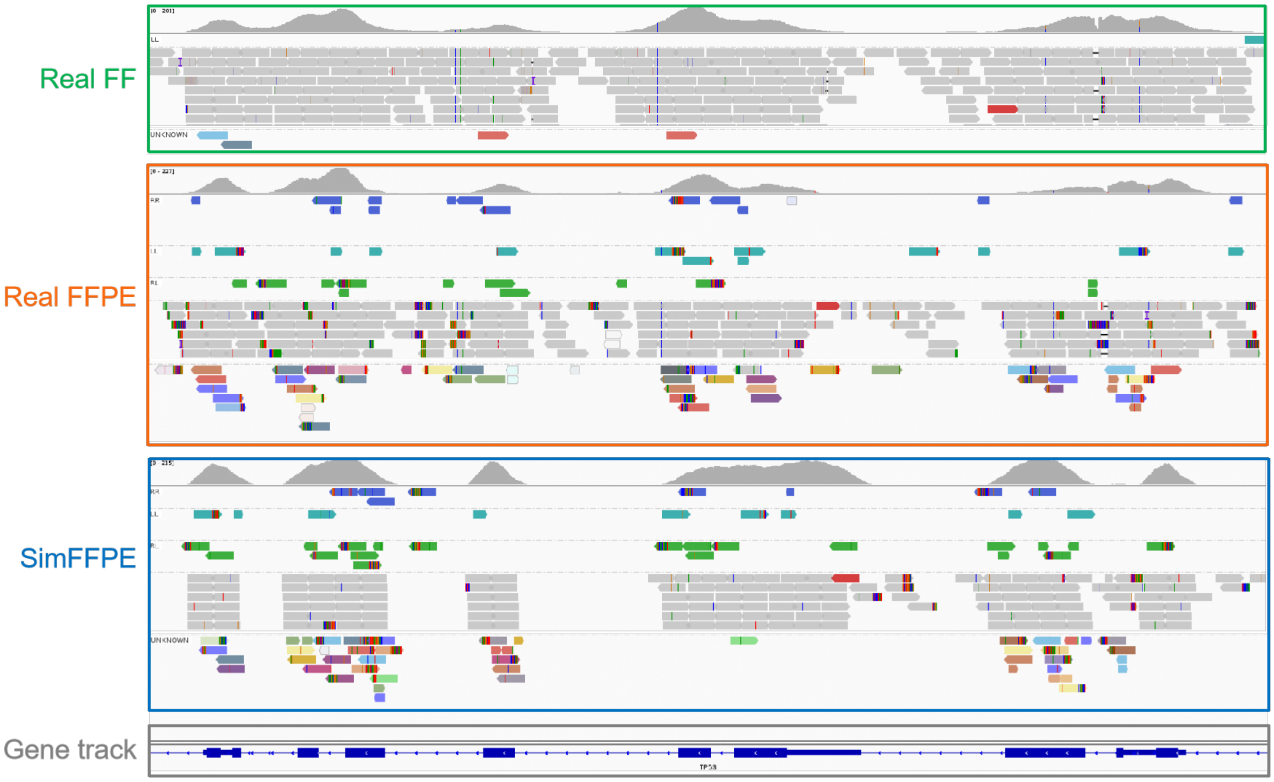

Figure 6. Exemplary alignment of reads simulated by SimFFPE in comparison to real reads in matching FF and FFPE samples. Soft-clipped bases are shown. Alignments are grouped by pair orientation. Pair orientation is presented in terms of read-strand: left (L) versus right (R), and first read versus second read of a pair. The color (not gray) of the alignment indicates an abnormal pair orientation, or a different chromosome that the paired read mapped to. Alignments with normal pair orientation are colored in grey. Abbreviations: FF - fresh frozen; FFPE - formalin-fixed paraffin-embedded.

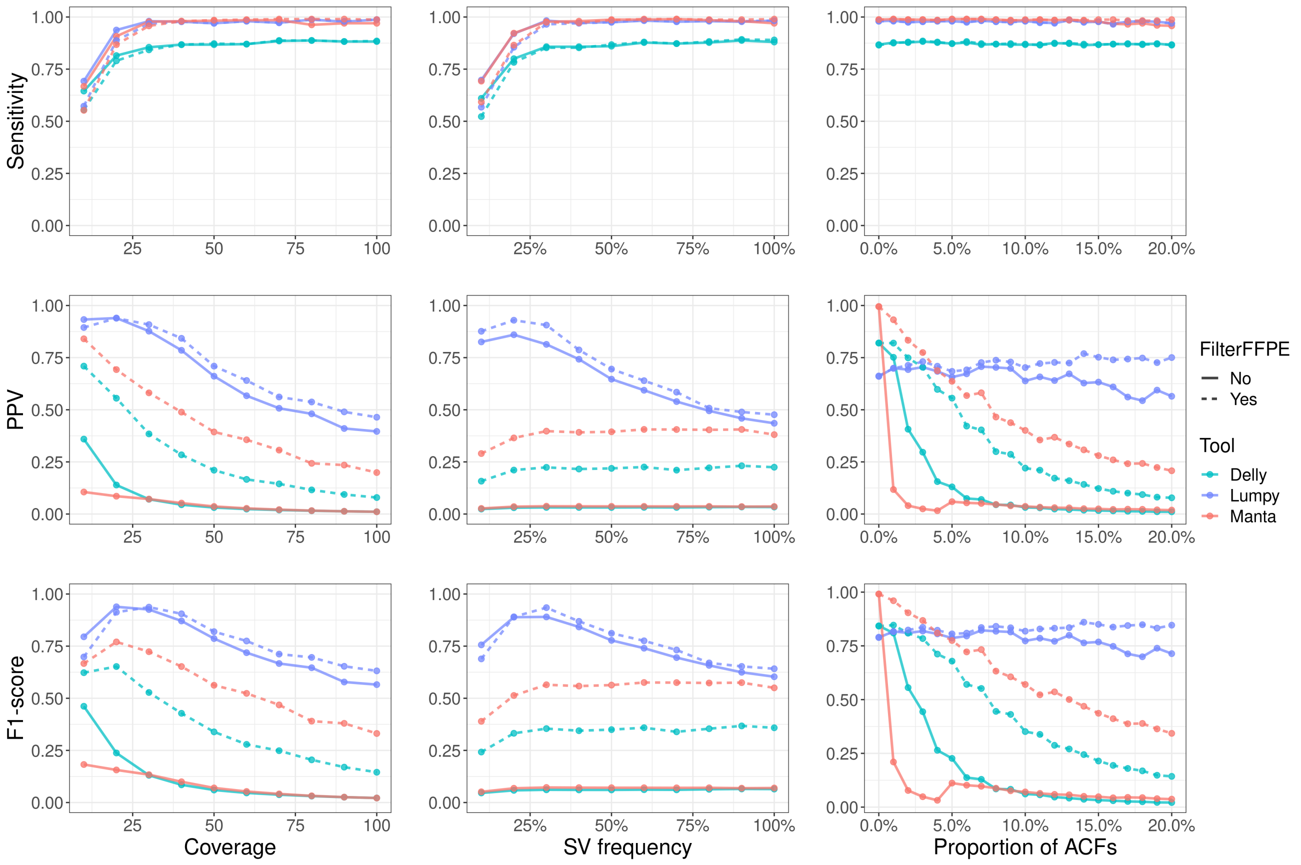

Figure 7. FilterFFPE increases PPV of SV calling in simulated samples, while keeping sensitivity unchanged. Lumpy performs best – with and without application of FilterFFPE. Abbreviations: PPV - positive predictive value.

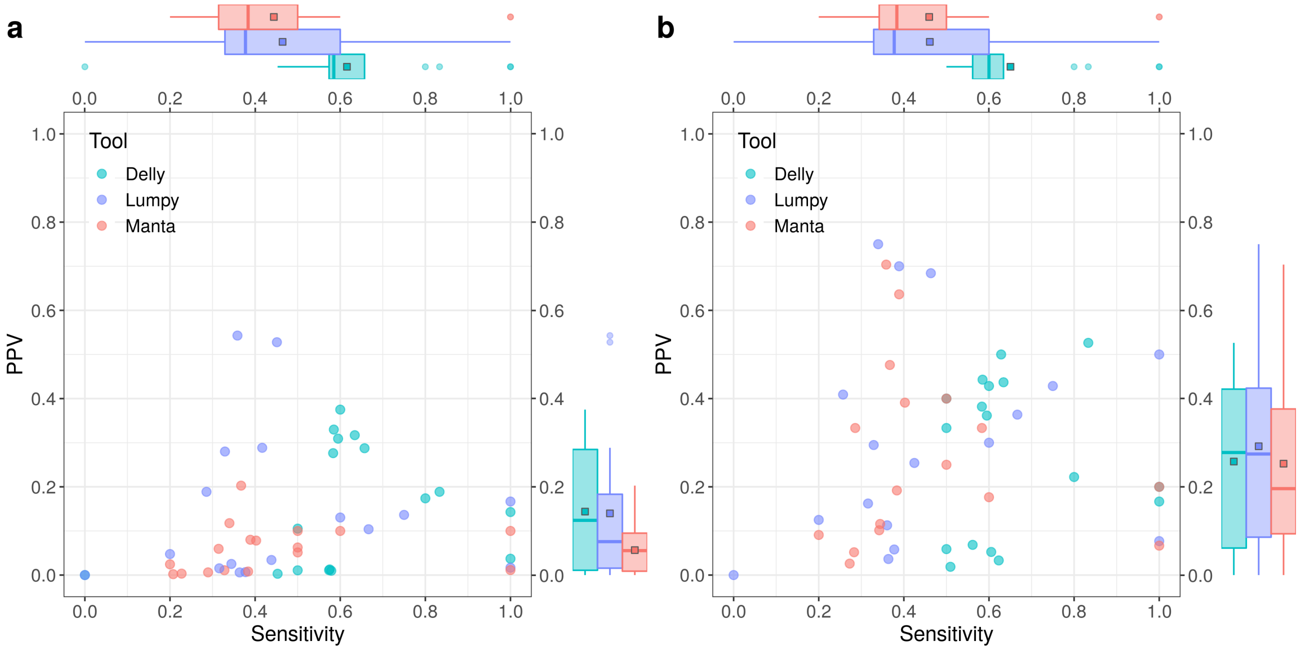

Figure 8. FilterFFPE considerably increases PPV of SV calling in real FFPE samples, while slightly improving sensitivity. PPV and sensitivity of SV calling (a) before and (b) after application of FilterFFPE are shown. Each dot represents one FFPE sample. Square in the box plot indicates mean value. Altogether, Lumpy is characterized by highest PPV and Delly by highest sensitivity. Abbreviations: PPV - positive predictive value.

tion of FilterFFPE leads to a considerable improvement in PPV and a minor improvement in sensitivity: filtration with FilterFFPE increases mean PPV in case of Delly from 0.14±0.14 to 0.26±0.19, from 0.06±0.05 to 0.25±0.21 for Manta, and from 0.14±0.17 to 0.29±0.25 for Lumpy; mean sensitivity increases from 0.62±0.23 to 0.65±0.16 for Delly, from 0.44±0.24 to 0.46±0.23 for Manta, and remains 0.46 for Lumpy. For Delly and Manta, more true positives were exclusively detected after application of FilterFFPE (Supplementary Material, Tables S3 and S4), thus resulting in increased sensitivity. Considering the tools' internal categories, the best overall performance can be observed - just like in case of simulated data - for FilterFFPE+Delly, considering only *precise* calls. Delly's *precise* calls have an average F1-score of 0.45±0.28 in real FFPE samples, and reach 0.58±0.24 with FilterFFPE. More details can be found in Supplementary Material (Figure S22).

To further validate performance of FilterFFPE, we also calculated the number of reported SV calls in FF samples before and after FilterFFPE's application (Supplementary Material, Figure S23). Over all 18 real FFPE samples, FilterFFPE reduces the number of reported SV calls by 44% (Delly), 76% (Manta) and 61% (Lumpy). In comparison, the number is reduced by only 0.3% (Delly), 5% (Manta), and increased by only 0.2% (Lumpy) in matched FF samples.

Discussion

In this paper, we introduce two R packages for improved handling of sequencing data generated from FFPE samples: SimFFPE and FilterFFPE. SimFFPE is a novel tool simulating realistic sequencing data from FFPE samples. Simulated data with known biological truth is the prerequisite for e.g. optimization of variant calling pipelines. Based on the output of SimFFPE we developed and tested a new filtration algorithm for SV calling: FilterFFPE. Results on both simulated and real data show that our filtration algorithm is able to improve PPV without compromising sensitivity of three established SV calling algorithms.

Despite developing a tool for realistic simulation of FFPE samples, it can be observed that sensitivity of the three SV calling tools Manta, Delly and Lumpy differed between simulated and real data. These discrepancies were mainly due to technical differences between these data sets: our simulated samples were whole chromosome sequencing data (mimicking WGS data since it is the ideal material for SV calling) while real samples contained WES data and had a shorter read length (150 bp in simulated samples vs 90 bp in real samples).

Sensitivity of Lumpy and Manta was much lower for real data compared to simulated data. Lumpy utilizes not only read-pair and split-read support, but also read-depth support to identify SV candidates. However, regional coverage fluctuates heavily in WES data. Thus, it can harm read-depth support detection in Lumpy and lead to lower sensitivity. The reduced sensitivity of Manta is likely due to inaccurately detected SV positions. The accuracy of Manta's local assembly might have been affected by the shorter read length of the real data. Delly showed lowest sensitivity in simulated data sets but featured highest in real data. It could be observed that Delly's imprecise positioning of translocations leads to false negative calls. In our simulated data, 25% (100/400) of all SVs were translocations, but only 2% (7/296) in real data.

Since the purpose of SimFFPE and the type of its simulated noise are different from those of existing simulators, it is difficult to compare SimFFPE with other simulation tools. However, exemplary comparison of simulated and real data in the IGV shows that reads generated by SimFFPE resemble real FFPE samples, while reads generated by other simulation tools resemble real FF samples.

It may be argued that for real data we do not know biological truth based on validation experiments, but just by expert-based review. It is possible that our data contains misclassified variants, i.e. false negatives and false positives. Nevertheless, the classification was based on detailed scheme and criteria, and we performed careful manual inspection on over 5,000 SV calls. Therefore, the effect of misclassified variants on our overall results can assumed to be neglectable.

Regarding FilterFFPE, the first filtration step may seem very similar to filtering out SV calls with split-read support ≤ 2. However, these two strategies are fundamentally different. Many true SV calls in real samples lack split-read support. For example, in the 18 real FFPE samples, 41% (615/1506) of the true positive SV calls had

no split-read support. This can be related to the fact that real SVs often overlap with homologous sequences and/or sequence repeats [6]. These highly repetitive sequences can confound split-read alignment and thus obscure split-read support detection. Short read length and low coverage can also lead to missing split-read support. In addition, split-read support appears to be tool-dependent: for the same SV call we observe varying amounts of split-read support and missing information on how this number is calculated by each tool.

In real FFPE samples, we observed different levels of artifacts and thus, variable levels of PPV for SV detection. One possible reason for sample-wise artifact level variation may be different time at high temperature steps when processing FFPE samples in the laboratory. For instance, larger paraffin blocks require longer time for deparaffinization, thus leading to a higher proportion of denatured DNA and a higher number of ACRs. Besides, long-term storage of FFPE sample leads to more fragmented DNA, which is more vulnerable to denaturation at high temperature. We also observed varying sensitivities for real samples, which could be explained by different levels of sample coverage.

The mechanism of the ACR generation in FFPE samples was first described in detail by Simon Haile et al. [4]. They used S1 nuclease to remove ss-DNAs before sequencing. Their method serves as a laboratory aid, while FilterFFPE serves as a bioinformatic method to deal with these ACRs. Using S1 nuclease has the advantage of preventing ACR generation from the source. It has the ability to degrade large amounts of ss-DNA, especially in highly noisy FFPE samples. However, this can be unfavorable for low-frequency variant detection, especially considering that the FFPE material is often very precious. Moreover, the majority of already sequenced FFPE samples was not treated with S1 nuclease. And currently, to our knowledge, S1 nuclease treatment is not yet widely adopted in the standard protocol for sequencing FFPE samples.

Conclusion

SV calling in FFPE samples is challenging due to the presence of ACRs leading to a large number of false positive calls. To facilitate future development of FFPE-specific algorithms, we developed SimFFPE. It is the first simulation tool generating realistic NGS data from FFPE samples, simulating ACRs as well as normal reads. In addition, we developed the filtration algorithm FilterFFPE. Analyses on simulated as well as real data show that our algorithm successfully removes ACRs while keeping real chimeric reads. Thus, FilterFFPE improves PPV considerably without affecting sensitivity.

Availability of source code and requirements

Project name: SimFFPE and FilterFFPE
Project home page: http://www.bioconductor.org/packages/release/bioc/html/SimFFPE.html; http://www.bioconductor.org/packages/release/bioc/html/FilterFFPE.html
Operating system: Platform independent
Programming language: R
Other requirements: none
License: LGPL-3
SciCrunch ID: RRID:SCR_021085; RRID:SCR_021086

Availability of supporting data and materials

The two real data sets analyzed during the current study are available at the European Nucleotide Archive repository with accession number SRP044740 (https://www.ebi.ac.uk/ena/browser/view/SRP044740) and PRJNA301548 (https://www.ebi.ac.uk/ena/browser/view/PRJNA301548).

The three simulated data sets can be generated with SimFFPE: http://www.bioconductor.org/packages/release/bioc/html/SimFFPE.html and RSVsim: http://www.bioconductor.org/packages/release/bioc/html/RSVSim.html as described.

Additional files

Additional file 1

SupplementaryMaterial.pdf: information on supplementary methods and results.

Declarations

List of abbreviations

ACF: artifact chimeric fragment; ACR: artifact chimeric read; CNV: copy number variant; FF: fresh frozen; FFPE: formalin-fixed and paraffin-embedded; Indel: short insertion/deletion; NGS: Next-generation sequencing; PCR: polymerase chain reaction; PPV: positive predictive value; SNV: single nucleotide variant; SD: standard deviation; SRC: short reverse complementary; ss-DNA: single-stranded DNA ; SV: structural variant; WES: whole-exome sequencing; WGS: whole genome sequencing

Ethical Approval

Not applicable

Consent for publication

Not applicable

Competing Interests

The authors declare that they have no competing interests.

Funding

Not applicable

Author’s Contributions

LW analyzed the data, developed SimFFPE and FilterFFPE and wrote the manuscript. SS and MD guided the project, provided ideas for improvement during the development of the two tools, as well as suggestions on the manuscript. All authors read and approved the final version of the manuscript.

Acknowledgements

We would like to thank Dr. Marius Wöste for supporting the evaluation of SV calls.

References

1. Sah S, Chen L, Houghton J, Kemppainen J, Marko A, Zeigler R, et al. Functional DNA quantification guides accurate next-generation sequencing mutation detection in formalin-fixed, paraffin-embedded tumor biopsies. Genome medicine 2013;5(8):77.

2. Wimmer I, Tröscher A, Brunner F, Rubino S, Bien C, Weiner H, et al. Systematic evaluation of RNA quality, microarray data reliability and pathway analysis in fresh frozen and formalin-fixed paraffin-embedded tissue samples. Sci Rep 2018;8(1):6351.
3. Zhang P, Lehmann BD, Shyr Y, Guo Y. The Utilization of Formalin Fixed-Paraffin-Embedded Specimens in High Throughput Genomic Studies. Int J Genomics 2017;2017:1926304.
4. Haile S, Corbett R, Bilobram S, Bye M, Kirk H, Pandoh P, et al. Sources of erroneous sequences and artifact chimeric reads in next generation sequencing of genomic DNA from formalin-fixed paraffin-embedded samples. Nucleic Acids Res 2019;47(2):e12.
5. Kerick M, Isau M, Timmermann B, Sültmann H, Herwig R, Krobitsch S, et al. Targeted high throughput sequencing in clinical cancer settings: formaldehyde fixed-paraffin embedded (FFPE) tumor tissues, input amount and tumor heterogeneity. BMC medical genomics 2011;4:68.
6. Bartenhagen C, Dugas M. RSVSim: an R/Bioconductor package for the simulation of structural variations. Bioinformatic 2013;29(13):1679–1681.
7. Huang W, Li L, Myers J, Marth G. ART: a next-generation sequencing read simulator. Bioinformatics 2012;28(4):593–594.
8. Yu Z, Du F, Ban R, Zhang Y. SimuSCoP: reliably simulate Illumina sequencing data based on position and context dependent profiles. BMC Bioinformatic 2020;21(1):331.
9. Rausch T, Zichner T, Schlattl A, Stütz A, Benes V, Korbel J. DELLY: structural variant discovery by integrated paired-end and split-read analysis. Bioinformatic 2016;18(18):i333–i339.
10. Layer R, Chiang C, Quinlan A, Hall I. LUMPY: a probabilistic framework for structural variant discovery. Genome Biol 2014;15(6):R84.
11. Chen X, Schulz-Trieglaff O, Shaw R, Barnes B, Schlesinger F, Källberg M, et al. Manta: rapid detection of structural variants and indels for germline and cancer sequencing applications. Bioinformatic 2016;32(8):1220–1222.
12. Kosugi S, Momozawa Y, Liu X, Terao C, Kubo M, Y K. Comprehensive evaluation of structural variation detection algorithms for whole genome sequencing. Genome Biol 2019;20(1):117.
13. Gong T, Hayes V, Chan E. Detection of somatic structural variants from short-read next-generation sequencing data. Brief Bioinform 2020;.
14. Cameron D, Di Stefano L, Papenfuss A. Comprehensive evaluation and characterisation of short read general-purpose structural variant calling software. Nat Commun 2019;10(1):3240.
15. Johnson S, Trost B, Long J, Pittet V, A K. A better sequence-read simulator program for metagenomics. BMC Bioinformatics 2014;15(Suppl 9):S14.
16. Shcherbina A. FASTQSim: platform-independent data characterization and in silico read generation for NGS datasets. BMC Res Notes 2014;7:533.
17. McElroy K, Luciani F, T T. GemSIM: general, error-model based simulator of next-generation sequencing data. BMC Genomics 2012;13(1):74.
18. Angly F, Willner D, Rohwer F, Hugenholtz P, GW T. Grinder: a versatile amplicon and shotgun sequence simulator. Nucleic Acids Res 2012;40(12):e94.
19. Gourlé H, Karlsson-Lindsjö O, Hayer J, Bongcam-Rudloff E. Simulating Illumina metagenomic data with InSilicoSeq. Bioinformatics 2019;35(3):521–522.
20. Jia B, Xuan L, Cai K, Hu Z, Ma L, Wei C. NeSSM: a Next-generation Sequencing Simulator for Metagenomics. PLoS One 2013;8(10):e75448.
21. Hu X, Yuan J, Shi Y, Lu J, Liu B, Li Z, et al. pIRS: profile-based Illumina pair-end reads simulator. Bioinformatics 2012;28(11):1533–1535.
22. Pattnaik S, Gupta S, Rao A, Panda B. SInC: an accurate and fast error-model based simulator for SNPs, Indels and CNVs coupled with a read generator for short-read sequence data. BMC Bioinformatics 2014;15:40.

Click here to access/download
Supplementary Material
Revised_Supplyment_SimFFPE_and_FilterFFPE.pdf
