# Supplementary material for: SimFFPE and FilterFFPE: improving structural variant calling in FFPE samples
Source: Gigascience. 2021 Sep 22;10(9):giab065. doi: 10.1093/gigascience/giab065 (PMC8458033; doi:10.1093/gigascience/giab065)
Supplement: giab065_Supplemental_File [file giab065_supplemental_file.pdf]

SimFFPE and FilterFFPE: improving structural variant calling in FFPE samples
Supplementary Material

Lanying Wei, Sarah Sandmann and Martin Dugas

1 Mechanism of ACF formation

Figure S1 shows the mechanism how artifact chimeric fragments (ACFs) are formed. SimFFPE follows this mechanism to simulate ACFs.

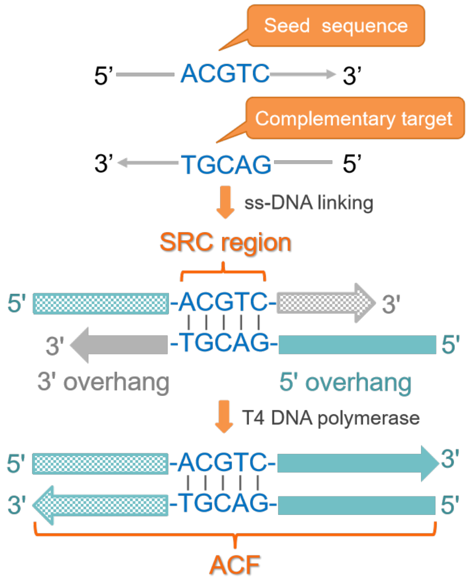

Figure S1: Example of seed- and target sequences used to generate an SRC region in an ACF. T4 DNA polymerase has 3'-5' exonuclease and 5'-3' polymerase activities. During the end-repair step of library construction, T4 DNA polymerase removes the 3' overhangs and fills in the 5' overhangs of the ss-DNA binding product to form a double-stranded ACF [1]. Abbreviations: SRC - short reverse complementary; ACF - artifact chimeric fragment.

2 Real data sets

The two real data sets analyzed in our manuscript are available at the European Nucleotide Archive repository with accession number SRP044740 and PRJNA301548. Information on these two data sets is shown in Table S1.

Table S1: Information on real data sets analyzed in our manuscript. Note: Average coverage was calculated for the final BAM files that serve as input for SV calling. Abbreviations: WES - whole exome sequencing; FF - fresh frozen; FFPE - formalin-fixed paraffin-embedded.

Data set	SRP044740		PRJNA301548	
Tumor type	Breast tumor		Unspecified	
Data type	WES			
Read length	90 bp			
Sample type	FFPE	FF	FFPE	FF
Number of samples	13	13	5	4
Average coverage	82x	58x	106x	94x
Supplementary alignment	2.10%	0.09%	3.36%	0.09%
Improperly paired reads	4.75%	1.67%	5.45%	0.77%
Reads with mate mapped to a different chromosome	2.38%	0.44%	3.76%	0.41%

3 Distributions and proportions for simulation

SimFFPE's default parameters and distributions were determined on the basis of 18 real FFPE samples. To show the differences between FFPE and FF samples, results from FF samples are also presented in this section.

Figure S2 shows the estimated length distribution of short reverse complementary (SRC) regions in FF samples. Figure S3 shows the distribution in FFPE samples. The average length of SRC regions is 10 bp in FF samples and 7 bp in FFPE samples.

SimFFPE uses a log-normal distribution (μ=1.8, σ=0.55, parameters adjustable) to approximate the SRC region length distribution in FFPE samples. Distribution of SRC region lengths in a sample simulated by SimFFPE is shown in Figure S4 (similar distributions observed in all simulated samples). The proportion of SRC regions with length $\geq$ 10 bp is smaller in simulated samples than in real FFPE samples. Due to a small window (5 kb), there is often no target sequence for long seed sequences.

The proportions of SRC pairs with two ss-DNA molecules originating from the same chromosome are shown in Figure S5. For convenience, this type of SRC pair is referred to as same chromosomal SRC pair in this document. The median proportion is 38.8% in

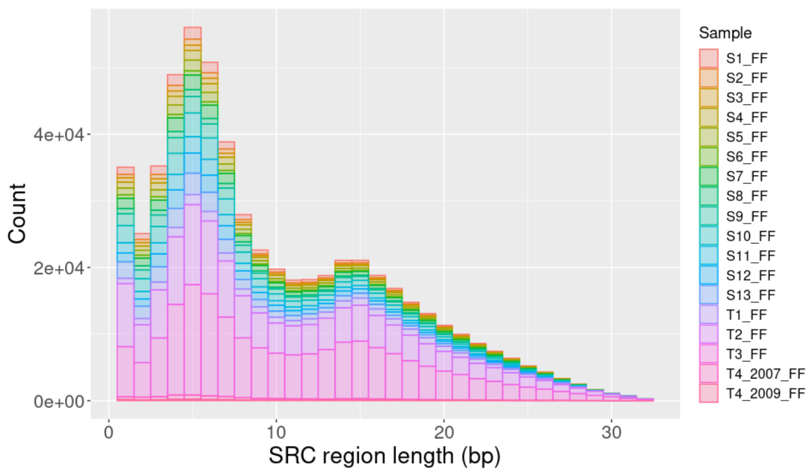

Figure S2: SRC region length distribution in real FF samples. Abbreviations: SRC - short reverse complementary; FF - fresh frozen.

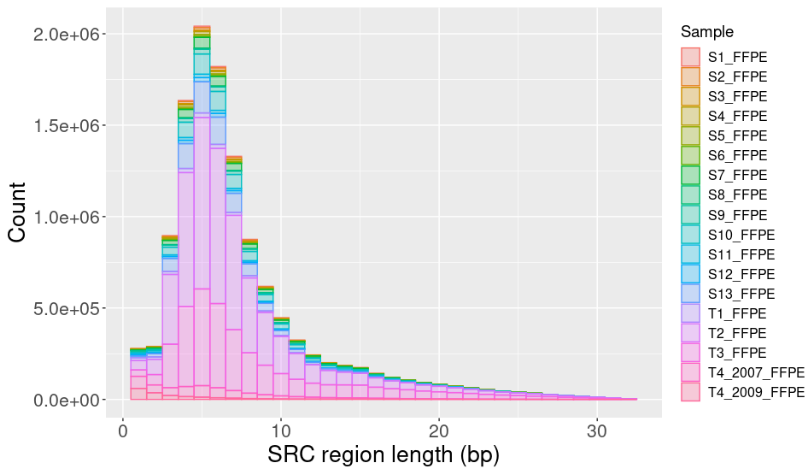

Figure S3: SRC region length distribution in real FFPE samples. Abbreviations: SRC - short reverse complementary; FFPE - formalin-fixed paraffin-embedded.

FF samples and 42.9% in FFPE samples. SimFFPE provides a parameter *sameChrProp* to simulate this proportion (default: 0.43).

Same chromosomal SRC pairs can be further divided into two categories: distant SRC pairs (original genomic distance between two ss-DNA $>$ 5 kb) and adjacent SRC pairs (original genomic distance between two ss-DNA $\leq$ 5 kb). Proportions of adjacent SRC pairs among same chromosomal SRC pairs are shown in Figure S6. The median proportion is 82.1% in FF samples and 63.4% in FFPE samples. SimFFPE provides a parameter *adjChimProp* to simulate this proportion (default: 0.63).

For distant SRC pairs, the proportions of SRC pairs derived from same- and different

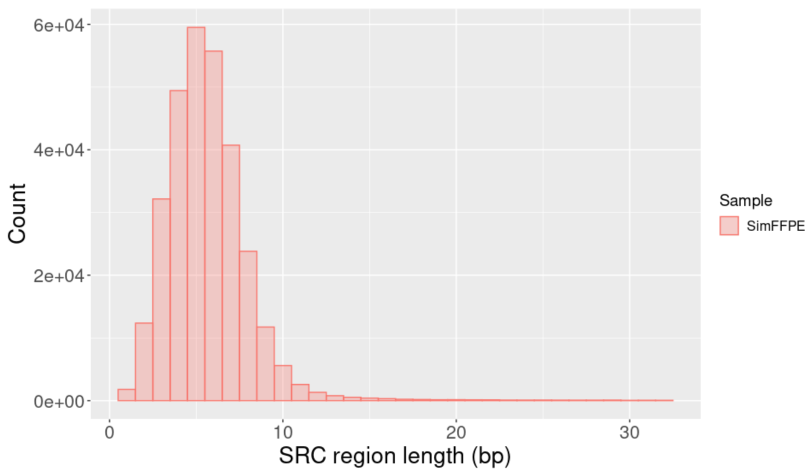

Figure S4: SRC region length distribution in an exemplary sample simulated with SimFFPE. Abbreviation: SRC - short reverse complementary.

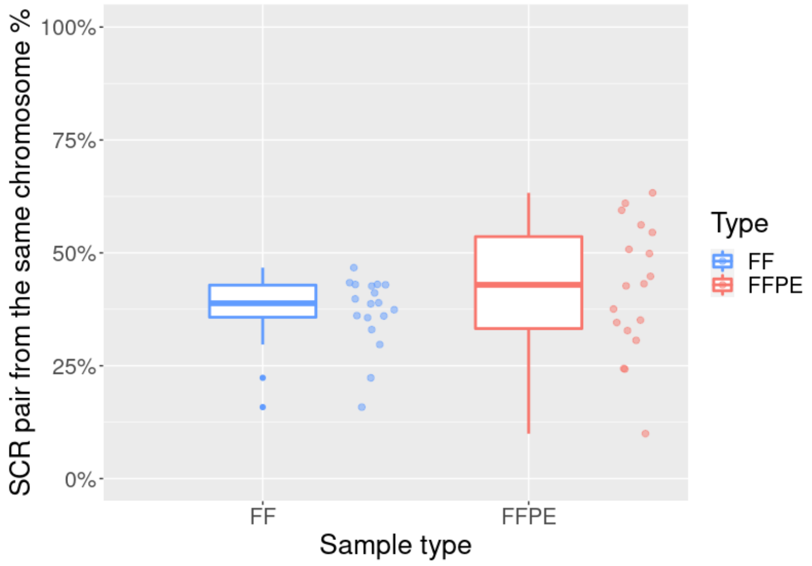

Figure S5: Proportion of SRC pairs with two ss-DNA molecules originating from the same chromosome. Each dot represents one real sample. Abbreviations: SRC - short reverse complementary; FF - fresh frozen; FFPE - formalin-fixed paraffin-embedded.

strand ss-DNA combination are equal. However, the proportion of same strand SRC pairs is higher in adjacent SRC pairs (see Figure S7). The median proportion is 63.0% in FF samples and 65.4% in FFPE samples. SimFFPE provides a parameter *sameStrandProp* to simulate this proportion (default: 0.65).

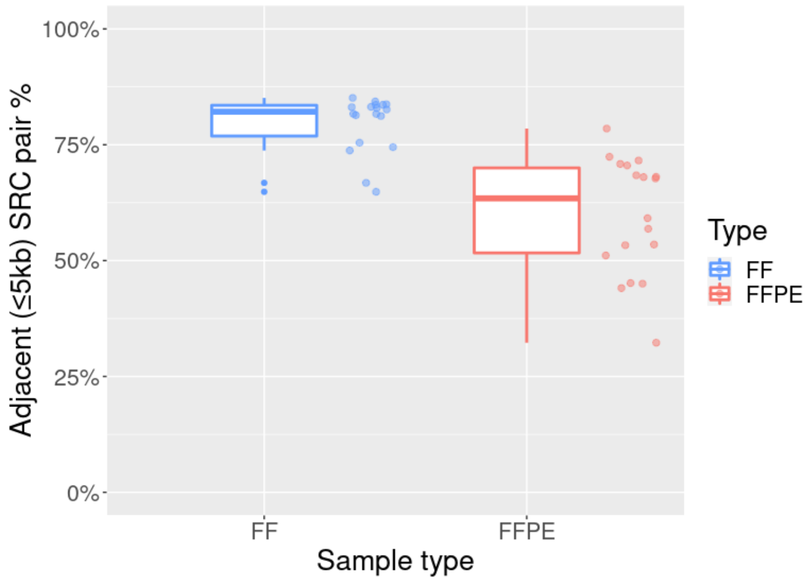

Figure S6: Proportion of adjacent SRC pairs among same chromosomal SRC pairs. Each dot represents one real sample. Abbreviations: SRC - short reverse complementary; FF - fresh frozen; FFPE - formalin-fixed paraffin-embedded.

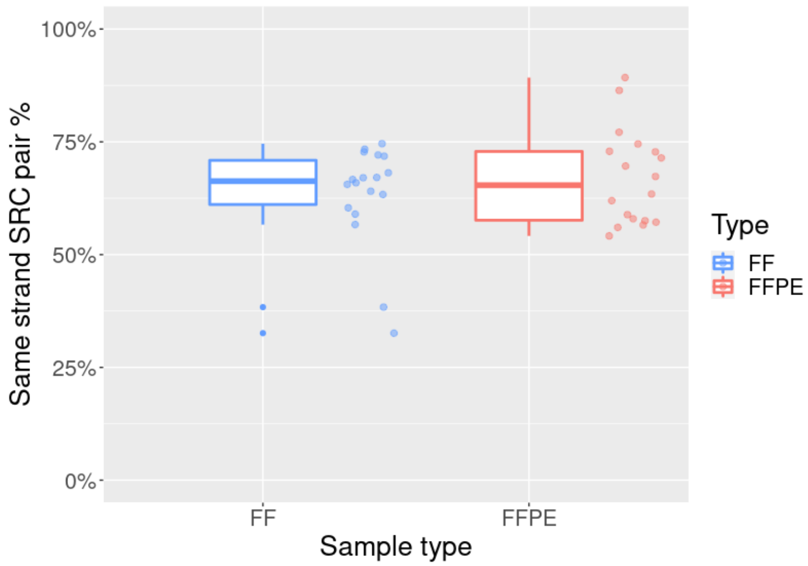

Figure S7: Proportion of same strand SRC pairs among adjacent SRC pairs. Each dot represents one real sample. Abbreviations: SRC - short reverse complementary; FF - fresh frozen; FFPE - formalin-fixed paraffin-embedded.

The original genomic distances between two ss-DNA molecules of adjacent SRC pairs are estimated. Cumulative distribution of the distance is shown in Figure S8. In FFPE

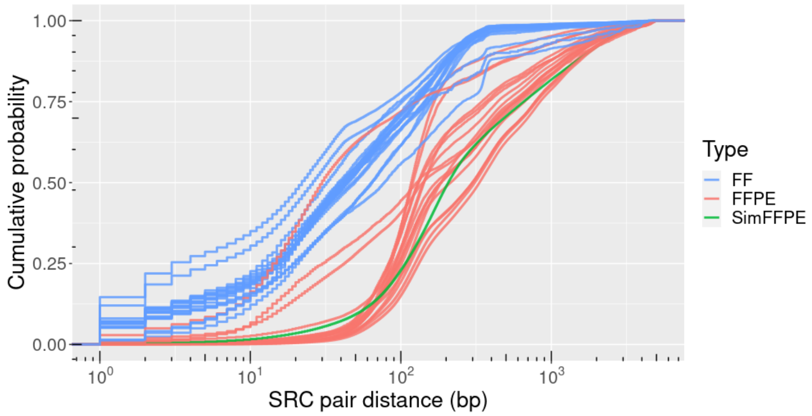

Figure S8: Cumulative distribution of the original genomic distance between two ss-DNA molecules of adjacent SRC pairs. Each line represents one real or simulated sample. Abbreviations: SRC - short reverse complementary; FF - fresh frozen; FFPE - formalin-fixed paraffin-embedded.

samples, there is a peak at around 50-200 bp. To simulate this distribution, SimFFPE applies a log-normal distribution (μ=4.7, σ=0.35, parameters adjustable) to simulate the probability of a seed binding to its target, based on its distance. The resulting cumulative distribution of an exemplary simulated sample is displayed.

4 BAM file processing

All samples were aligned to the reference genome (hg19) using BWA mem (v0.7.17) [2]. Sorting, indexing, down-sampling, and genomic regional extraction of the BAM files was performed with Samtools (v1.9) [3]. Duplicate removal was performed using Picard tools (v2.21.2, https://broadinstitute.github.io/picard/). To ensure that the supplementary alignment of duplicate reads is also excluded, before duplicate removal, the input BAM file is sorted by name and the parameter - -*ASSUME_SORT_ORDER* is set to *queryname*. Coverage estimation was performed using Mosdepth (v0.3.1) [4].

5 Manual inspection

The number of SV calls with information on initial category and final judgment is shown in Table S2. The grey list mainly contains SV calls located within highly homologous regions and a few SV calls that are difficult to classify. Typical characteristics used to determine true positive variants include:

1. Shared calls between matching FFPE and FF samples
2. Calls shared with at least one non-matched FF sample

3. Having in total ≥ 10 reads of split- and/or paired-read support
4. Breakpoints can be clearly pinpointed
5. Corresponding coverage change for deletions or insertions around breakpoints
6. Supporting reads have high mapping quality
7. Can be easily distinguished from background ACR noise

Table S2: Number of SV calls with information on initial category and final judgment.

SV call category	True positives	False positives	Grey list	Total
One	965	5	71	1,041
Two	407	1,495	380	2,282
Three	134	649	1,169	1,952
Four	0	44,877	0	44,877

Among the 2,000 randomly selected SV calls in the fourth category, 4 were considered ambiguous. These include two duplications with breakpoints located in homologous genes: KRTAP1-1 and KRTAP1-3, GYPA and GYPE; one translocation betwenn PRSS3 and its pseudogene PRSS3P2; one small duplication located in gene CRIPAK with highly repetitive coding sequences. Thus, it is possible that supporting reads of these four SV calls are in fact misaligned to homologous or repetitive sequence. Therefore, we do not consider these SV calls being possible true positives.

6 IGV view of NGS data

Figure S9 shows exemplary aligned reads generated by SimFFPE and ART [5]. For comparison, data from a real WGS FFPE sample (not publicly available due to data privacy protection) is displayed. The "spike" regions as well as enzymatic fragmented ACR pairs that map to the same locations are well simulated by SimFFPE.

Figure S10 provides an example showing that FilterFFPE successfully identifies and filters potential ACRs. Real chimeric reads that cover the breakpoints of a real SV are kept. Some non-ordinary reads are not filtered: they are considered potential real chimeric reads or have very short soft-clips (unable to form supplementary alignments), and are thus kept by FilterFFPE.

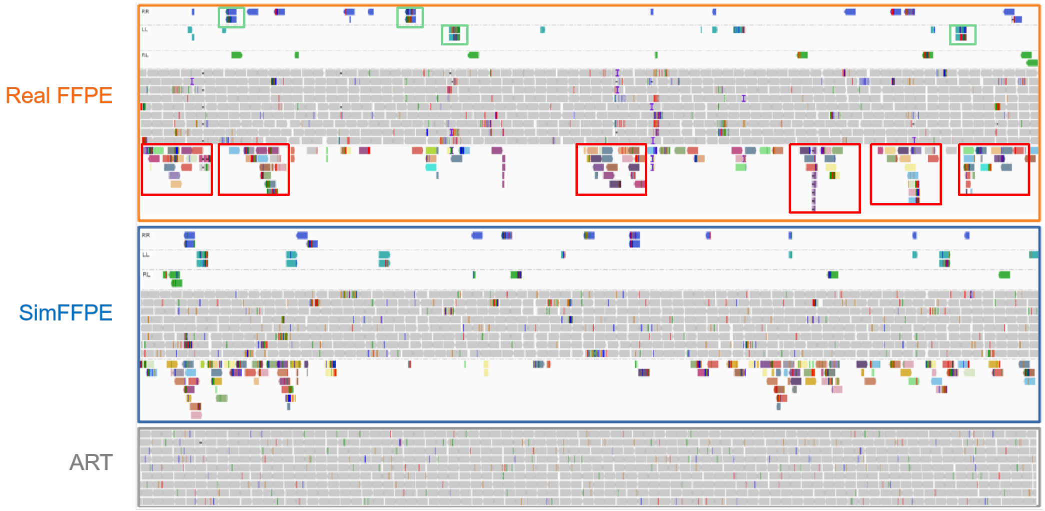

Figure S9: Exemplary alignment of reads simulated by SimFFPE and ART [5] in comparison to real reads in an FFPE sample. "Spike" regions are framed by red rectangles. ACR pairs mapped to the same locations due to enzymatic fragmentation are framed in green. Soft-clipped bases are shown. Alignments are grouped by pair orientation. Pair orientation is presented in terms of read-strand: left (L) versus right (R), and first read versus second read of a pair. The color (not gray) of the alignment indicates an abnormal pair orientation, or a different chromosome that the paired read mapped to. Alignments with normal pair orientation are colored in grey. Abbreviations: ACR - artifact chimeric read; FF - fresh frozen; FFPE - formalin-fixed paraffin-embedded.

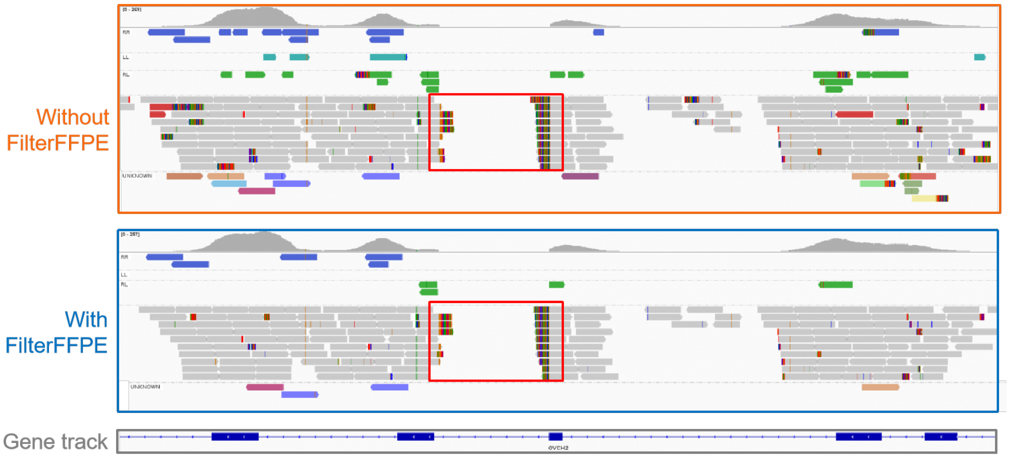

Figure S10: FilterFFPE removes artifact chimeric reads while keeping real chimeric reads. Chimeric reads of a true deletion are framed by red rectangles. Soft-clipped bases are shown. Alignments are grouped by pair orientation. Pair orientation is presented in terms of read-strand: left (L) versus right (R), and first read versus second read of a pair. The color (not gray) of the alignment indicates an abnormal pair orientation, or a different chromosome that the paired read mapped to. Alignments with normal pair orientation are colored in grey. Abbreviation: FFPE - formalin-fixed paraffin-embedded.

7 Proportion of abnormally paired reads

Proportions of abnormally paired reads in NGS data from FF, FFPE and SimFFPE's simulated samples are shown in Figures S11 and S12. To ease comparison with the varying levels of artifacts present in real data, we chose simulated samples from the Sim3 data set for our comparison, as these samples have varying fractions of ACFs.

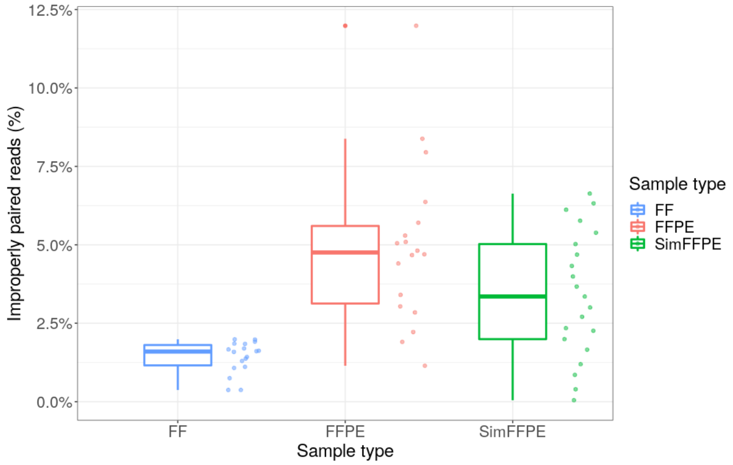

Figure S11: Proportion of improperly paired reads. Each dot represents one sample. Simulated samples originate from the Sim3 data set (with 0%-20% simulated fragments being ACFs). Abbreviations: ACF - artifact chimeric fragment; FF - fresh frozen; FFPE - formalin-fixed paraffin-embedded.

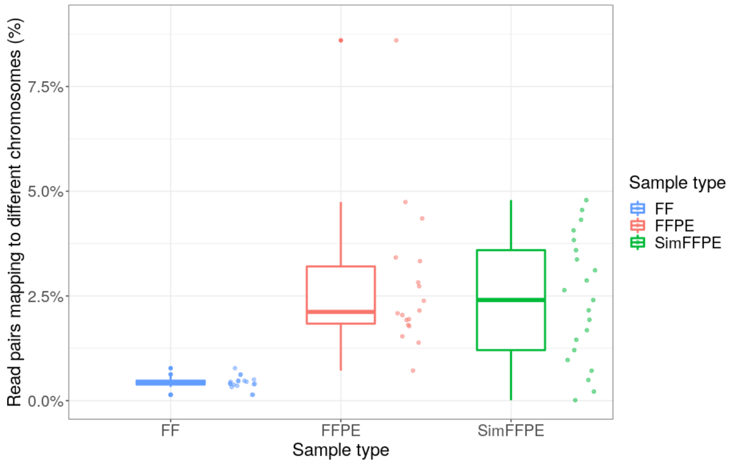

Figure S12: Proportion of read pairs mapping to different chromosomes. Each dot represents one sample. Simulated samples originate from the Sim3 data set (with 0%-20% simulated fragments being ACFs). Abbreviations: ACF - artifact chimeric fragment; FF - fresh frozen; FFPE - formalin-fixed paraffin-embedded.

8 FilterFFPE excludes FFPE-specific ACRs

Figure S13 shows the number and proportion of reads excluded by FilterFFPE.

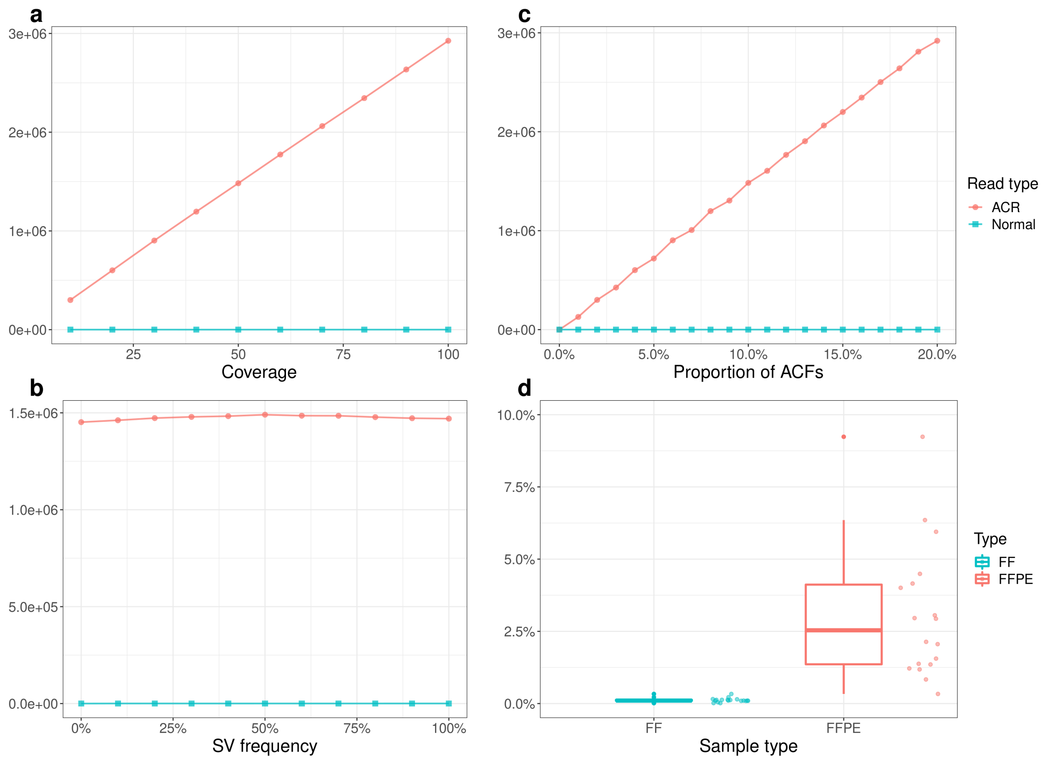

Figure S13: FilterFFPE excludes FFPE-specific ACRs. Numbers of reads excluded by FilterFFPE with two-step-filtration in simulated data set Sim1 (a), Sim2 (b), Sim3 (c) and proportions of excluded reads in real FF and FFPE samples (d) are shown. Abbreviations: ACF - artifact chimeric fragment; ACR - artifact chimeric read; FF - fresh frozen; FFPE - formalin-fixed paraffin-embedded; SV - structural variant.

The number of excluded ACRs is perfectly correlated with coverage as well as ACF proportion (Figure S13 a and c). The higher the coverage, resp. ACF proportion, the more ACRs were excluded. These results are expected as more ACRs are present if coverage or the ACF proportion is increased. Changes in SV frequency have no perceptible effect. This result is also expected as changes in SV frequency - though affecting the number of true chimeric reads - have no essential effect on the number of ACRs.

Figure S14 shows the proportion of ACRs in excluded reads for our 3 simulated data sets applying one-step and two-step filtration. Figure S15 illustrates how chimeric and non-chimeric reads can be derived from ACFs. Figure S16 and Figure S17 further display the sensitivity of FilterFFPE excluding ACRs based on chimeric reads and all reads (chimeric + non-chimeric) from ACFs, respectively. These figures show that the one-step filtration strategy is more sensitive in removing ACRs, but has the risk of removing more normal reads.

In contrast to this, the two-step filtration is characterized by slightly reduced sensitivity, ensuring that more normal reads are kept.

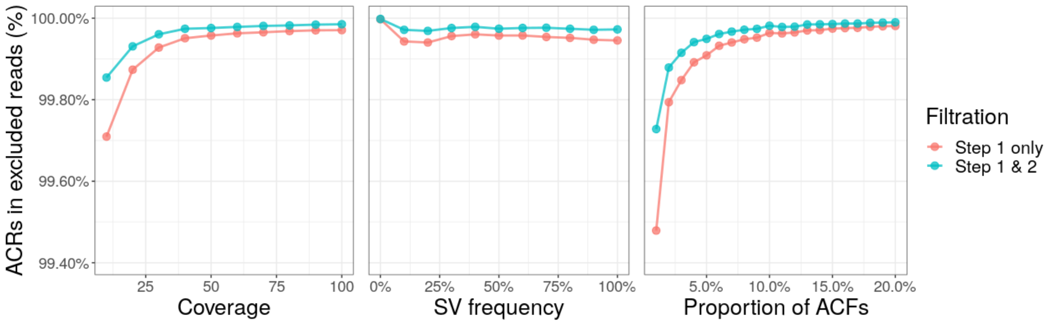

Figure S14: Proportion of ACRs in excluded reads. Proportion of ACRs in FilterFFPE's excluded reads for our three simulated data sets are shown (excluding one sample without simulated ACFs). Color indicates whether the results are generated by one-step or two-step filtration. Abbreviations: ACF - artifact chimeric fragment; ACR - artifact chimeric read; SV - structural variant.

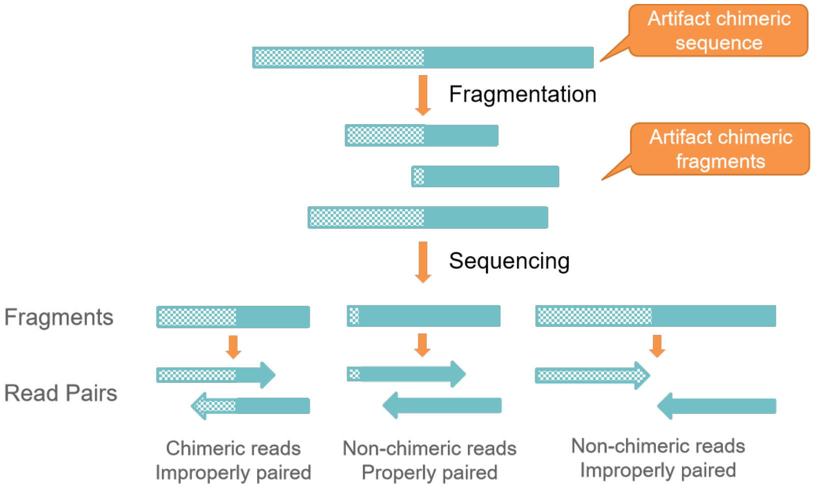

Figure S15: Examples of chimeric and non-chimeric reads deriving from artifact chimeric fragments. Here, chimeric reads specifically refers to reads with supplementary alignment. Therefore, reads with very short soft-clipped bases are non-chimeric.

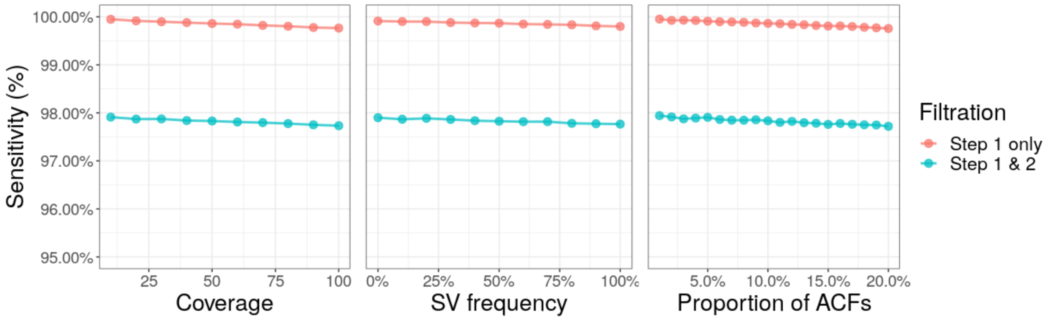

Figure S16: Sensitivity of FilterFFPE excluding ACRs based on chimeric reads (with supplementary alignment) from ACFs. Results for our three simulated data sets are shown (excluding one sample without simulated ACFs). Color indicates whether the results are generated by one-step or two-step filtration. Abbreviations: ACF - artifact chimeric fragment; SV - structural variant.

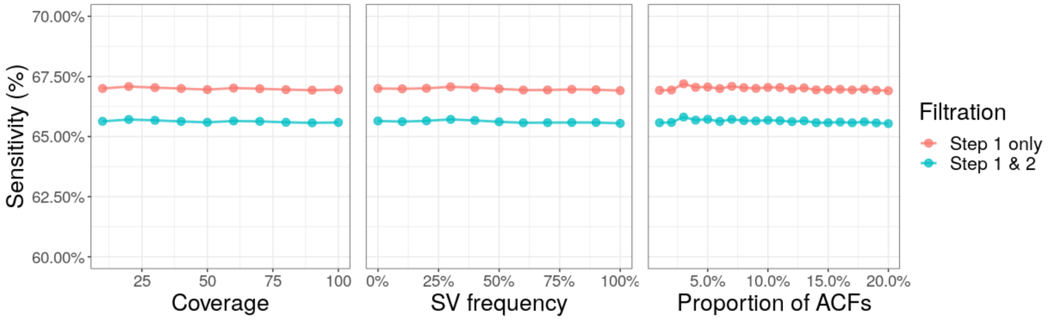

Figure S17: Sensitivity of FilterFFPE excluding ACRs based on all reads (chimeric + non-chimeric) from ACFs. Results for our three simulated data sets are shown (excluding one sample without simulated ACFs). Color indicates whether the results are generated by one-step or two-step filtration. Abbreviations: ACF - artifact chimeric fragment; SV - structural variant.

9 Evaluation of SV calling in simulated data sets

Figure S18 shows results of SV calling performance for each SV call category (*all* calls, *pass* calls, *precise* calls) in simulated data sets with and without FilterFFPE's filtering. Overall,

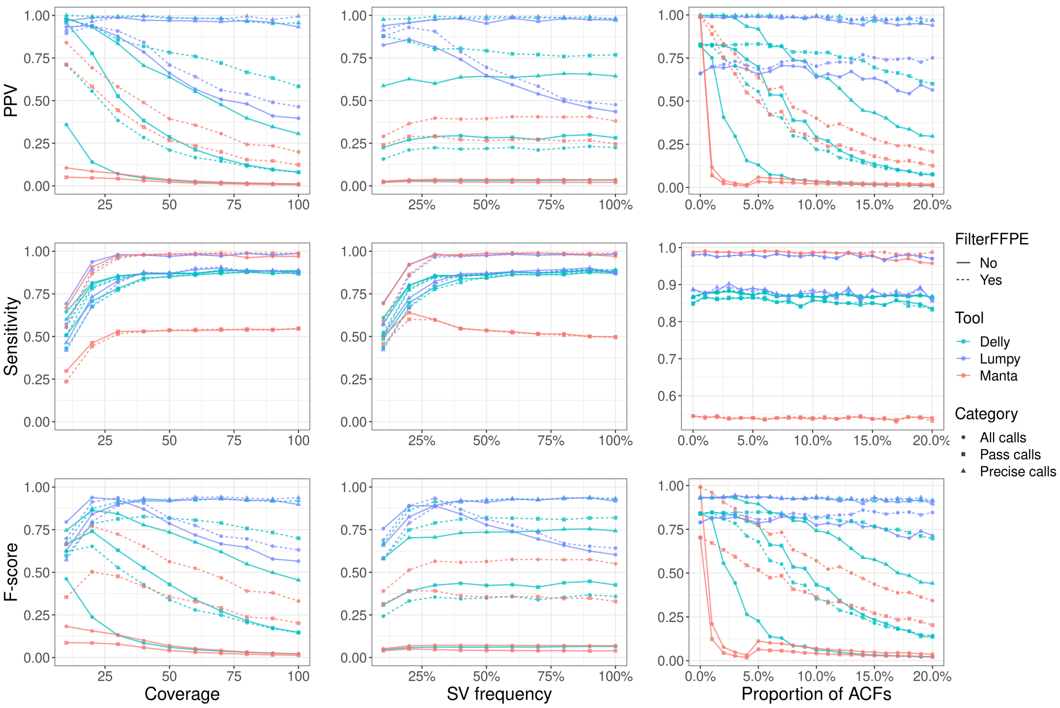

Figure S18: SV calling performance for each SV call category in simulated data sets with and without FilterFFPE's application (two filtration steps applied). Abbreviations: ACF - artifact chimeric fragment; PPV - positive predictive value; SV - structural variant.

Delly's best performance is observed for FilterFFPE+Delly, considering only *precise* calls (average F1 score improves from 0.71 to 0.91 with FilterFFPE's application). Lumpy's best performance is also observed for FilterFFPE+Lumpy, considering only *precise* calls. However, the average F1 score is only marginally improved by FilterFFPE's application (from 0.9029 to 0.9032). Manta's best performance is observed for FilterFFPE+Manta, considering all calls. FilterFFPE largely improves Manta's performance (average F1 score improves from 0.09 to 0.58 with FilterFFPE's application).

Results on filtration with FilterFFPE in simulated samples applying the first step only are displayed in Figure S19. It is worth mentioning that only few SV calls of Manta are not labeled as *precise* calls (0.2% to 2% on average across all simulated and real data sets before applying FilterFFPE). Consequently, the set of all calls hardly differs from the set only containing the *precise* calls. Therefore, we decided not to include Manta's *precise* category for evaluation. Lumpy does not provide the *pass* / *non-pass* category in the output.

Overall, Delly's best performance is still observed for FilterFFPE+Delly, considering only *precise* calls (average F1 score improves from 0.71 to 0.90 with FilterFFPE's applica-

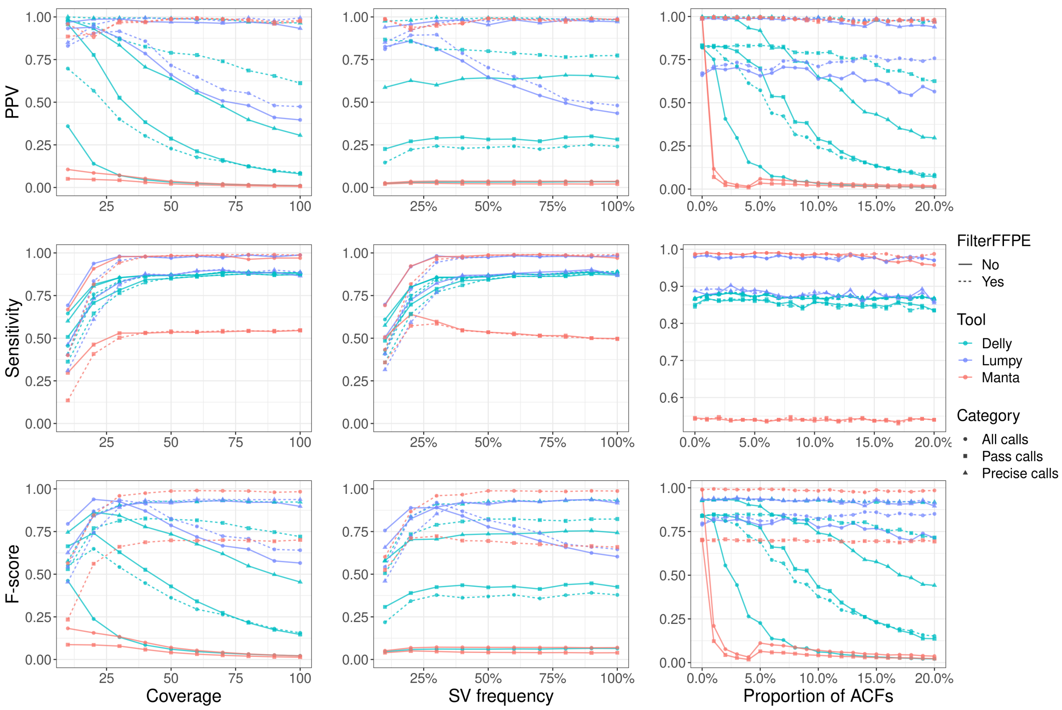

Figure S19: SV calling performance for each SV call category in simulated data sets with and without applying FilterFFPE's first filtering step only. Abbreviations: ACF - artifact chimeric fragment; PPV - positive predictive value; SV - structural variant.

tion). Lumpy's best performance is observed for Lumpy without FilterFFPE, considering only *precise* calls. The average F1 score is marginally reduced by FilterFFPE's application (from 0.90 to 0.89). It can be observed that in the 6 samples with low coverage ($\leq$ 30x) or low SV frequency ($\leq$ 30%), sensitivity is lower for the results based on one-step filtration by FilterFFPE, compared to two-step-filtration. This shows that the second filtering step of FilterFFPE has achieved its expected effect (improving sensitivity in case of low coverage or low SV frequency).

Manta's best performance is still observed for FilterFFPE+Manta, considering all calls. FilterFFPE improves Manta's performance to a greater extent, even outperforming Delly and Lumpy's best performance (average F1 score improves from 0.09 to 0.96 with FilterFFPE's application). It can be observed that Manta is characterized by highest sensitivity. However, PPV is low because Manta is too sensitive to ACRs. Applying the first filtration step only leads to more stringent filtering. With the vast majority of ACRs filtered, PPV of Manta's results largely increases. However, this result should be treated with caution because it can be difficult to extensively exclude ACRs in real data.

In addition, we split the simulated data into samples with low coverage or low SV frequency (n=6, with coverage $\leq$ 30 or SV frequency $\leq$ 0.3) and other samples (n=35). Figures S20 and S21 show the performance of one- and two-step filtration for these two

cases, respectively.

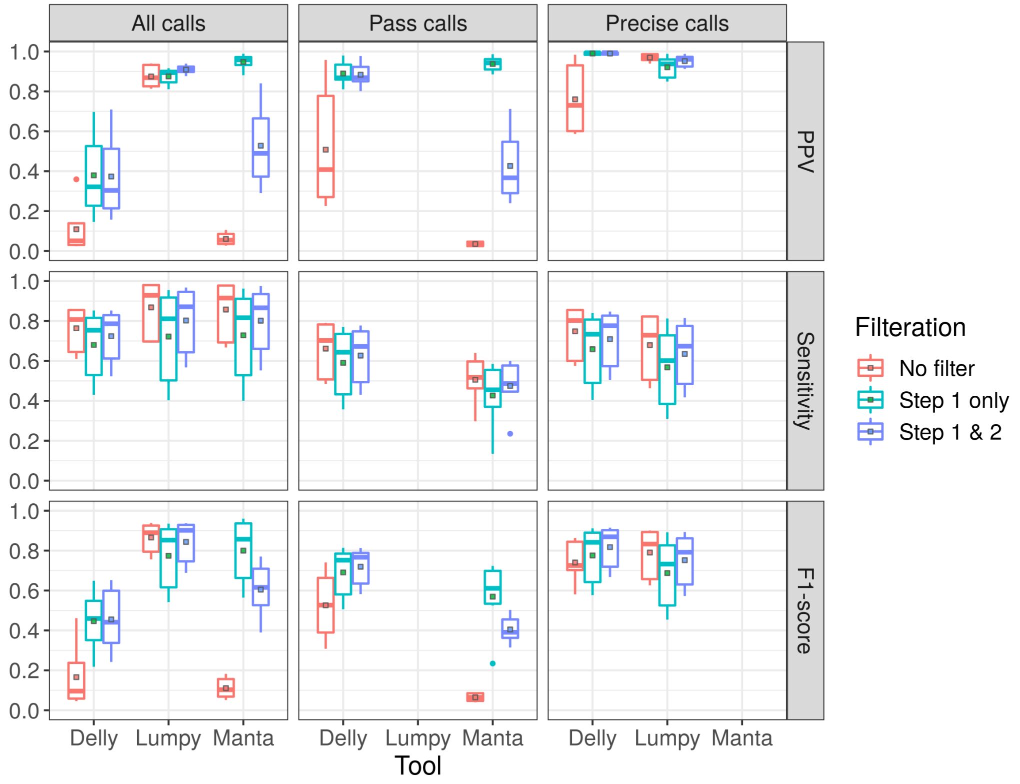

Figure S20: SV calling performance for 6 simulated samples with low coverage or low SV frequency. Results with (one- or two -step filtration) and without FilterFFPE's application are shown. The 6 samples are characterized by coverage $\leq$ 30x or SV frequency $\leq$ 0.3. Abbreviations: PPV - positive predictive value.

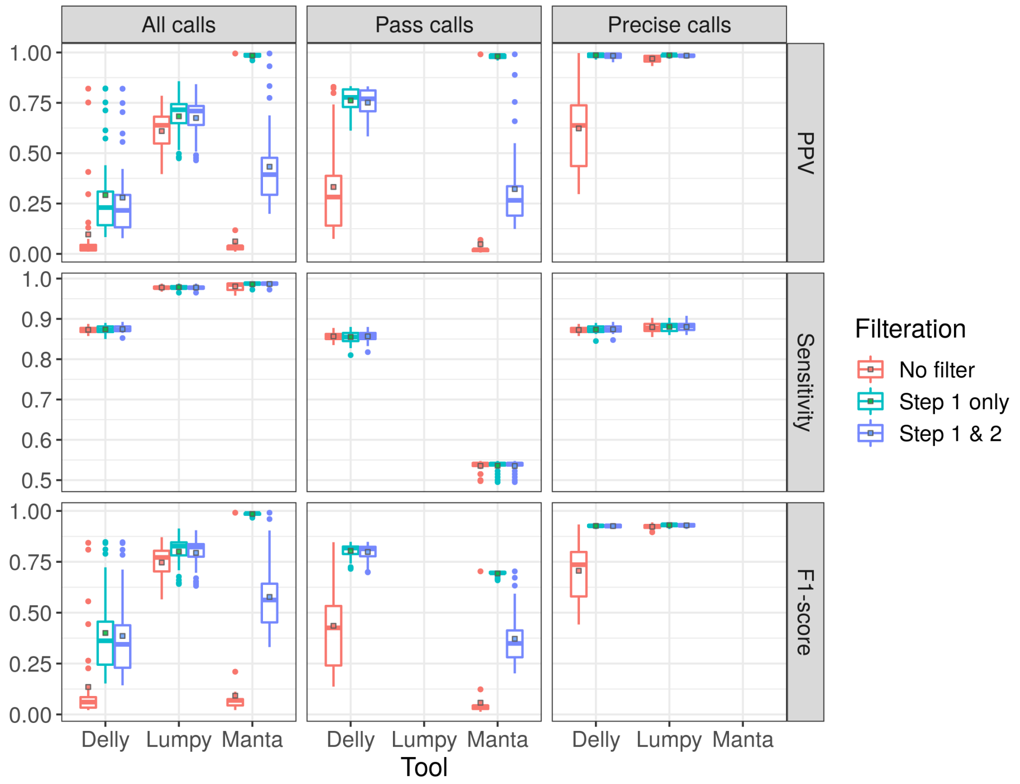

Figure S21: SV calling performance for 35 simulated samples with high coverage and high SV frequency. Results with (one- or two -step filtration) and without FilterFFPE's application are shown. The 35 samples are characterized by coverage > 30x and SV frequency > 0.3. Abbreviations: PPV - positive predictive value.

10 Evaluation of SV calling in real data sets

Figure S22 shows results of SV calling performance in real data with (one- or two-step filtration) and without application of FilterFFPE.

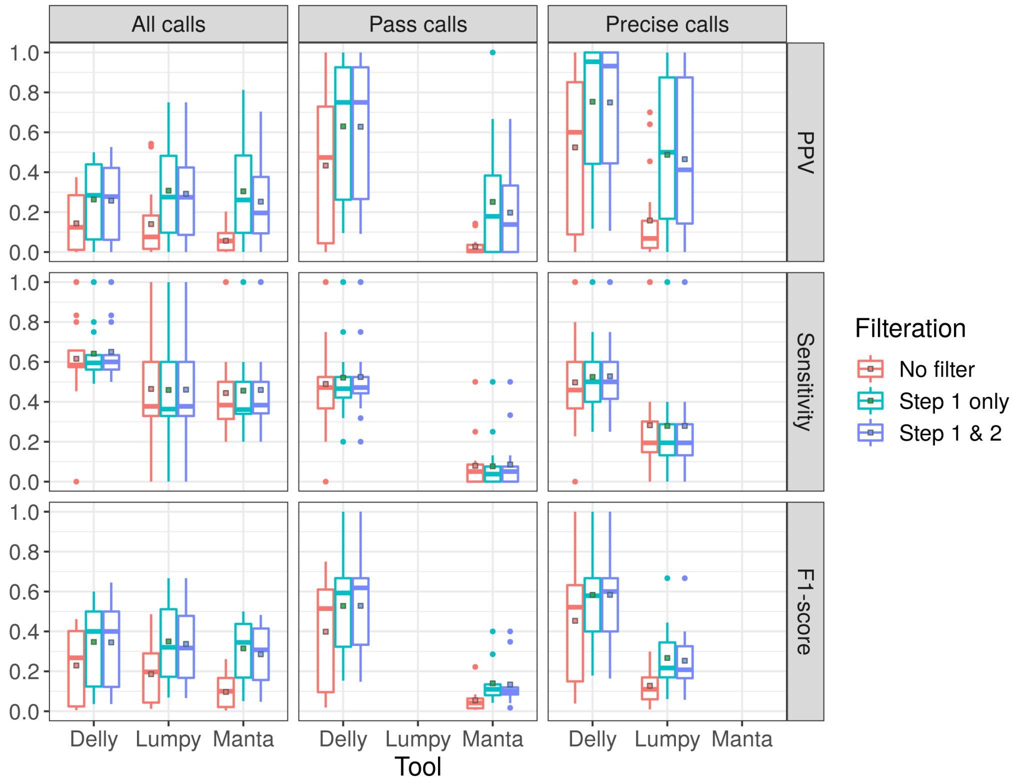

Figure S22: SV calling performance for real data with (one- or two -step filtration) and without FilterFFPE's application. Abbreviations: PPV - positive predictive value.

It can be observed that FilterFFPE improves F1-score in all three tools considering *all* calls, only *pass* calls or *precise* calls. With two-step filtration, Delly's best performance is observed - just like in simulated data - for FilterFFPE+Delly, considering only *precise* calls (average F1 score improves from 0.45 to 0.58 with FilterFFPE's application). Lumpy's best performance is observed for FilterFFPE+Lumpy's all calls (average F1 score improves from 0.19 to 0.34 with FilterFFPE's application). Manta's best performance is also observed for FilterFFPE+Manta's all calls (average F1 score improves from 0.10 to 0.29 with FilterFFPE's application). Just like in simulated data sets, Manta shows the largest improvement with FilterFFPE among these three tools.

Just like in case of simulated samples, decreased sensitivity and increased PPV could be observed when only applying the first filtering step. Notably, for real data sets, we filtered out exonic regions with average coverage below 30x in FFPE or matched FF samples before SV calling. The second filtering step mainly improves sensitivity at low coverage or low SV

frequency; thereby, the improvement in sensitivity applying the second filtering step is small in real data sets, while the improvement in PPV using only the first filtering step is more pronounced.

Figure S23 shows the relative change in the number of SV calls in real data after application of FilterFFPE (one-step and two-step filtration).

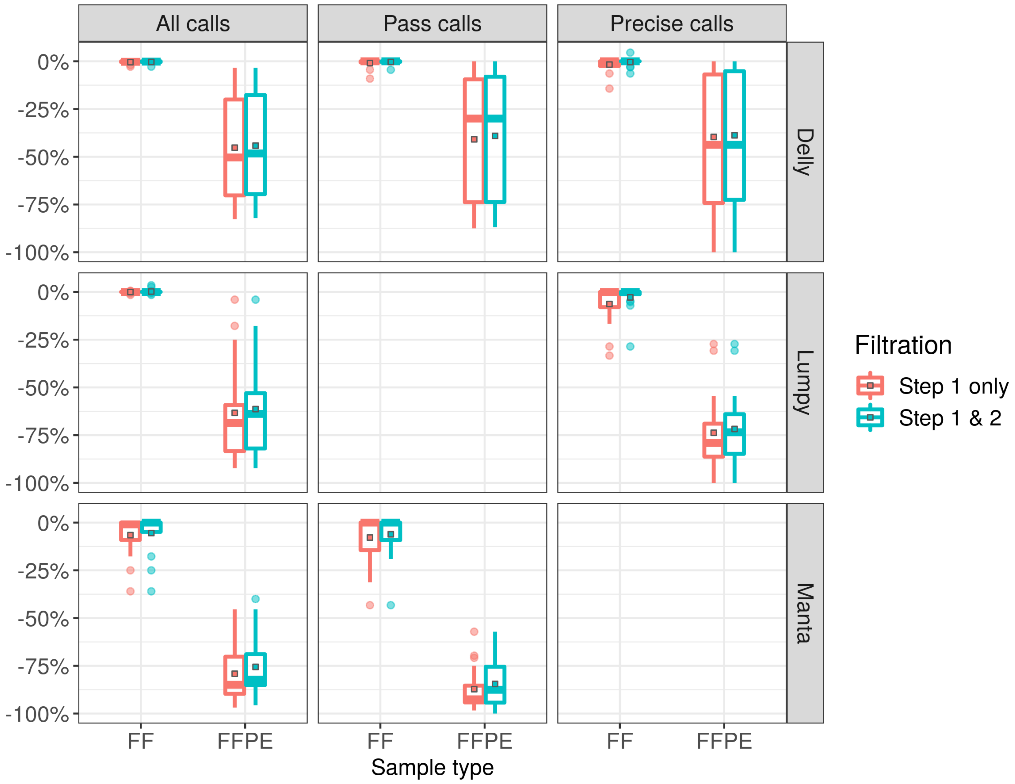

Figure S23: Relative change in the number of SV calls in real data after application of FilterFFPE (one-step and two-step filtration). Abbreviations: FF - fresh frozen; FFPE - formalin-fixed paraffin-embedded.

With the application of FilterFFPE, the number of SV calls in FFPE samples decreases substantially. In FF samples, only slight changes could be observed. Of the three tools we evaluated, Manta shows the largest decrease in SV calls with application of FilterFFPE. This corresponds to our previous observation that Manta is most sensitive to ACR noise.

11 Increased sensitivity after application of FilterFFPE

Tables S3 and S4 show the number of true calls for each tool that are exclusively called before and after the application of FilterFFPE with one-step filtration and two-step filtration, respectively.

Table S3: Number of true calls that are exclusively called before and after the application of FilterFFPE with one-step filtration. All reported calls from the 18 real FFPE samples are considered. Abbreviation: FF - fresh frozen.

	No filtration			One-step filtration		
	Shared with matched FF		Total	Shared with matched FF		Total
Tool	Yes	No		Yes	No	
Delly	6	9	15	11	6	17
Lumpy	8	8	16	6	7	13
Manta	8	11	19	4	21	25
Sum	22	28	50	21	34	55

Table S4: Number of true calls that are exclusively called before and after the application of FilterFFPE with two-step filtration. All reported calls from the 18 real FFPE samples are considered. Abbreviation: FF - fresh frozen.

	No filtration			Two-step filtration		
	Shared with matched FF		Total	Shared with matched FF		Total
Tool	Yes	No		Yes	No	
Delly	3	6	9	9	6	15
Lumpy	4	8	12	3	7	10
Manta	6	9	15	4	20	24
Sum	13	23	36	16	33	49

These results correspond to the changes in sensitivity for Delly, Lumpy and Manta after application of FilterFFPE. For Manta, 21 non-shared (not shared with matched FF samples) true SV calls were exclusively detected after application of FilterFFPE. Nevertheless, 14/21 of these SV calls are shared with at least one other FFPE/FF sample. For the remaining 7 SV calls, 3 have more than 10 reads of paired-read support.

Increased sensitivity was also observed for Manta in case of simulated samples (not low coverage or low SV frequency). In summary, our results show that in case of sufficient coverage and SV frequency, several additional true positive SVs were detected by Delly and Manta after removing ACRs by the help of FilterFFPE. Thus, ACRs do not only generate false positive SV calls, but can also affect the detection of true SV calls. Nevertheless, according to the results of the simulated data (Figure S18, changes of sensitivity with increasing coverage or ACF proportion), this influence is rather small.

References

[1] Haile S, Corbett R, Bilobram S, Bye M, Kirk H, Pandoh P, et al. Sources of erroneous sequences and artifact chimeric reads in next generation sequencing of genomic DNA from formalin-fixed paraffin-embedded samples. Nucleic Acids Res. 2019;47(2):e12.

[2] Li H, Durbin R. Fast and accurate short read alignment with Burrows-Wheeler transform. Bioinformatics. 2009;25(14):1754–1760.

[3] Li H, Handsaker B, Wysoker A, Fennell T, Ruan J, Homer N, et al. The Sequence Alignment/Map format and SAMtools. Bioinformatics. 2009;25(16):2078–2079.

[4] Pedersen B, Quinlan A. Mosdepth: quick coverage calculation for genomes and exomes. Bioinformatics. 2018;34(5):867–868.

[5] Huang W, Li L, Myers J, Marth G. ART: a next-generation sequencing read simulator. Bioinformatics. 2012;28(4):593–594.
