# Supplementary material for: SimFFPE and FilterFFPE: improving structural variant calling in FFPE samples
Source: Gigascience. 2021 Sep 22;10(9):giab065. doi: 10.1093/gigascience/giab065 (PMC8458033; doi:10.1093/gigascience/giab065)
Supplement: giab065_Response_to_Reviewer_Comments_Original_Submission [file giab065_response_to_reviewer_comments_original_submission.pdf]

Author's Response To Reviewer Comments

Close

Reviewer #1:
Specific comments/questions:
* Evaluating the fidelity of simulated NGS data in general is very difficult. And in this context many of the parameters you might compare in such an evaluation (i.e. those in Figure 2) were used to develop the tool. However, are there any other metrics you could use to quantitatively evaluate SimFFPE? For example, could a comparison between real and simulated FFPE data of the the fraction of improperly paired reads (or pairs mapping to different chromosomes) in tiled windows across the genome provide evidence of the fidelity of SimFFPE? Alternately are there experimental evaluations that might be possible, but would be beyond the scope of this work, which could be included as future work in the discussion?

Changes: We added section 7 to the supplementary material, describing our analysis of the “Proportion of abnormally paired reads”, and refer to it at the end of subsection “Simulating realistic FFPE data using SimFFPE” of the main article.

We think it is a good suggestion to use these metrics to quantitatively evaluate SimFFPE. Results regarding this new analysis underline our previous observation that SimFFPE generates realistic simulated FFPE data.

* I had trouble understanding the manual curation process/results, and specifically category 3 SVs. My understanding is that this category comprises SVs that do not match a call in a FF sample and have less than 10 supporting reads. And that the authors manually reviewed 1,952 of these 46,829 SVs. Should I interpret Table S2 as 134/1,952 were actually true positives and 1,169/1,952 were considered ambiguous (and thus excluded)? Or is the "grey list" determined algorithmically? If the former, what fraction of the putative false positives (most of which, if I understood correctly, were not manually reviewed) would still be considered false positives (as opposed to "grey listed") had they been manually reviewed? Since there are so many putative false positives, it would seem that this category would have a significant impact on the results.

Changes: We revised our description in the main manuscript (section “Performance evaluation”) and supplement (section 5) and updated our categorization: now, there are four categories. Former category 3 is split into category 3 (low probability of being true; all calls manually reviewed) and category 4 (calls automatically labeled as false positives; random examples manually reviewed). Category 3 contains 1,952 calls, category 4 contains 44,877 calls. To access the fidelity of our automatic classification (category 4) we manually reviewed 2,000 randomly selected SV.

We totally agree that 44,877 putative false positive SV calls that are not reviewed could have a significant impact on the results. Therefore, we extended our manual review of variant calls and additionally considered 2,000 randomly selected calls in category 4. Among these, 1,996 are false positives and only 4 are considered ambiguous (located in homologous / repetitive regions). The 4 ambitious calls are kept after application of FilterFFPE. Our updated results confirm that the expected number of true positives within these 44,877 category 4 calls is very low (0-90). Therefore, we think it is reasonable to label the whole category 4 as false positives without further manual inspection.

* Page 5 and Figure S11: I find the absolute scales in Figure S11 panels a-c hard to interpret since there is no context to know how many ACRs should have been excluded. I would advocate for reporting the fraction of ACR and non-ACR reads excluded. This would complement the results on Page 5, which if I understood correctly, are effectively the precision/PPV ("99.73% to 100.00% of the filtered reads were ACRs (average: 99.96%)."). Is the sensitivity also reported somewhere? If not, I think including those stats in that paragraph would help the reader better understand FilterFFPE's effectiveness.

Changes: We added four new figures to our supplement showing PPV and sensitivity of FilterFFPE (Figures S14-S17). Additionally, we updated the subsection “Filtering FFPE-specific ACRs with FilterFFPE” in our main manuscript (2nd paragraph) by these additional results.

* I found the evaluation of the two filtering steps very interesting, and particularly the sentences on page 11 in the supplemental "This shows that the second filtering step of FilterFFPE has achieved its expected effect (improving sensitivity in case of low coverage or low SV frequency)." and on page 13 in the supplemental: "The second filtering step mainly improves sensitivity at low coverage or low SV frequency; thereby, the improvement in sensitivity applying the second filtering step is small in real data sets, while the improvement in PPV using only the first filtering step is more pronounced." The combination of those sentences prompted a number of questions:

- Does this indicate that stage 1 alone excludes more than just ACRs, but that effect is attenuated at increased coverage?

Yes, in the new Figure S14 you can also observe this tendency: With increased coverage, more reads can cover the breakpoint of an SV event. Therefore, the likelihood of these reads to be removed as artifact chimeric reads by mistake gets smaller.

- What fraction of reads that would have been excluded by stage 1 only and retained when stage 2 is employed (if I understood correctly, theses are reads with unique breakpoints but no SRC region)? Or perhaps more useful for the reader, what are the precision and recall for excluding ACRs with the different filter configurations?

Changes: We updated results on application of no filter, one- and two-step filtration to enable the direct comparison (see Figures S20-S23 in supplementary material).

Regarding your question: Yes, those are reads with unique breakpoints but no SRC region.

- For the real data (with sufficient coverage) does looking for the SRC regions during filtering have either a neutral or actually a net negative effects on SV calling?

Changes: We updated supplementary Figure S22. It can be observed that adding the second filtration step results in slightly lower PPV and slightly higher sensitivity.

- If there are indeed a relevant fraction of ACRs without SRC regions, is there a different mechanism that creates those reads? Is it the case that the SRC region is present but can't be detected (as suggested on page 5: "However, sequencing noise in ACRs may harm the correct detection of SRC regions.") or are there other error mechanisms at work?

Currently we have not observed or had theories about any other mechanism. In addition, verification of possible mechanisms requires laboratory work, which is beyond the scope of this work.

I struggled to compare the effects of the two filter stages using Figures S12-S13, S14-S15, and S16-S17. I found myself flipping back and forth, but it is difficult to compare the values that way. Could Figures S14-S15 be integrated to show "No filter", "Stage 1 only" and "Stage 1 & 2" (i.e., three bars instead of two) to permit direct comparison? And perhaps something similar for Figures S16-S17? I recognize that doing the same for Figures S12-S13 might become unreadable. Is there another way to structure FigureS13 to more directly show the differences between the two filter configurations?

Changes: We now integrated results with no filter, with one- and two-step filtration into a single figure to enable the direct comparison (see Figures S20-S23 in supplementary material).

For Figures S18 and S19 (former Figures S12 and S13), it is difficult to merge the results in one readable figure. Therefore, we provided two additional Figures S20 and S21 to enable direct comparison. Since the second filtration step is specifically designed for low coverage or low SV frequency, we split the simulated samples into 6 samples with low coverage / low SV frequency, and 35 other samples. This

allows the reader to compare the effect of one- and two-step filtration in these two contexts.

* The sections above prompted me to wonder if "stage 1" is a useful filter to apply prior to SV calling generally, not just for FFPE samples (at least with high coverage). How does stage 1 vs. stage 1 and 2 impact the sensitivity and PPV for SV calls in the FF samples? If stage 1 is an interesting filter "across the board", perhaps add a brief description of its utility to the discussion?

Although we like the idea of introducing 1-step filtration with FilterFFPE as a general step in the workflow of SV calling, we do not observe much evidence that it would actually be beneficial. In Figure S23 it can be observed that FilterFFPE has only little effect on FF samples, especially for Delly and Lumpy.

* My understanding is that FilterFFPE is removing reads and thus my initial (and likely naive) expectation is that sensitivity would be the same or decrease - not increase. If I understood correctly, the last paragraph of the results and Figure S16 indicates that after filtering all the callers are reporting fewer SVs. And from Page 7 my understanding is that all SVs (not just PASSing SVs) are considered in the analysis. If that is correct, does that indicate the callers are identifying more/different true positive SVs in the FilterFFPE data than in all the data?

Changes: We added section 11 “Increased sensitivity after application of FilterFFPE” to our supplement. Tables S3 and S4 within this section sum up the number of true calls for each tool that are exclusively called before and after FilterFFPE’s application. In the main article, we updated the corresponding paragraph in our results section accordingly.

* Page 7: "Despite developing a tool for realistic simulation of FFPE samples, it can be observed that sensitivity of the three SV calling tools Manta, Delly and Lumpy differed between simulated and real data. These discrepancies were mainly due to technical differences between these data sets: our simulated samples were whole chromosome sequencing data (mimicking WGS data since it is the ideal material for SV calling) while real samples contained WES data and had a shorter read length (150 bp in simulated samples vs 90 bp in real samples)." I am curious why you did not try to generate simulated data similar to the real samples. My understanding is that SimFFPE supports both WGS and WES simulation.

Changes: We updated subsection “Simulated data sets” of our methods section. To complement the real WES data that we evaluated in our manuscript, we additionally generated simulated data that are more optimal for SV calling (mimicking WGS data with 150 bp read length, n=41).

Minor comments/questions:

* While the choice of Lumpy, Delly, and Manta seems very appropriate for the simulated WGS data, my understanding is that those methods are not necessarily designed for WES/targeted sequencing. I recognize though that the key comparisons here are within tools (with and without FilterFFPE) as opposed to absolute sensitivity, etc. Out of curiosity, do you think you would observe similar results for WES-specific CNV callers like XHMM, etc.?

We think that it mainly depends on the sources of evidence deployed by the caller to detect SVs: Since the majority of WES-specific CNV callers (including XHMM) only use changes in read-depth (depth of coverage) to detect CNVs, we do not expect ACRs (and thus FilterFFPE) to have much impact on the results. However, in case of WES-specific CNV callers also integrating split-read and/or paired-read evidence, FillerFFPE should have similar effect on the results.

* When simulating WES data, how is the effect of the WES capture technology modeled?

Changes: We updated the description of “Simulating ACRs with SimFFPE”, mentioning that the capture efficacy is uniformly modeled.

A more realistic simulation of the capture efficacy variation might be obtained by analyzing a large

amount of WES (FFPE) data, but since it is not the focus of this work, we only used a simple simulation.

* Page 3: "In real data, we observed that some read pairs from adjacent ACFs both align to the same genomic locus. We found out that this is a special phenomenon arising from the enzymatic fragmentation of some adjacent ACFs…" I had trouble following the phenomenon being described in this section. Perhaps a naive question, but is this a function of the specific library preparation method, or does this occur generally in FFPE samples? How does SimFFPE model the fragmentation location?

Changes: We modified the description in our methods section, subsection "Simulating ACFs" to make it clearer.

Regarding your question: Yes, it is due to the specific library preparation method.

* I found Figure 5 confusing at first. Based on the convergence of arrows at "SV calling" I initially thought that the simulated data was used during evaluation of the real FFPE and FF samples. But if I understood correctly those are entirely separate analyses. If that is indeed correct, I would suggest having separate a "SV calling" box for the for the simulated and real data workflows.

Changes: We modified the figure accordingly.

* I find Figure 8 difficult to interpret since you have to match the dots manually based on the very small text. Is the intent for the reader to look at the shift of individual points, or to look at the shift in the overall distribution? If the latter, is there an alternate visualization of the distribution? Or perhaps overlay a cross with the means, quartiles, etc.?

Changes: Thank you for this suggestion. The shift in the overall distribution is more important. We modified the figure, removed text labels and added box plots to the sides for better visualization.

* Page 7: I would advocate for including the standard deviation along with the means (or the range of changes observed) to provide additional context for interpreting the changes in sensitivity and PPV before and after applying FilterFFPE.

Changes: Standard deviation was added to the text.

Reviewer #2:

Major comments

1. On pages 4-5, under section 'Simulating ACFs', the authors mention a higher probability of SCRs forming between adjacent chromosomal regions or within the same chromosome, based on 18 real FFPE samples. However, the Figure 2 indicates the probability of SCRs on the same chromosome as 43%, and that on different chromosomes as 57%.

Changes: We updated the description of "Simulating ACFs" in our methods section.

We see your point that the probability of SCRs on the same chromosome is – altogether – lower compared to different chromosomes. In our description in the methods we just wanted to point out that 27% (adjacent region on the same chromosome) is a relatively high number. It has to be taken into account that the length of one chromosome is much shorter than the total length of all other chromosomes, and even much shorter is the length of an adjacent region on one chromosome. Furthermore, a higher percentage can be observed for the same strand within the group of adjacent regions on the same chromosome. Within the group of regions on different chromosomes, the percentage regarding the strand is equal (50%).

2. Figure 8 shows sample-wise and tool-wise differences in relationship between positive predictive values and sensitivity of SV detection, using real data. It is not clear what causes sample-to-sample

differences in this relationship. Can the authors elaborate further?

Changes: We added a paragraph to our discussion (the 2nd to last paragraph) elaborating on this question.

Close
