# Supplementary material for: SimFFPE and FilterFFPE: improving structural variant calling in FFPE samples
Source: Gigascience. 2021 Sep 22;10(9):giab065. doi: 10.1093/gigascience/giab065 (PMC8458033; doi:10.1093/gigascience/giab065)
Supplement: giab065_Response_to_Reviewer_Comments_Revision_1 [file giab065_response_to_reviewer_comments_revision_1.pdf]

Author's Response To Reviewer Comments

Close

Thanks to reviewers and editors for all the comments and suggestions. The "Data Availability" section was revised accordingly and the GigaDB dataset was cited.

Close
