# Supplementary material for: SimFFPE and FilterFFPE: improving structural variant calling in FFPE samples
Source: Gigascience. 2021 Sep 22;10(9):giab065. doi: 10.1093/gigascience/giab065 (PMC8458033; doi:10.1093/gigascience/giab065)
Supplement: giab065_Reviewer_1_Report_Original_Submission — Michael Linderman -- 5/19/2021 Reviewed [file giab065_reviewer_1_report_original_submission.pdf]

Reviewer Report

Title: SimFFPE and FilterFFPE: improving structural variant calling in FFPE samples

Version: Original Submission **Date:** 5/19/2021

Reviewer name: Michael Linderman

Reviewer Comments to Author:

In this paper the authors describe methods for simulating artifactual chimeric reads (ACRs) in FFPE samples and then filtering out those reads. These methods are implemented as a pair of R packages available via Bioconductor. The model for ACR creation is based on the random self assembly of single stranded DNA with complementary regions. The parameters for the simulation are derived from analysis of real FFPE samples. The authors evaluate the simulator via visual inspection of the pileup data and the filtering by calling SVs in simulated and real data using Delly, Lumpy and Manta. The truth set for the real data was constructed via manual review of the putative SVs. Evaluation using both the simulated and real data shows the FilterFFPE substantially improves SV calling PPV while maintaining sensitivity. Evaluation of the fidelity of the simulated data is more qualitative. The pileup images show strong visual similarity between the data produced by SimFFPE and real FFPE data, but there is no quantitative evaluation of the data produced by SimFFPE.
Specific comments/questions:
* Evaluating the fidelity of simulated NGS data in general is very difficult. And in this context many of the parameters you might compare in such an evaluation (i.e. those in Figure 2) were used to develop the tool. However, are there any other metrics you could use to quantitatively evaluate SimFFPE? For example, could a comparison between real and simulated FFPE data of the the fraction of improperly paired reads (or pairs mapping to different chromosomes) in tiled windows across the genome provide evidence of the fidelity of SimFFPE? Alternately are there experimental evaluations that might be possible, but would be beyond the scope of this work, which could be included as future work in the discussion?
* I had trouble understanding the manual curation process/results, and specifically category 3 SVs. My understanding is that this category comprises SVs that do not match a call in a FF sample and have less than 10 supporting reads. And that the authors manually reviewed 1,952 of these 46,829 SVs. Should I interpret Table S2 as 134/1,952 were actually true positives and 1,169/1,952 were considered ambiguous (and thus excluded)? Or is the "grey list" determined algorithmically? If the former, what fraction of the putative false positives (most of which, if I understood correctly, were not manually reviewed) would still be considered false positives (as opposed to "grey listed") had they been manually reviewed? Since there are so many putative false positives, it would seem that this category would have a significant impact on the results.
* Page 5 and Figure S11: I find the absolute scales in Figure S11 panels a-c hard to interpret since there is no context to know how many ACRs should have been excluded. I would advocate for reporting the fraction of ACR and non-ACR reads excluded. This would complement the results on Page 5, which if I understood correctly, are effectively the precision/PPV ("99.73% to 100.00% of the filtered reads were

ACRs (average: 99.96%)."). Is the sensitivity also reported somewhere? If not, I think including those stats in that paragraph would help the reader better understand FilterFFPE's effectiveness.
* I found the evaluation of the two filtering steps very interesting, and particularly the sentences on page 11 in the supplemental "This shows that the second filtering step of FilterFFPE has achieved its expected effect (improving sensitivity in case of low coverage or low SV frequency)." and on page 13 in the supplemental: "The second filtering step mainly improves sensitivity at low coverage or low SV frequency; thereby, the improvement in sensitivity applying the second filtering step is small in real data sets, while the improvement in PPV using only the first filtering step is more pronounced." The combination of those sentences prompted a number of questions:
    - Does this indicate that stage 1 alone excludes more than just ACRs, but that effect is attenuated at increased coverage?
    - What fraction of reads that would have been excluded by stage 1 only and retained when stage 2 is employed (if I understood correctly, theses are reads with unique breakpoints but no SRC region)? Or perhaps more useful for the reader, what are the precision and recall for excluding ACRs with the different filter configurations?
    - For the real data (with sufficient coverage) does looking for the SRC regions during filtering have either a neutral or actually a net negative effects on SV calling?
    - If there are indeed a relevant fraction of ACRs without SRC regions, is there a different mechanism that creates those reads? Is it the case that the SRC region is present but can't be detected (as suggested on page 5: "However, sequencing noise in ACRs may harm the correct detection of SRC regions.") or are there other error mechanisms at work?

I struggled to compare the effects of the two filter stages using Figures S12-S13, S14-S15, and S16-S17. I found myself flipping back and forth, but it is difficult to compare the values that way. Could Figures S14-S15 be integrated to show "No filter", "Stage 1 only" and "Stage 1 & 2" (i.e., three bars instead of two) to permit direct comparison? And perhaps something similar for Figures S16-S17? I recognize that doing the same for Figures S12-S13 might become unreadable. Is there another way to structure FigureS13 to more directly show the differences between the two filter configurations?
* The sections above prompted me to wonder if "stage 1" is a useful filter to apply prior to SV calling generally, not just for FFPE samples (at least with high coverage). How does stage 1 vs. stage 1 and 2 impact the sensitivity and PPV for SV calls in the FF samples? If stage 1 is an interesting filter "across the board", perhaps add a brief description of its utility to the discussion?
* My understanding is that FilterFFPE is removing reads and thus my initial (and likely naive) expectation is that sensitivity would be the same or decrease - not increase. If I understood correctly, the last paragraph of the results and Figure S16 indicates that after filtering all the callers are reporting fewer SVs. And from Page 7 my understanding is that all SVs (not just PASSing SVs) are considered in the analysis. If that is correct, does that indicate the callers are identifying more/different true positive SVs in the FilterFFPE data than in all the data?
* Page 7: "Despite developing a tool for realistic simulation of FFPE samples, it can be observed that sensitivity of the three SV calling tools Manta, Delly and Lumpy differed between simulated and real data. These discrepancies were mainly due to technical differences be- tween these data sets: our simulated samples were whole chromo- some sequencing data (mimicking WGS data since it is the ideal material for SV calling) while real samples contained WES data and had a shorter read length (150 bp in

simulated samples vs 90 bp in real samples)." I am curious why you did not try to generate simulated data similar to the real samples. My understanding is that SimFFPE supports both WGS and WES simulation.
Minor comments/questions:
* While the choice of Lumpy, Delly, and Manta seems very appropriate for the simulated WGS data, my understanding is that those methods are not necessarily designed for WES/targeted sequencing. I recognize though that the key comparisons here are within tools (with and without FilterFFPE) as opposed to absolute sensitivity, etc. Out of curiosity, do you think you would observe similar results for WES-specific CNV callers like XHMM, etc.?
* When simulating WES data, how is the effect of the WES capture technology modeled?
* Page 3: "In real data, we observed that some read pairs from adjacent ACFs both align to the same genomic locus. We found out that this is a special phenomenon arising from the enzymatic fragmentation of some adjacent ACFs…" I had trouble following the phenomenon being described in this section. Perhaps a naive question, but is this a function of the specific library preparation method, or does this occur generally in FFPE samples? How does SimFFPE model the fragmentation location?
* I found Figure 5 confusing at first. Based on the convergence of arrows at "SV calling" I initially thought that the simulated data was used during evaluation of the real FFPE and FF samples. But if I understood correctly those are entirely separate analyses. If that is indeed correct, I would suggest having separate a "SV calling" box for the for the simulated and real data workflows.
* I find Figure 8 difficult to interpret since you have to match the dots manually based on the very small text. Is the intent for the reader to look at the shift of individual points, or to look at the shift in the overall distribution? If the latter, is there an alternate visualization of the distribution? Or perhaps overlay a cross with the means, quartiles, etc.?
* Page 7: I would advocate for including the standard deviation along with the means (or the range of changes observed) to provide additional context for interpreting the changes in sensitivity and PPV before and after applying FilterFFPE.

Level of Interest

Please indicate how interesting you found the manuscript: Choose an item.

Quality of Written English

Please indicate the quality of language in the manuscript: Choose an item.

Declaration of Competing Interests

Please complete a declaration of competing interests, considering the following questions:

- Have you in the past five years received reimbursements, fees, funding, or salary from an organisation that may in any way gain or lose financially from the publication of this manuscript, either now or in the future?

- Do you hold any stocks or shares in an organisation that may in any way gain or lose financially from the publication of this manuscript, either now or in the future?
- Do you hold or are you currently applying for any patents relating to the content of the manuscript?
- Have you received reimbursements, fees, funding, or salary from an organization that holds or has applied for patents relating to the content of the manuscript?
- Do you have any other financial competing interests?
- Do you have any non-financial competing interests in relation to this paper?

If you can answer no to all of the above, write 'I declare that I have no competing interests' below. If your reply is yes to any, please give details below.

I declare that I have no competing interests

I agree to the open peer review policy of the journal. I understand that my name will be included on my report to the authors and, if the manuscript is accepted for publication, my named report including any attachments I upload will be posted on the website along with the authors' responses. I agree for my report to be made available under an Open Access Creative Commons CC-BY license (http://creativecommons.org/licenses/by/4.0/). I understand that any comments which I do not wish to be included in my named report can be included as confidential comments to the editors, which will not be published.

Choose an item.

To further support our reviewers, we have joined with Publons, where you can gain additional credit to further highlight your hard work (see: https://publons.com/journal/530/gigascience). On publication of this paper, your review will be automatically added to Publons, you can then choose whether or not to claim your Publons credit. I understand this statement.

Yes Choose an item.
