# Supplementary material for: SimFFPE and FilterFFPE: improving structural variant calling in FFPE samples
Source: Gigascience. 2021 Sep 22;10(9):giab065. doi: 10.1093/gigascience/giab065 (PMC8458033; doi:10.1093/gigascience/giab065)
Supplement: giab065_Reviewer_2_Report_Original_Submission — Binay Panda -- 6/16/2021 Reviewed [file giab065_reviewer_2_report_original_submission.pdf]

Reviewer Report

Title: SimFFPE and FilterFFPE: improving structural variant calling in FFPE samples

Version: Original Submission **Date:** 6/16/2021

Reviewer name: Binay Panda

Reviewer Comments to Author:

GIGA-D-21-00120
SimFFPE and FilterFFPE: improving structural variant calling in FFPE samples
The paper 'SimFFPE and FilterFFPE: improving structural variant calling in FFPE samples' describes a tool created by Wei et al, FilterFFPE, that identifies spurious split reads generated due to sample preparation by formalin fixing and paraffin embedding (FFPE). In doing so, false positive structural variants called due to support from these spurious reads were observed to be eliminated in simulated data. Further, Wei et al describe another tool they created called SimFFPE, which is a read simulator specifically designed for samples prepared using the FFPE approach. Both tools are R packages.
Overall, the paper is well-written and the analyses are also well-done, and such tools are useful to the field. I have two major comments for the authors to address and expand on. These need to be addressed before the manuscript is accepted for publication.
Major comments
1. On pages 4-5, under section 'Simulating ACFs', the authors mention a higher probability of SCRs forming between adjacent chromosomal regions or within the same chromosome, based on 18 real FFPE samples. However, the Figure 2 indicates the probability of SCRs on the same chromosome as 43%, and that on different chromosomes as 57%.
2. Figure 8 shows sample-wise and tool-wise differences in relationship between positive predictive values and sensitivity of SV detection, using real data. It is not clear what causes sample-to-sample differences in this relationship. Can the authors elaborate further?

Level of Interest

Please indicate how interesting you found the manuscript: Choose an item.

Quality of Written English

Please indicate the quality of language in the manuscript: Choose an item.

Declaration of Competing Interests

Please complete a declaration of competing interests, considering the following questions:

- Have you in the past five years received reimbursements, fees, funding, or salary from an organisation that may in any way gain or lose financially from the publication of this manuscript, either now or in the future?
- Do you hold any stocks or shares in an organisation that may in any way gain or lose financially from the publication of this manuscript, either now or in the future?
- Do you hold or are you currently applying for any patents relating to the content of the manuscript?
- Have you received reimbursements, fees, funding, or salary from an organization that holds or has applied for patents relating to the content of the manuscript?
- Do you have any other financial competing interests?
- Do you have any non-financial competing interests in relation to this paper?

If you can answer no to all of the above, write 'I declare that I have no competing interests' below. If your reply is yes to any, please give details below.

I declare that I have no competing interests.

I agree to the open peer review policy of the journal. I understand that my name will be included on my report to the authors and, if the manuscript is accepted for publication, my named report including any attachments I upload will be posted on the website along with the authors' responses. I agree for my report to be made available under an Open Access Creative Commons CC-BY license (http://creativecommons.org/licenses/by/4.0/). I understand that any comments which I do not wish to be included in my named report can be included as confidential comments to the editors, which will not be published.

Choose an item.

To further support our reviewers, we have joined with Publons, where you can gain additional credit to further highlight your hard work (see: https://publons.com/journal/530/gigascience). On publication of this paper, your review will be automatically added to Publons, you can then choose whether or not to claim your Publons credit. I understand this statement.

Yes Choose an item.
